# Multisite PCET with photocharged carbon nitride in dark

**DOI:** 10.1002/EXP.20210063

**Published:** 2021-12-16

**Authors:** Stefano Mazzanti, Clara Schritt, Katharina ten Brummelhuis, Markus Antonietti, Aleksandr Savateev

**Affiliations:** ^1^ Max‐Planck Institute of Colloids and Interfaces, Department of Colloid Chemistry Research Campus Golm Potsdam Germany; ^2^ Institut für Chemie und Biochemie Freie Universität Berlin Berlin Germany

**Keywords:** carbon nitride, PCET, photocatalysis, photocharging, poly(heptazine imide), thermochemistry

## Abstract

A combination of photochemistry and proton coupled electron transfer (PCET) is a primary strategy employed by biochemical systems and synthetic chemistry to enable uphill reactions under mild conditions. Degenerate nanometer‐sized n‐type semiconductor nanoparticles (SCNPs) with the Fermi level above the bottom of the conduction band are strongly reducing and act more like metals than semiconductors. Application of the degenerate SCNPs is limited to few examples. Herein, we load microporous potassium poly(heptazine imide) (K‐PHI) nanoparticles with electrons (e^‒^) and charge balancing protons (H^+^) in an illumination phase using sacrificial agents. e^‒^/H^+^ in the K‐PHI nanoparticles are weakly bound and therefore could be used in a range of PCET reactions in dark, such as generation of aryl radicals from aryl halides, ketyl radicals from ketones, and 6e^‒^/6H^+^ reduction of nitrobenzene to aniline. The integration of several features that until now were intrinsic for plants and natural photosynthesis into a transition metal free nanomaterial composed of abundant elements (C, N, and K) offers a powerful tool for synthetic organic chemistry.

## Graphical abstract

Nature relies on the exploitation of sunlight and hydrogen ions to enable uphill organic reactions, namely photosynthesis. Herein, a biomimetic organic semiconductor composed of abundant elements (C, N, and K) uses this strategy to accumulate light energy and store hydrogen ions as powerful tool for synthetic organic chemistry in the flask.

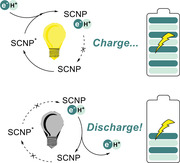

## 1 INTRODUCTION

Photochemistry and proton coupled electron transfer (PCET) – combination of these two approaches is the only efficient strategy to enable uphill reactions under mild conditions, employed both in natural photosynthesis and photocatalysis.^[^ ^1^, ^2^ ^]^ Indeed, electronic energy is insensitive to temperature change as inferred from Boltzmann constant, 8.5 × 10^−5^ eV K^−1^, while PCET reactions are characterized by lower activation energy compared to stepwise electron transfer (ET)/proton transfer (PT) as it avoids formation of high‐energy intermediates.^[^ ^3^, ^4^, ^5^ ^]^

**FIGURE 1:**
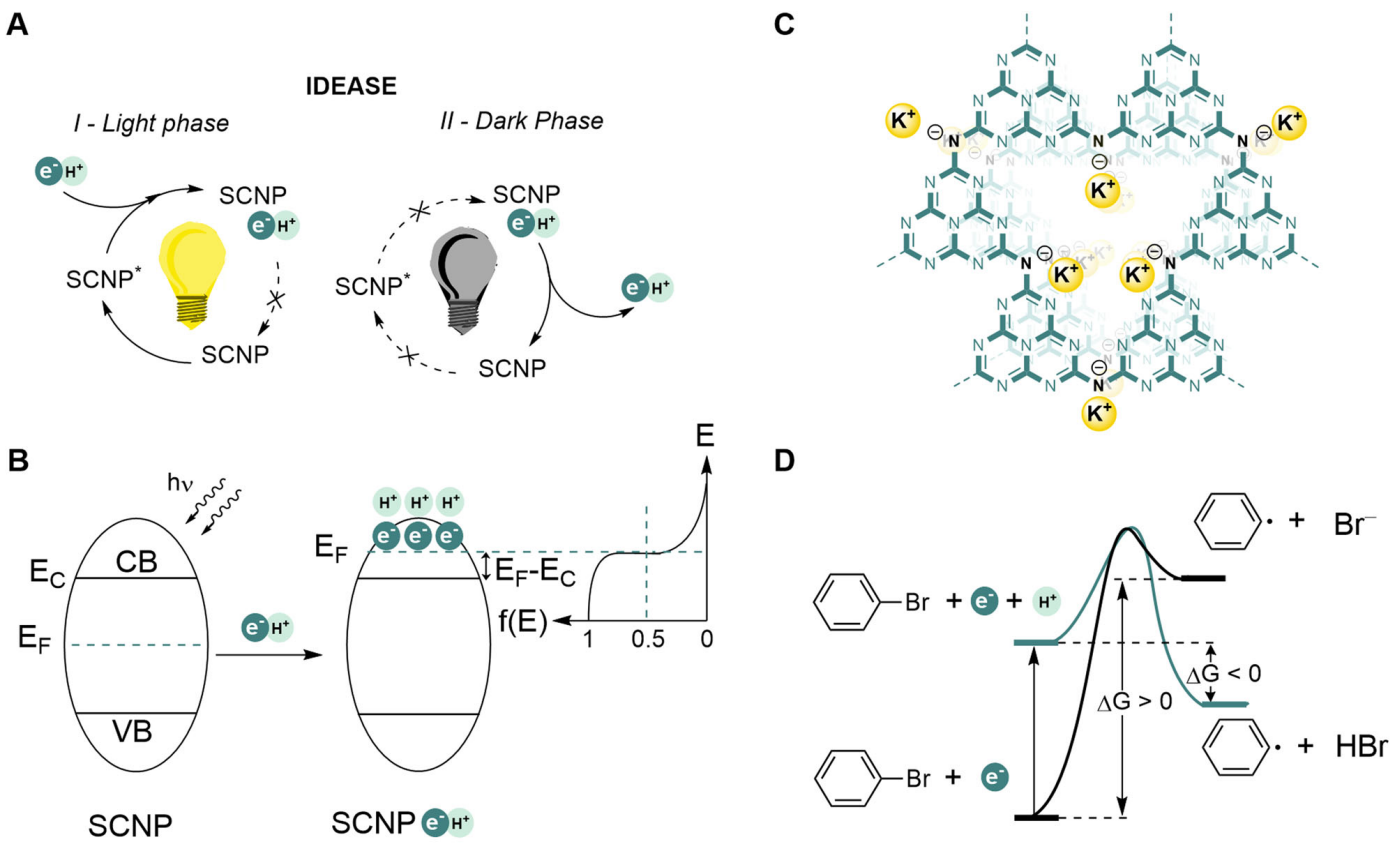
Background and concept of this work. (A) Decoupling of light and dark phases in photocatalysis through IDEASE followed by PCET using e^‒^/H^+^ stored in SCNP. (B) Irradiation of the SCNP in the presence of e^‒^/H^+^ donors triggers accumulation of electrons in the CB and H^+^ at the surface of the SCNP. (C) Structure of K‐PHI. (D) Generation of aryl radicals via ET and MS‐PCET

It has been known as early as 1980 that n‐ and p‐type semiconductor nanoparticles (SCNPs) are able to accumulate either electrons or holes.^[^ ^6^, ^7^, ^8^ ^]^ The process of SCNPs “charging” is triggered by irradiation with light in the presence of sacrificial agents, while the stored charges can then be employed under dark conditions for either reduction or oxidation of the substrates.^[^ ^8^, ^9^ ^]^ In the last decade, this area of research has been flourishing with ingenious terms and acronyms, such as “dark photocatalysis,”^[^ ^10^, ^11^ ^]^ IDEASE (illumination driven electron accumulation and exploitation, Figure 1A),^[^ ^12^ ^]^ memory photocatalysis^[^ ^13^, ^14^ ^]^ and round‐the‐clock photocatalysis.^[^ ^15^ ^]^ Indeed, the concept is intriguing as it offers a way to decouple light and dark phases in photocatalysis, mimics natural photosynthesis, and potentially explores unknown pathways in organic synthesis.^[^ ^12^, ^16^ ^]^ In the context of this work, hereafter we narrow discussion to n‐type SCNPs with an excess of electrons in the conduction band (CB). A remarkable feature of such charged (degenerately or heavily doped) SCNPs, is higher reduction power due to the shift (*E*_F_ − *E*_C_) of the Fermi level above the bottom of the CB (Figure 1B),^[^ ^17^ ^]^ which makes them more similar to reductive metals than semiconductors.^[^ ^18^ ^]^ For example, the conductivity of TiO_2_ doped in this way increases six orders of magnitude.^[^ ^19^ ^]^ The shift of the Fermi level could be as high as ‒1 eV, for example, for CdS nanoparticles with the diameter of 1.5 nm, but it vanishes quickly as the diameter of the SCNP exceeds 5–10 nm.^[^ ^20^ ^]^

In such degenerate nanoparticles of inorganic semiconductors, the negative charge caused by the excess of electrons in the CB has to be compensated by protons (H^+^), which can collectively be used in reductive PCET.^[^ ^21^ ^]^ In addition, negative charges could be compensated by alkali, alkaline earth metal cations, and even bulky organic cations, such as tetrabutylammonium and decamethylcobaltocenium, which are then stored in the double layer.^[^ ^22^ ^]^

Electrons and protons stored in metal oxide nanoparticles have been employed in reduction of organic substrates in the dark. Thus, Mayer et al. performed 2e^‒^/2H^+^ reduction of C═O, C═N, and N═N bonds.^[^ ^23^ ^]^ Zhang and Wang employed amorphous TiO_2_ loaded with e^‒^/H^+^ in hydrogenation of 5‐hydroxymethylfurfural to 2,5‐bis(hydroxymethyl)furan^[^ ^24^ ^]^ as well as in H_2_ evolution at MoS_2_ co‐catalyst.^[^ ^25^ ^]^ Metal oxides with stored e^‒^/H^+^ are also key intermediates in deoxygenation of epoxides^[^ ^26^ ^]^ and reduction of aldehydes to alcohols,^[^ ^27^ ^]^ but under continuous light irradiation.

Our own results suggest that SCNPs of potassium poly(heptazine imide) (K‐PHI, Figure 1C), a special member of the carbon nitride materials family,^[^ ^28^ ^]^ loaded with e^‒^/H^+^ (abbreviated as K‐PHI(e^‒^/H^+^) hereafter), are involved into the photocatalytic cycle of α,β‐unsaturated ketones reductive dimerization to cyclopentanoles under continuous light irradiation.^[^ ^29^ ^]^ K‐PHI(e^‒^/H^+^) mediates generation of dichloromethyl radical from chloroform followed by Giese‐type addition to α,β‐unsaturated ketones,^[^ ^30^ ^]^ and enables condensation of α,β‐unsaturated ketones with tetrahydroisoquinolines.^[^ ^31^ ^]^ The really high activity of K‐PHI(e^‒^/H^+^) in PCET reactions in dark and under continuous light irradiation obviously stems from a combination of its properties: 1) the system has a conjugated microporous structure with a pore diameter of ca. 0.8 nm, formed around columns of K^+^ ions, hosting large amounts of both e^‒^ and H^+^. Therefore, the material can store electrons both inside its channels and on the outside surface of the particle.^[^ ^32^, ^33^ ^]^ 2) The highly positive potential of the valence band (+ 2.2 V vs. NHE)^[^ ^34^ ^]^ facilitates fast (*k* ∼ 10^5^–10^12^ s^−1^)^[^ ^11^, ^35^ ^]^ quenching of holes at the expense of a sacrificial electron donor. 3) Finally, potassium ions, which are intrinsic part of the K‐PHI structure, are able to stabilize efficiently the excessive negative charge by forming the intercalation band.^[^ ^16^ ^]^

Mayer, Gamelin et al. showed that the number of electrons added per metal oxide nanoparticle is proportional to *r* ^3^, while density, ∼ 1–2·10^20^ [e^‒^] cm^−3^, is constant in the range of radii *r* = 1.5–6 nm.^[^ ^22^, ^36^ ^]^ A higher degree of K‐PHI photoloading of up to 0.5e^‒^/0.5H^+^ per unit cell (∼ 10^21^ [e^‒^] cm^−3^), can be achieved even for particles with the diameter > 100 nm,^[^ ^37^ ^]^ which is only possible when e^‒^/H^+^ are stored in the micropores.

Plenty of reports using K‐PHI nanoparticles in net‐reductive reactions and high capacity of stored electrons prompted us to investigate K‐PHI(e^‒^/H^+^) as multisite (MS) PCET reductants in dark. In MS‐PCET approach, electrons are stored in the π‐conjugated structure of K‐PHI, while protons are located in the micropores, between the layers separated by 0.3 nm or on the surface. Therefore, unlike conventional hydrogen atom transfer catalysts,^[^ ^38^, ^39^, ^40^ ^]^ e^‒^/H^+^ from K‐PHI(e^‒^/H^+^) are transferred to the substrate from different sites.

Aryl halides are synthetically useful precursors of aryl radicals in photoredox catalysis,^[^ ^41^, ^42^ ^]^ and can be obtained upon photolysis with UV light.^[^ ^43^, ^44^ ^]^ One‐electron reduction of aryl halides to aryl radicals occurs at potential of ‒1 to ‒2 V vs. SCE,^[^ ^45^ ^]^ which makes ET in this case ca. + 23 to + 46 kcal mol^−1^ endergonic, but it can be accomplished employing strongly reducing photoredox catalysts.^[^ ^46^, ^47^ ^]^ Alternatively, MS‐PCET, in which e^‒^ is transferred to the aryl moiety, while H^+^ is added to Br^‒^, is ‒5.2 kcal mol^−1^ exergonic (Figure 1D, see Supporting Information for thermochemical calculations).^[^ ^48^, ^49^ ^]^

In this work, we report a two‐step method to generate aryl radicals from the corresponding aryl halides. In the first step, we load K‐PHI with e^‒^/H^+^ under light irradiation in the presence of the sacrificial donor. In the second step, aryl halide is reduced via MS‐PCET by e^‒^/H^+^ stored in K‐PHI nanoparticles in dark. Thermochemical calculations suggest that e^‒^/H^+^ in K‐PHI are weakly bound, which makes them useful for reduction of classes of organic compounds, such as generation of ketyl radicals and even 6e^‒^/6H^+^ reduction of nitro compounds to anilines in dark.

## 2 METHODS

### 2.1 K‐PHI synthesis

Potassium poly(heptazine imide) (K‐PHI) has been prepared according to the reported procedure.^[^ ^32^ ^]^ Mixture of lithium chloride (3.71 g), potassium chloride (4.54 g), and 5‐aminotetrazole (1.65 g) was ground in ball mill for 5 min at the shaking rate 25 s^−1^. Reaction mixture was transferred into a porcelain crucible and covered with a lid. The crucible was placed in the oven and heated under constant nitrogen flow (15 L min^−1^) and atmospheric pressure. The temperature increased from 25 °C to 600 °C within 4 h followed by annealing at 600 °C for 4 h. After completion of the heating program, the crucibles were allowed to cool slowly to room temperature under nitrogen flow. The crude product was removed from the crucible, washed with deionized water (100 ml) for 3 h. Solid was separated by centrifugation (10 min, 4000 rpm) followed by washing with deionized water and centrifugation (3 × 2 ml, centrifugation at 13,000 rpm for 3 min each time). The solid was dried in a vacuum oven (20 mbar) at 50 °C overnight.

### 2.2 Cyclic voltammetry (CV)

Measurements were performed in a glass single‐compartment electrochemical cell. Glassy carbon (diameter 3 mm) was used as a working electrode (WE), Ag wire in AgNO_3_ (0.01 M) as a reference electrode (RE), Pt wire as a counter electrode. Each compound was studied in a 30 mM concentration in a 0.1 M tetrabutylammonium perchlorate and DMSO electrolyte solution (10 ml). Before voltammograms were recorded, the solution was purged with Ar, and an Ar flow was kept in the headspace volume of the electrochemical cell during CV measurements. A potential scan rate of 0.050 V s^−1^ was chosen, and the potential window ranging from +1.5 to −2.5 V (and backward) was investigated. CV was performed under room‐temperature conditions (∼ 20–22 °C). Values have been converted vs SCE using ferrocene as internal standard.

^1^H and ^13^C NMR spectra were recorded on Agilent 400 MHz (at 400 MHz for Protons and 101 MHz for Carbon‐13). Chemical shifts are reported in ppm versus solvent residual peaks: DMSO*‐d_6_* 2.50 ppm (^1^H NMR), 39.52 ppm (^13^C NMR)

Mass spectral data were obtained using Agilent GC 6890 gas chromatograph, equipped with HP‐5MS column (inner diameter = 0.25 mm, length = 30 m, and film = 0.25 μm), coupled with Agilent MSD 5975 mass spectrometer (electron ionization).

## 3 RESULTS

Information related to synthesis and characterization of K‐PHI nanoparticles is given in the supplementary information (Figure S1). For reduction of aryl halides via MS‐PCET, we developed a two‐step procedure: 1) irradiation of the reaction mixture with blue light for time τ_1_ at temperature *T*_1_ in the presence of a sacrificial agent to load K‐PHI with e^‒^/H^+^; 2) addition of an aryl halide to K‐PHI(e^‒^/H^+^) and maintaining the reaction mixture in dark for time τ_2_ at temperature *T*_2_. While a complete data set of the reaction conditions optimization is given in Tables S1–S8, Table 1 highlights the most important of our findings with optimized conditions in the heading.

**TABLE 1:** Optimization of reaction conditions

**Entry**	**1a (mmol)**	**Semiconductor**	**Amine**	**Solvent**	***τ*_1_ (h)**	***τ*_2_ (h)**	***T*_1_ (°C)**	***T*_2_ (°C)**	**Yield (%)**
1^†^	0.025	K‐PHI (20 mg)	TEA (350 μl)	DMSO (1 ml)	25	20	RT	50	6
2^†^	0.025	K‐PHI (40 mg)	TEA (350 μl)	DMSO (1 ml)	25	20	RT	50	15
3^†^	0.025	K‐PHI (80 mg)	TEA (350 μl)	DMSO (1 ml)	25	20	RT	50	22
4^†^	0.025	K‐PHI (20 mg)	TEA (350 μl)	DMSO (1 ml)	16	5	RT	80	14
5^‡^	0.025	K‐PHI (20 mg)	DIPEA (140 μl)	DMSO (1 ml)	22	19	RT	80	67
6^‡^	0.025	K‐PHI (80 mg)	DIPEA (140 μl)	DMSO (1 ml)	22	19	RT	80	31
7^‡^	0.025	K‐PHI (80 mg)	DIPEA (140 μl)	DMSO (3 ml)	22	20	RT	80	71
8^‡^	0.05	K‐PHI (80 mg)	DIPEA (560 μl)	DMSO (3 ml)	24	20	RT	80	100
9^‡^	0.05	K‐PHI (80 mg)	DIPEA (560 μl)	DMSO (3 ml)	24	20	RT	RT	7
10^§^	0.05	K‐PHI (80 mg)	DIPEA (560 μl)	DMSO (3 ml)	24	20	RT	80	Traces
11^‡^	0.05	None	DIPEA (560 μl)	DMSO (3 ml)	24	20	RT	80	Traces
12^‡^	0.05	Na‐PHI (80 mg)	DIPEA (560 μl)	DMSO (3 ml)	24	20	RT	80	61
13^‡^	0.05	mpg‐CN (80 mg)	DIPEA (560 μl)	DMSO (3 ml)	24	20	RT	80	12
14^‡^	0.05	TiO_2_ (80 mg)	DIPEA (560 μl)	DMSO (3 ml)	24	20	RT	80	Traces
15^‡,¶^	0.05	K‐PHI (80 mg)	DIPEA (560 μl)	DMSO (3 ml)	24	/	RT	/	100
16^‡,**^	0.05	K‐PHI (80 mg)	DIPEA (560 μl)	DMSO (3 ml)	24	20	RT	80	49

^†^Blue light (5 cm distance, 49 mW cm^−2^).

^‡^Blue light (1 cm distance, 370 mW cm^−2^).

^§^Vial wrapped in aluminum foil (no light).

^¶^Reaction performed in one step (only light).

^**^Reaction performed using recycled K‐PHI.

We started screening the reaction conditions with triethylamine (TEA) as a sacrificial e^‒^/H^+^ donor and DMSO as solvent. Carbonyl compounds bearing halogen atom at the aromatic ring are a convenient class of organic molecules to study selectivity of PCET reaction since the reduction potentials of C═O and C‒Hal functionalities overlap and are in the range ‒1 to ‒2 V vs SCE.^[^ ^45^ ^]^ 4‐bromoacetophenone (**1a**) was selected as the model aryl halide to add in the step 2. Time of the light phase was chosen to be 25 h and temperature *T*_1_ = 25 °C, while the parameters of the dark phase were *τ*_2_ = 20 h and *T*_2_ = 50 °C. Thus, acetophenone **2a** was obtained with 6% yield (Table 1, entry 1). By increasing the amount of K‐PHI the yield reached 22% (entry 2–3). We found that crucial parameter to accelerate the reaction was raising the temperature in the dark phase to *T*_2_ = 80 °C (entry 4). Screening the amount and type of amine (Table S2), led to DIPEA as the optimum sacrificial electron donor (Table 1, entry 5). Taking into account the high extinction coefficient of carbon nitrides, > 10^5^ cm^−5^,^[^ ^50^ ^]^ further improvement in the yield of **2a** was achieved by decreasing optic density of the solution and increasing light intensity (Table 1, entry 7). After further screening of the amount of DIPEA and the duration of *τ*_1_ and *τ*_2_ (Tables S5,S6), we achieved 100% yield of **2a** (Table 1, entry 8). Control experiments confirmed that photocharging of K‐PHI proceeds only upon irradiation with light (Table 1, entry 10–11). Under optimized conditions ionic carbon nitrides,^[^ ^51^ ^]^ such as Na‐PHI, gave **2a** in 61% yield, while mesoporous graphitic carbon nitride (mpg‐CN) gave **2a** in only 12% yield (entry 12–13), which underlines essential role of the negatively charged framework and the larger microporous structure of ionic carbon nitrides for the storage of protons (Figure S2).^[^ ^32^, ^52^ ^]^ Commercial TiO_2_ rutile did not give **2a** (entry 14), which we explain by low amount of (e^‒^/H^+^) stored in this material as surface area and morphology have not been optimized. In the control experiment, K‐PHI also reduces **1a** under continuous illumination with light (entry 15). The color of K‐PHI(e^‒^/H^+^) dispersion, which arises from the intercalation band,^[^ ^16^ ^]^ depends on the solvent and is the most pronounced in case of using DMSO (Figure S3A). Remarkably, K‐PHI(e^‒^/H^+^) is able to reduce **1a** even after storage for 7 days under O_2_ free conditions (Table S9, entry 1–3).^[^ ^53^ ^]^ In the second run, recovered K‐PHI nanoparticles gave **2a** in 49% yield, implying that the material is stable and does not degrade (Table 1, entry 16).

**FIGURE 2:**
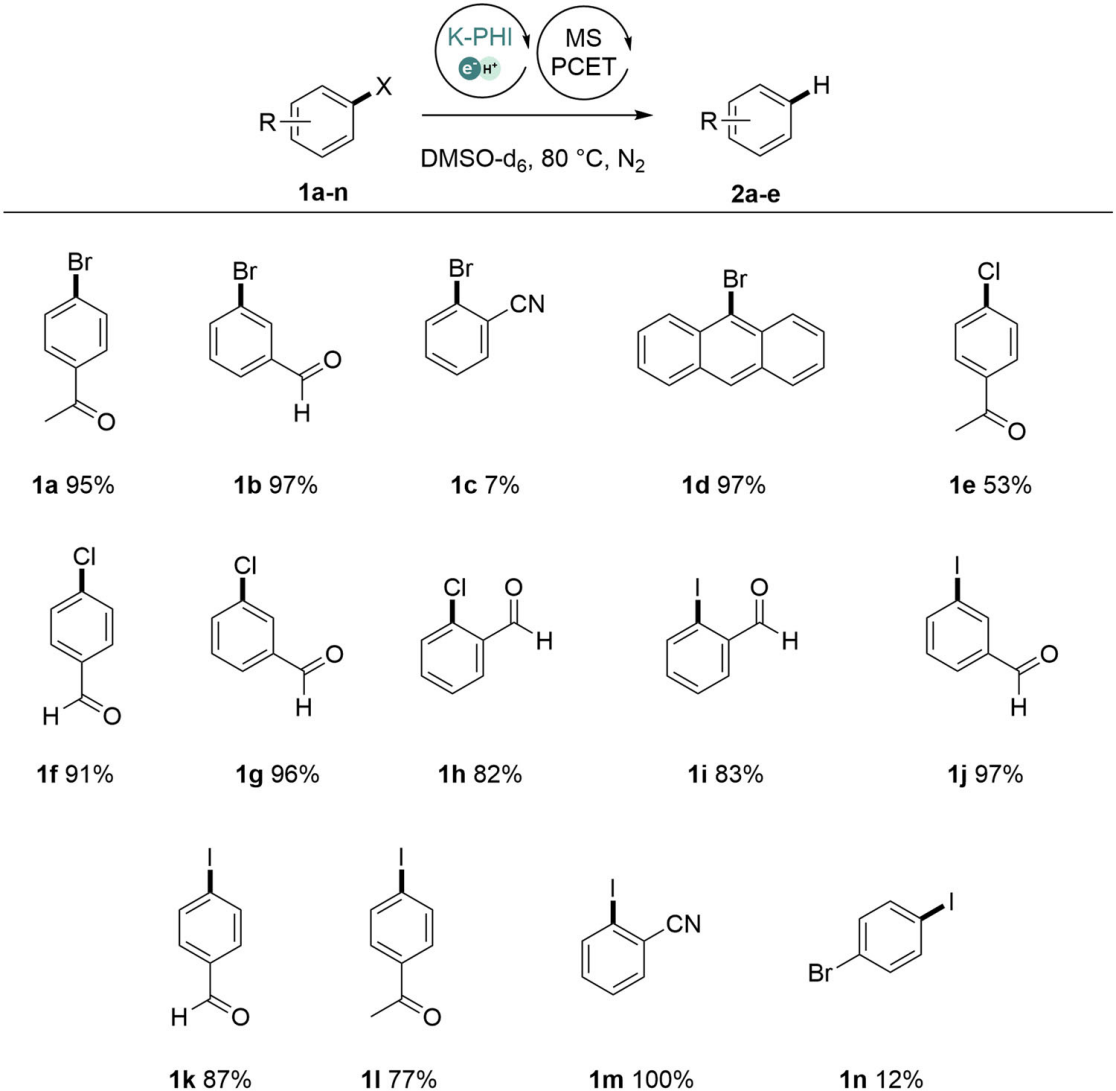
Scope of aryl halides in MS‐PCET using K‐PHI(e^‒^/H^+^). Conditions: (1) 80 mg K‐PHI, 3 ml DMSO‐d_6_, Blue light (370 mW⋅ cm^−2^), 24 h (*τ*_1_), RT (*T*_1_); (2) 0.05 mmol aryl halide (**1a**‐**n**) in 1 ml of DMSO‐d_6_, 20 h (*τ*_2_), 80 °C (*T*_2_). See standard procedure in ESI for further details. Yields determined by ^1^H NMR with internal standard

We applied the optimized conditions to generate aryl radicals from other aryl halides. Fourteen substrates were converted into the corresponding aromatics in 7%–100% yield (Figure 2).

Aryl chlorides (**1e**–**1h**), which are considered to be less reactive substrates,^[^ ^54^ ^]^ due to higher BDFE values of C‒Cl bond compared to C‒Br bond, were reduced as well, pleasingly with yields comparable to that of aryl bromides and iodides. The yield of benzonitrile from *ortho*‐cyano substituted aryl halides (**1c**, **m**, **s**) depends on the nature of the leaving group and follows the order I > Br > Cl. In a competitive study using **1n**, C─I bond was selectively cleaved. At this point, we could not derive a strong correlation between the half reduction potential (E_p1/2_) of the aryl halides, BDE value of the C‒Hal bond, and the yield of the aromatic compound (Figure S5B). It implies that not only reducibility of aryl halides expressed through the BDE values and E_p1/2_ define reactivity of the substrate, but other factors as well. In general, among the tested substrates (**1a**–**1t**), those bearing formyl and acetyl groups have been reduced with excellent yields, which we take as an indication for improved interaction of the substrate with the surface of K‐PHI. Such conclusion agrees with the results of hydroxymethylfurfural adsorption at TiO_2_.^[^ ^24^ ^]^ Analysis of half reduction potentials of aryl halides strongly supports PCET mechanism since all substrates have E_p1/2_ < ‒1.7 V vs SCE (Table S10), which is ca. ‒1 V more negative than the potential of the CB (‒0.75 V vs SCE).^[^ ^34^ ^]^ Therefore, reduction of aryl halides is too endergonic to proceed via stepwise ET/PT pathway under the studied conditions.

**FIGURE 3:**
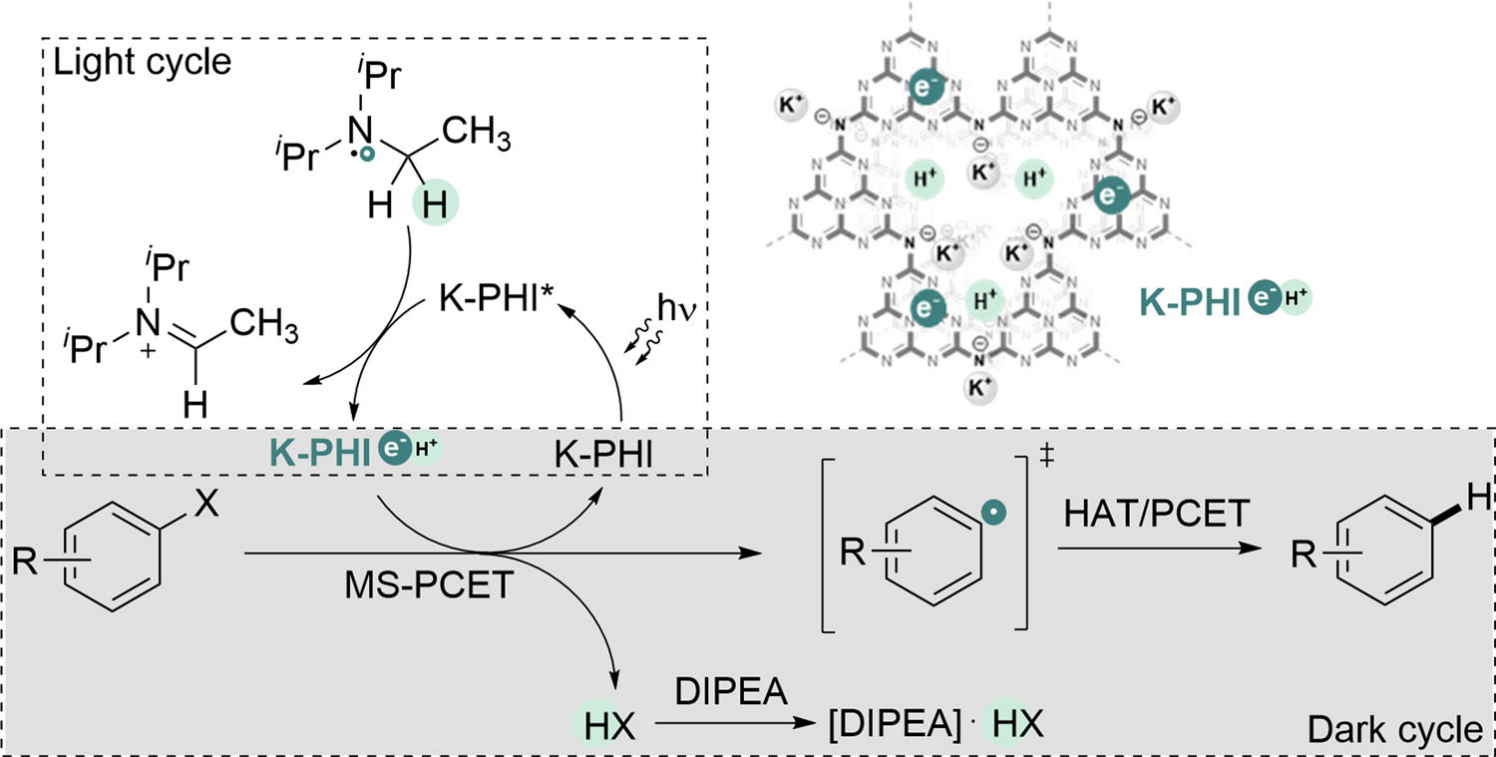
A proposed mechanism of aryl halides reduction via MS‐PCET using K‐PHI(e^‒^/H^+^). K‐PHI* denotes excited state of K‐PHI with photogenerated electron‐hole pairs

The mechanism of the two‐step generation of aryl radicals is shown in Figure 3. Absorption of photon by K‐PHI leads to the formation of K‐PHI* excited state with the electron and hole located in CB and VB respectively. Taking into account highly positive VB potential of K‐PHI (+ 2.2 V vs NHE) and earlier reports on reductive MS‐PCET using tertiary amines, such N(*n*‐Bu)_3_,^[^ ^55^ ^]^ charging of the SCNP proceeds via stepwise 1e^‒^ oxidation of DIPEA followed by injection of a second electron into the CB (current doubling) and transfer of H^+^ from the α‐carbon atom to K‐PHI nanoparticle. As a result, DIPEA loses two e^‒^, one H^+^ and is converted into iminium cation. Excessive negative charge of the photodoped K‐PHI nanoparticle is stabilized both by H^+^ and iminium cation. In the dark cycle, transfer of H^+^ to the halide (X) and e^‒^ to the aryl moiety leads to the formation of HX and the aryl radical. Given that BDFE[C‒H], for example, in benzene is 104.4 kcal mol^−1^,^[^ ^49^ ^]^ once formed aryl radical abstracts e^‒^/H^+^(or H^•^) either from K‐PHI(e^‒^/H^+^) or other components of the reaction medium.

Thermochemical calculations suggest that e^‒^/H^+^ are weakly bound to K‐PHI – BDFE[K‐PHI(e^‒^/H^+^)] is < 5.2 kcal mol^−1^ (see Supporting Information). Such BDFE value is much lower than that of the ketyl radicals (ca. 26–30 kcal mol^−1^).^[^ ^2^, ^56^ ^]^ It is also lower than BDFE of the superoxide radical (42.7 kcal mol^−1^)^[^ ^57^ ^]^ or H_2_O_2_ (79.6 kcal mol^−1^),^[^ ^57^ ^]^ which are the intermediates of O_2_ reduction to H_2_O_2_. Therefore, charged K‐PHI(e^‒^/H^+^) nanoparticles are strong reductants. Indeed, such conclusions agree with earlier reports of using charged K‐PHI(e^‒^/H^+^) or similar carbon nitride materials in the reduction of O_2_ to H_2_O_2_ in dark.^[^ ^58^, ^59^ ^]^

**FIGURE 4:**
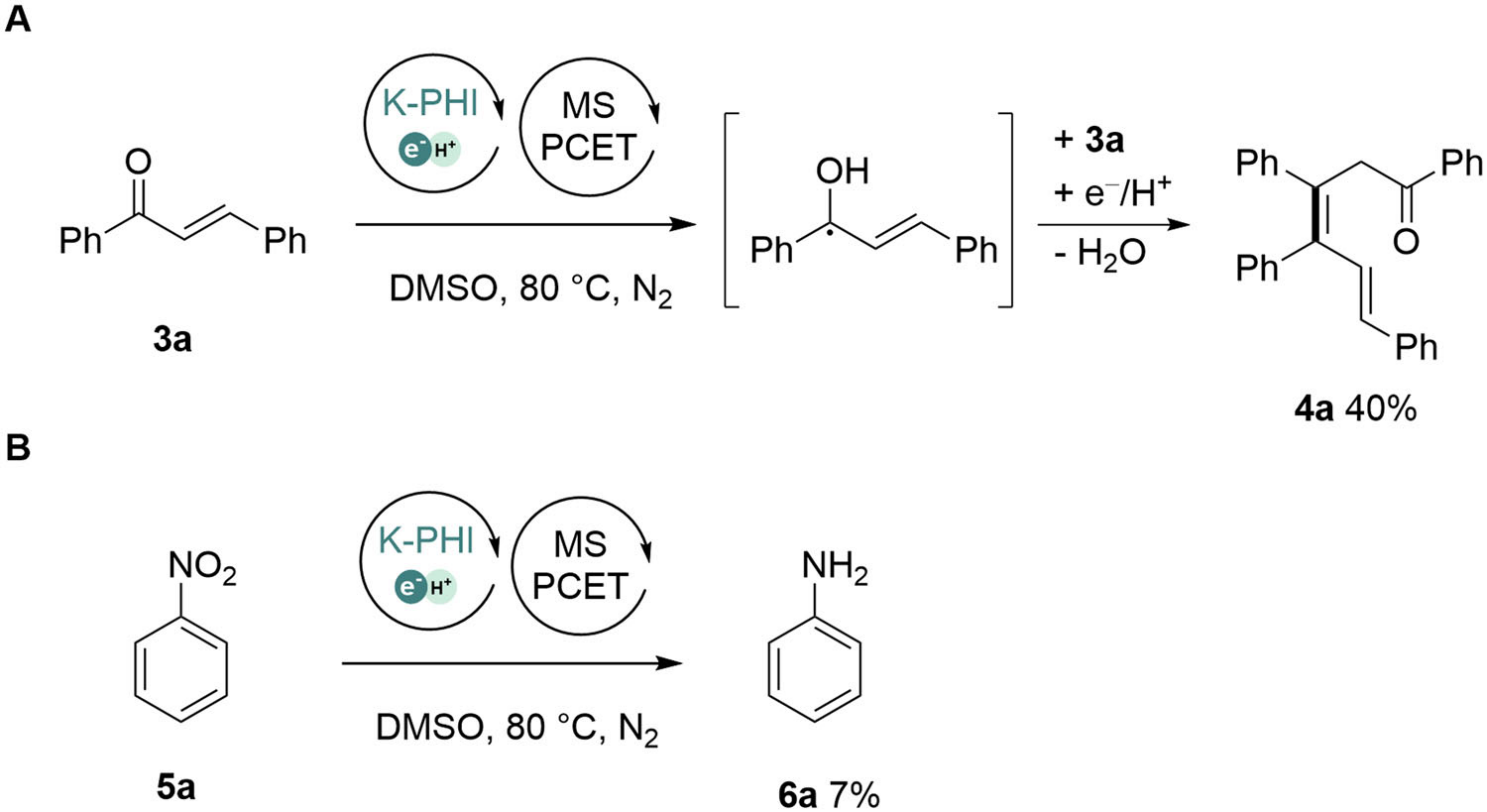
Scope of the MS‐PCET reactions mediated by K‐PHI(e^‒^/H^+^) in dark. (A) Generation of the ketyl radical followed by addition to the β‐carbon atom of chalcone **3a** and formation of the dienone **4a**. (B) Reduction of nitrobenzene to aniline via MS‐PCET using K‐PHI(e^‒^/H^+^) in dark. Conditions: (1) 80 mg K‐PHI, 3 ml DMSO, Blue light (370 mW⋅ cm^−2^), 24 h (*τ*_1_), RT (*T*_1_); (2) 0.05 mmol substrate (**3a** or **5a** in 1 ml of DMSO, 20 h (*τ*_2_), 80 °C (*T*_2_). See standard procedure in ESI for further details. Yields determined by GC‐MS

We proved experimentally that K‐PHI(e^‒^/H^+^) nanoparticles reduce chalcone to the ketyl radical, which followed by coupling with the second chalcone molecule, gives dienone **4a** with a yield of 40% (under non‐optimized conditions, Figure 4A). Finally, reduction of nitrobenzene is known to terminate at the step of diazo‐ and/or azoxy‐compounds,^[^ ^60^ ^]^ while 6e^‒^/6H^+^ reduction to aniline can be accomplished using K‐PHI nanoparticles under continuous light irradiation (in this case K‐PHI(e^‒^/H^+^) is constantly recovered) in the presence of formic acid as sacrificial donor of electrons and protons.^[^ ^61^ ^]^ In this work, we reduced nitrobenzene to aniline **6a** with 100% selectivity and 7% yield (under non‐optimized conditions) with pre‐synthesized K‐PHI(e^‒^/H^+^) in dark (Figure 4B).

Collectively, these examples illustrate remarkable reduction power of K‐PHI(e^‒^/H^+^) to drive uphill processes via MS‐PCET. Overall, its negatively charged microporous structure is the most crucial parameter that explains high performance of K‐PHI nanoparticles in the MS‐PCET reactions. It gives degenerate semiconductor with higher degree of loading with electrons and charge‐compensating H^+^, 1 mmol g^−1^ or ca. 10^21^ cm^−3^ (determined by titration of K‐PHI(e^‒^/H^+^) with methylviologen),^[^ ^37^ ^]^ compared to more dense covalent mpg‐CN (ca. 6·10^19^ cm^−3^),^[^ ^37^ ^]^ which correlates with the yield of **2a** (Table S15). Although average diameter of K‐PHI particles is ca. 100 nm (Figure S1J),^[^ ^30^ ^]^ which is 10 times larger compared to that of metal oxides, the degree of doping in K‐PHI(e^‒^/H^+^) is comparable to the latter. Concentration of e^‒^/H^+^ in TiO_2_ was reported to be 3‒5·10^21^ cm^−3^ (ref. [21]) and ca. 2·10^19^‒4·10^20^ cm^−3^ in ZnO.^[^ ^22^, ^62^ ^]^ Considering that up‐shift of the Fermi level in semiconductors is proportional to the concentration of charge carriers (n), *E*_F_‒*E*_C_ ∼ n^2/3^,^[^ ^20^ ^]^ higher doping degree affords material with stronger reduction power. Upon partial discharging, the Fermi level shifts downward and reduction power of the SCNP(e^‒^/H^+^) decreases. Under the optimized conditions with respect to the yield of **2a** (100%), by taking into account loading of K‐PHI (80 mg), 4‐bromoacetophenone (0.05 mmol), and maximum amount of stored e^‒^/H^+^ (1 mmol g^−1^), the efficiency of e^‒^/H^+^ transfer in the dark phase to the aryl halide is 63%, which is explained by decline of the reduction power of K‐PHI(e^‒^/H^+^) once the SCNP is partially discharged in dark. MS‐PCET from K‐PHI under continuous light irradiation requires loading of the material only in catalytic quantities as the K‐PHI(e^‒^/H^+^) nanoparticles are constantly recharged at the expense of a sacrificial agent.

## 4 DISCUSSION

Ionic carbon nitrides, in this work represented by K‐PHI nanoparticles, direct energy of electromagnetic radiation to drive uphill processes via MS‐PCET with electrons stored in the π conjugated structure and charge compensating protons, which are stored in the micropores. Based on thermochemical calculation, the binding energy of e^‒^/H^+^ pairs in charged K‐PHI nanoparticles is < 5.2 kcal mol^−1^, which makes the material a very potent reductant. Using K‐PHI nanoparticles charged electrons and protons under light irradiation, 14 different aryl halides have been reduced with 7–100% yield of the corresponding aromatic compounds in dark. Scope of the methodology has been expanded to the 6e^‒^/6H^+^ reduction of nitrobenzene to aniline and the generation of ketyl radical from the chalcone followed by dimerization to the dienone in dark. The integration of several features that up to now were intrinsic for plants and natural photosynthesis into a nanomaterial composed of abundant C,N,K elements offers a powerful tool for synthetic organic chemistry. The concept developed herein has high potential for “storage” of natural sunlight and subsequent employment in synthetically useful uphill processes in the flask.

## CONFLICT OF INTEREST

A patent WO/2019/081036 has been filed by Max Planck Gesellschaft zur Förderung der Wissenschaften E.V. in which Aleksandr Savateev and Markus Antonietti are listed as co‐authors.

## AUTHOR CONTRIBUTIONS

Stefano Mazzanti contributed to perform catalytic experiments and materials synthesis, preparation of the manuscript, and the ESI. Clara Schritt and Katharina ten Brummelhuis contributed to perform catalytic experiments and materials synthesis. Aleksandr Savateev conceived the idea and contributed in the preparation of the manuscript and ESI. Markus Antonietti contributed in the planning of the research work.

## Supporting information



Supporting Information
