# Supplementary material for: Multisite PCET with photocharged carbon nitride in dark
Source: Exploration (Beijing). 2021 Dec 16;1(3):20210063. doi: 10.1002/EXP.20210063 (PMC10190955; doi:10.1002/EXP.20210063)
Supplement: Supplementary file 1 — Supporting Information [file EXP2-1-20210063-s001.pdf]

Supporting information

Multisite PCET with Photocharged Carbon Nitride in Dark

Stefano Mazzanti[1], Clara Schritt[1,2], Katharina ten Brummelhuis[1],
Markus Antonietti[1], Aleksandr Savateev[1*]

1 Max-Planck Institute of Colloids and Interfaces, Department of Colloid Chemistry, Research Campus Golm, Am Mühlenberg 1, 14476 Potsdam, Germany.

2 Freie Universität Berlin, Kaiserswerther Str. 16-18, 14195 Berlin, Germany

* Corresponding author. E-mail address: oleksandr.savatieiev@mpikg.mpg.de (A. Savateev).

Materials

TiO_2 was purchased from Sigma-Aldrich (CAS number 1317-80-2, rutile, nanopowder 10x40 nm, specific surface area 50 m^2g^{-1}).

1. Supplementary methods

Powder X-Ray diffraction patterns were measured on a Bruker D8 Advance diffractometer equipped with a scintillation counter detector with CuKα radiation (λ = 0.15418 nm) applying 2θ step size of 0.05° and counting time of 3s per step.

Nitrogen adsorption/desorption measurements were performed after degassing the samples at 150 °C for 20 hours using a Quantachrome Quadrasorb SI-MP porosimeter at 77.4 K. The specific surface areas were calculated by applying the Brunauer-Emmett-Teller (BET) model to adsorption isotherms for $0.05 < p/p_0 < 0.3$ using the QuadraWin 5.11 software package.

^{1}H and ^{13}C NMR spectra were recorded on Agilent 400 MHz (at 400 MHz for Protons and 101 MHz for Carbon-13). Chemical shifts are reported in ppm versus solvent residual peaks: *DMSO-d$_6$* 2.50 ppm (^{1}H NMR), 39.52 ppm (^{13}C NMR)

Mass spectral data were obtained using Agilent GC 6890 gas chromatograph, equipped with HP-5MS column (inner diameter=0.25 mm, length=30 m, and film=0.25 μm), coupled with Agilent MSD 5975 mass spectrometer (electron ionization).

The X-ray photoelectron spectroscopy (XPS) measurements were carried out in an ultrahigh vacuum (UHV) spectrometer equipped with a VSW Class WA hemispherical electron analyzer. A dual anode Al Kα X-ray source (1486.6 eV) was used as incident radiation. Survey and high resolution spectra were recorded in constant pass energy mode (44 and 22 eV, respectively). During the UPS (He I excitation energy $h\nu$=21.23 eV) measurements a bias of 15.32 V was applied to the sample, in order to avoid interference of the spectrometer threshold in the UP spectra. The values of the valence band maximum (VBM) are determined by fitting a straight line into the leading edge.

Optical absorbance spectra of powders were measured on a Shimadzu UV 2600 equipped with an integrating sphere.

Emission spectra were recorded on Jasco FP-8300 instrument. The excitation wavelength was 360 nm.

Energy dispersive X-Ray (EDX) analysis was performed on JEOL JSM-7500F electron microscope equipped with two Oxford Instruments EDX detectors, located at opposite sides from the sample. The angle between the sample film surface and EDX detector axis was 28°.

The TEM measurements were acquired using a double-corrected Jeol ARM200F, equipped with a cold field emission gun and a Gatan GIF Quantum. The used acceleration voltage was 200kV and the emission was set to 10μA in order to reduce beam damage. An objective aperture with a diameter of 60μm was introduced into the beam to improve the contrast while still allowing for atomic resolution.

Hydrodynamic diameter of K-PHI particles in water was measured using Malvern Zetasizer instrument.

Cyclic voltammetry (CV) measurements were performed in a glass single-compartment electrochemical cell. Glassy carbon (diameter 3 mm) was used as a working electrode (WE), Ag wire in $AgNO_3$ (0.01M) as a reference electrode (RE), Pt wire as a counter electrode. Each compound was studied in a 30 mM concentration in a 0.1 M tetrabutylammonium perchlorate (TBAP) and DMSO electrolyte solution (10 mL). Before voltammograms were recorded, the solution was purged with Ar, and an Ar flow was kept in the headspace volume

of the electrochemical cell during CV measurements. A potential scan rate of 0.050 V s^{-1} was chosen, and the potential window ranging from +1.5 V to −2.5 V (and backwards) was investigated. Cyclic voltammetry was performed under room-temperature conditions (~20-22 °C). Values have been converted using vs SCE using ferrocene as internal standard.

Irradiance of the LED modules was measured using PM400 Optical Power and Energy Meter equipped with the integrating sphere S142C and purchased from Thorlabs.

Carbon nitride photocatalysts synthesis and characterization

K-PHI

Potassium poly(heptazine imide) (K-PHI) was synthesized according to the previously described procedure.[2] Mixture of lithium chloride (3.71 g), potassium chloride (4.54 g) and 5-aminotetrazole (1.65 g) was ground in ball mill for 5 min at the shaking rate 25 s^{-1}. Reaction mixtures were transferred into porcelain crucibles and covered with lids. Crucibles were placed in the oven and heated under constant nitrogen flow (15 L·min^{-1}) and atmospheric pressure at a following temperature regime: heating from room temperature to 600 °C for 4 hours, annealing at 600 °C for 4 hours. After completion of the heating program, the crucibles were allowed to cool slowly to room temperature under nitrogen flow. The crude products were removed from the crucibles, washed with deionized water (100 mL) for 3 hours in order to remove salts, then filtered, extensively washed with deionized water and dried in a vacuum oven (20 mbar) at 50 °C for 15 h.

mpg-CN

Cyanamide (3.0 g) and Ludox HS-40 (7.5 g) were mixed in a 10 mL glass vial. The mixture was stirred at room temperature for 30 min until cyanamide has completely dissolved. The resultant solution was stirred at +60°C for 16 h until water has completely evaporated. Magnetic stir bar was removed and white solid was transferred to the porcelain crucible and heated under N_2 flow in the oven. The temperature was increased from room temperature to 550°C within 4 h and maintained at 550°C for 4 h. The crucible was spontaneously cooled to room temperature. The solid from the crucible was briefly grinded in the mortar and transferred to the polypropylene bottle. A solution of $(NH_4)HF_2$ (0.24 g·mL^{-1}, 50 mL) was added and suspension was stirred at room temperature for 24 h. The solid was filtered,

thoroughly washed with water, once with ethanol and dried in vacuum (55°C, 20 mbar) overnight.

g-C_3N_4

Diacyandiamide (15.0 g) was calcined at 600°C for 4 h under flow of nitrogen (15 L min^{-1}) in a porcelain crucible. Yellow solid was ground in mortar.

Standard procedure for photocatalytic dehalogenation in dark conditions

In a 8 mL vial with stirrer (3 cm length, vertically placed), 80 mg of K-PHI, 4 mL of DMSO-d_6 and 560 µL of DIPEA were added. The vial was closed with a rubber cap. The solution was degassed for 2 minutes via double needle technique, fluxing nitrogen. The reaction vessel was irradiated (light conditions) with a 50 W Blue light LED module at 1 cm distance (0.37 W·cm^{-2}), cooled with a fan for 24 hours (T_1, τ_1). Afterwards, 1 mL DMSO-d_6 solution (degassed as described above) containing 0.05 mmol of aryl halide (**1a**-**1t**) was added. Then, the vial was placed in an oil bath (1 cm deep) at 80 °C for 20 hours (T_2, τ_2), wrapped in aluminum foil, without any light irradiation (dark conditions). K-PHI was separated by filtration through 200 nm syringe filter followed by addition of 1,3,5,-trimethoxybenzene as internal standard. The yield was determined via ^{1}H NMR. See Figure S5 for schematic overview.

Standard procedure for photocatalytic generation of ketyl radical in dark conditions

In a 8 mL vial with stirrer (3 cm length, vertically placed), 80 mg of K-PHI, 4 mL of DMSO and 560 µL of DIPEA were added. The vial was closed with a rubber cap. The solution was degassed for 2 minutes via double needle technique, fluxing nitrogen. The reaction vessel was irradiated (light conditions) with a 50 W Blue light LED module at 1 cm distance (0.37 W·cm^{-2}), cooled with a fan for 24 hours (T_1, τ_1). Afterwards, 1 mL DMSO solution (degassed as above) containing chalcone **3a** (0.05 mmol, 10.4 mg) was added. Then, the vial was placed in an oil bath (1 cm deep) at 80 °C for 20 hours (T_2, τ_2), wrapped in aluminum foil, without any light irradiation (dark conditions). K-PHI was separated by filtration through 200 nm syringe filter followed by analysis via GC-MS to determine the yield.

Standard procedure for photocatalytic reduction of nitrobenzene in dark conditions

In a 8 mL vial with stirrer (3 cm length, vertically placed), 80 mg of K-PHI, 4 mL of DMSO and 560 µL of DIPEA were added. The vial was closed with a rubber cap. The solution was degassed for 2 minutes via double needle technique, fluxing nitrogen. The reaction vessel was irradiated (light conditions) with a 50 W Blue light LED module at 1 cm distance (0.37 W·cm^{-2}), cooled with a fan for 24 hours (T_1, τ_1). Afterwards, 1 mL DMSO solution (degassed as above) containing nitrobenzene **5a** (0.05 mmol, 5.13 µL) was added. Then, the

vial was placed in an oil bath (1 cm deep) at 80 °C for 20 hours (T_2, τ_2), wrapped in aluminum foil, without any light irradiation (dark conditions). K-PHI was separated by filtration through 200 nm syringe filter followed by analysis via GC-MS to determine the yield.

2. Supplementary tables

Entry	Semiconductor	Amine	DMSO (mL)	τ_1 (h)	τ_2 (h)	T_1 (°C)	T_2 (°C)	Yield (%)
**1**	K-PHI (20 mg)	TEA (350 µL)	1	25	20	RT	50	6
**2**	mpg-CN (20 mg)	TEA (350 µL)	1	25	20	RT	50	Traces
**3**	g-CN (20 mg)	TEA (350 µL)	1	25	20	RT	50	Traces
**4**	Na-PHI (20 mg)	TEA (350 µL)	1	25	20	RT	50	Traces
**5**	K-PHI (40 mg)	TEA (350 µL)	1	25	20	RT	50	15
**6**	K-PHI (80 mg)	TEA (350 µL)	1	25	20	RT	50	22
**7**	K-PHI (20 mg)	TEA (350 µL)	1	16	5	RT	80	14

Table S1 – Screening of semiconductors. Reactions were performed following the standard procedure. Parameters deviated from STD conditions are reported. Samples have been analyzed via GC-MS to determine the yield.

Entry	Substrate	K-PHI	Amine	DMSO	τ_1 (h)	τ_2 (h)	Yield (%)
**1**	0.025 mmol	20 mg	TEA (3.5 µL)	1 mL	23	19	Traces
**2**	0.025 mmol	20 mg	TEA (7 µL)	1 mL	23	19	12
**3**	0.025 mmol	20 mg	TEA (14 µL)	1 mL	23	19	24
**4**	0.025 mmol	20 mg	TEA (28 µL)	1 mL	23	19	25
**5**	0.025 mmol	20 mg	TEA (112 µL)	1 mL	23	18	31
**6**	0.025 mmol	20 mg	TEA (168 µL)	1 mL	23	18	28
**7**	0.025 mmol	20 mg	THIQ (102 µL)	1 mL	26	17	16
**8**	0.025 mmol	20 mg	TEOA (106 µL)	1 mL	26	17	25
**9**	0.025 mmol	20 mg	DIPEA (106 µL)	1 mL	26	17	40
**10**	0.025 mmol	20 mg	1-phenylpyrrolidine (118 (µL)	1 mL	26	17	7

Table S2 – Amines screening. Reactions were performed following the standard procedure. Parameters deviated from STD conditions are reported. Samples have been analyzed via GC-MS to determine the yield.

Entry	Substrate	DIPEA	Light distance	DMSO	K-PHI	τ1 (h)	τ2 (h)	Yield (%)
**1**	0.025 mmol	140 µL	1 cm	1 mL	20 mg	22	19	67
**2**	0.025 mmol	140 µL	1 cm	1 mL	80 mg	22	19	31
**3**	0.025 mmol	140 µL	1 cm	3 mL	80 mg	22	20	71

Table S3 – Studying the effect of dilution and K-PHI loading. Reactions were performed following the standard procedure. Parameters deviated from STD conditions are reported. Samples have been analyzed via GC-MS to determine the yield.

Entry	DIPEA	DMSO	K-PHI	Light (at 1 cm)	τ1 (h)	τ2 (h)	Yield (%)
**1**	140 µL	1 mL	20 mg	Blue (465 nm)	20	5	34
**2**	140 µL	1 mL	20 mg	Purple (410 nm)	20	5	11
**3**	140 µL	1 mL	20 mg	UV (365 nm)	20	5	52

Table S4 – Studying the effect of photon energy. Reactions were performed following the standard procedure. Parameters deviated from STD conditions are reported. Samples have been analyzed via GC-MS to determine the yield. Lower yield in case of using purple light is apparently due to higher optical power of the LED, which results in higher temperature of the reaction mixture. As a result, lower amount of electrons and protons stored in K-PHI nanoparticles and accessible on the second step in the dark.

Entry	Substrate	K-PHI	DIPEA (µL)	DMSO	τ1 (h)	τ1 (h)	Yield (%)
**1**	0.025 mmol	40 mg	140	2 mL	23	22	63
**2**	0.025 mmol	40 mg	180	2 mL	23	22	51
**3**	0.025 mmol	40 mg	100	2 mL	23	22	69
**4**	0.025 mmol	40 mg	280	2 mL	23	22	100

Table S5 – Screening the amount of DIPEA. Reactions were performed following the standard procedure. Parameters deviated from STD conditions are reported. Samples have been analyzed via GC-MS to determine the yield.

Entry	τ1 (h)	τ2 (h)	Yield (%)
**1**	8	24	80
**2**	20	24	100
**3**	24	24	100
**4**	24	8	70
**5**	24	20	100
**6**	24	20	100

Table S6 – Reaction time screening. Reactions were performed following the standard procedure. Parameters deviated from STD conditions are reported. Samples have been analyzed via GC-MS to determine the yield.

Entry	Catalyst	Light	T_2 (°C)	Yield (%)
**1**	K-PHI (80 mg)	Blue	80	100%
**2**	K-PHI (80 mg)	Blue	**RT**	7%
**3**	K-PHI (80 mg)	None	80	Traces
**4**	None	Blue	80	Traces
**5**	Na-PHI (80 mg)	Blue	80	61
**6**	g-CN (80 mg)	Blue	80	Traces
**7**	mpg-CN (80 mg)	Blue	80	12
**8**	TiO_2 (12.8 mg)	UV	80	Traces
**9**[a]	K-PHI (80 mg)	Blue	/	100
**10**	K-PHI (80 mg)[b]	Blue	80	49%

Table S7 – Reactions were performed following the standard procedure. Parameters deviated from STD conditions are reported. a) Reaction performed with the substrate added before irradiation. Reaction was stopped after the light phase. b) Recycled catalyst. Samples have been analyzed via GC-MS to determine the yield.

Entry	Solvent	Co-solvent	Yield (%)
**1**	DMSO	/	100
**2**	CH_3CN	/	0
**3**	CH_3CN	80 µL H_2O	0
**4**	DMF	/	7
**5**	Toluene	/	0
**6**	DMSO	/	100
**7**	DMSO[a]	20 µL H_2O	90
**8**	DMSO[a]	40 µL H_2O	97
**9**	DMSO[a]	80 µL H_2O	100

Table S8 – Reactions were performed following the standard procedure. Parameters deviated from STD conditions are reported. a) Using a new bottle of dry DMSO. Samples have been analyzed via GC-MS to determine the yield.

Entry	Storage after 24 h irradiation	Yield (%)
**1**	3 days	75
**2**	7 days	10
**3**	7 days[a]	51

Table S9 – Reactions were performed following the standard procedure. Parameters deviated from STD conditions are reported. a) Kept in freezer at -25 °C Samples have been analyzed via GC-MS to determine the yield.

Entry	Substrate	$E_{p1/2}$ (vs SCE)	Reference	BDE, kcal mol^{-1}	Reference
1	**1a**	−1.84 V	This work	80.4	[1]
2	**1c**	–	–	70.3	[2]
3	**1d**	−1.70 V	[3]	–	–
4	**1e**	−1.90 V	[3]	95.7	[1]
5	**1f**	−1.85 V	[4]	–	–
6	**1h**	–	–	95.0	[1]
7	**1l**	–	–	65.6	[1]
8	**1n**	−1.95 V	[4]	–	–
9	**1o**	−1.95 V	This work	70.6	[2]
10	**1p**	−1.82 V	[5]	–	–

Table S10 – Half peak reduction potentials (Figure S5) and BDE values of C–I, C–Br and C–Cl bonds in aryl halides

3. Supplementary figures

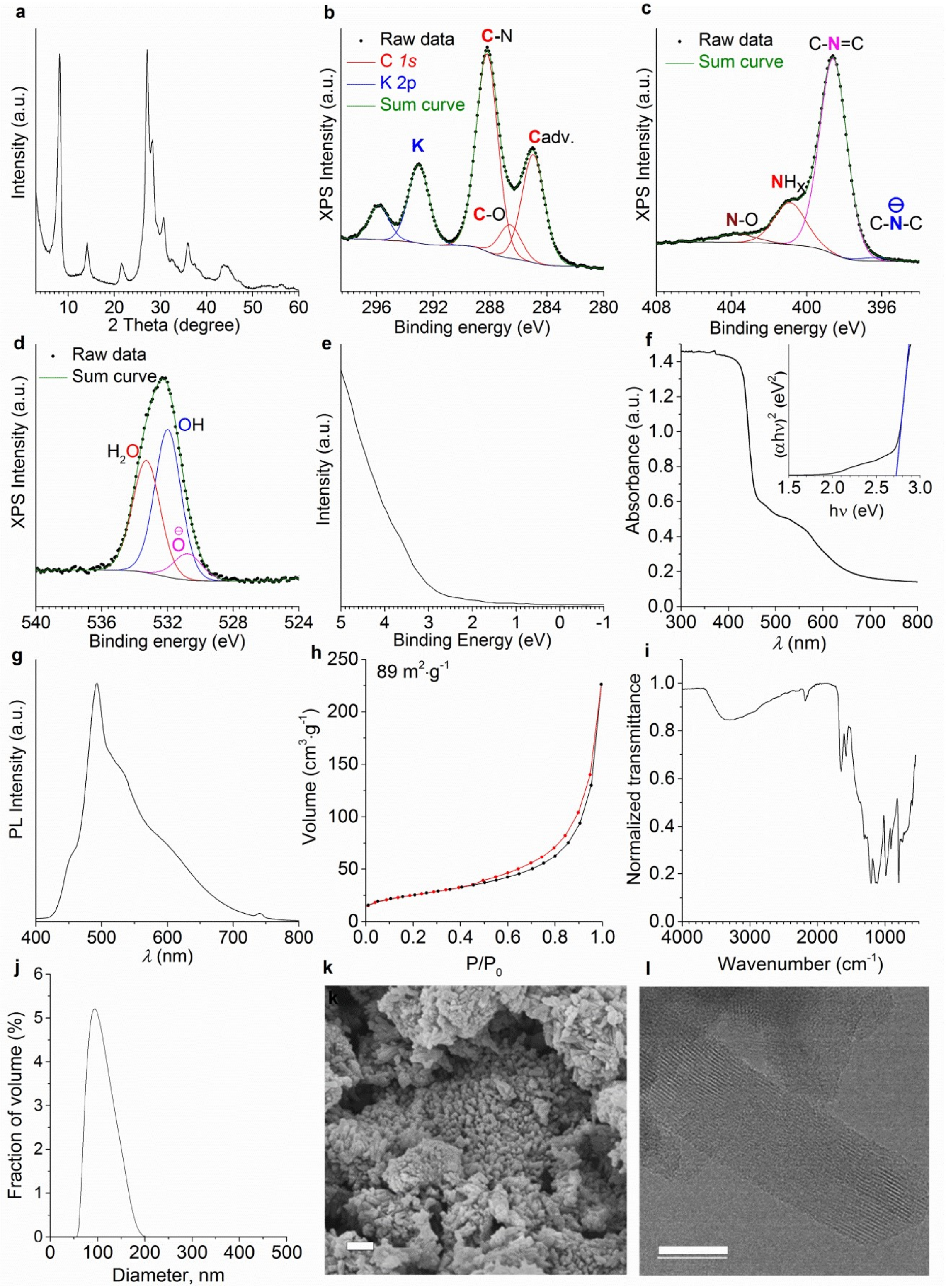

Figure S 1 - K-PHI characterization. (A) PXRD pattern of K-PHI; (B) XPS C 1s and K 2p spectra of K-PHI; (C) XPS N 1s spectrum of K-PHI; (D) XPS O 1s spectrum of K-PHI; (E) UPS spectrum of K-PHI; (F) UV-vis absorption spectrum of K-PHI with Tauc plot as inset assuming that K-PHI is a direct semiconductor; (G) room temperature PL spectrum of K-PHI obtained upon excitation

with 350 nm wavelength; (H) N_2 sorption isotherm measured at 77 K. BET surface area; (I) FT-IR spectrum of K-PHI; (J) DLS analysis of K-PHI suspension in water; (K) representative SEM image of K-PHI photocatalyst. Scale bar 200 nm; (L) AC-HRTEM image of K-PHI photocatalyst. Scale bar 20nm. Reproduced with permission from reference *[6].*

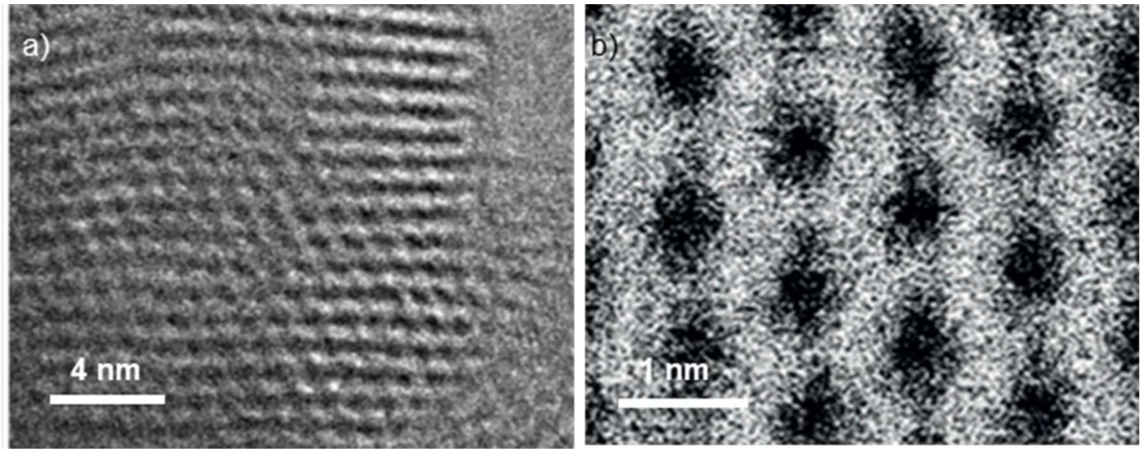

Figure S 2 – AC-HRTEM images of K-PHI microporous structure. (A) Reproduced with permission from reference [7]. (B) Reproduced from reference [8].

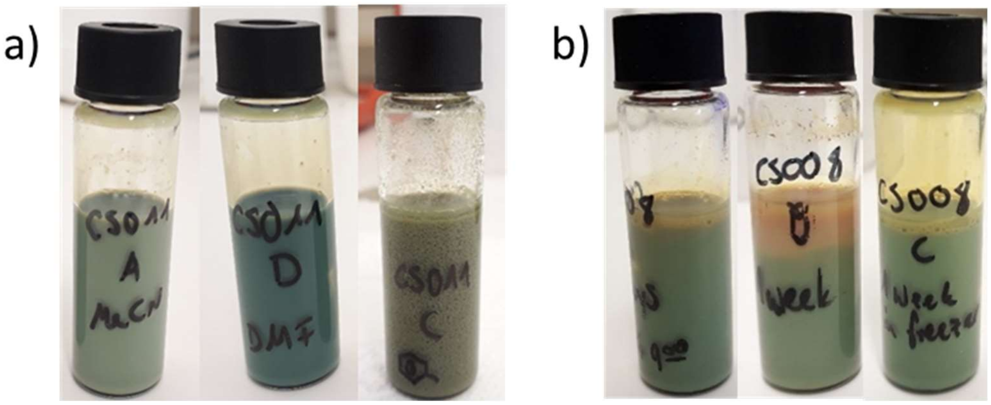

Figure S 3 – (A) Samples after irradiation in different solvent: CH_3CN, DMF and toluene, respectively from left to right. (B) Samples after irradiation stored for 3 days, 7 days and 7 days in freezer, respectively from left to right.

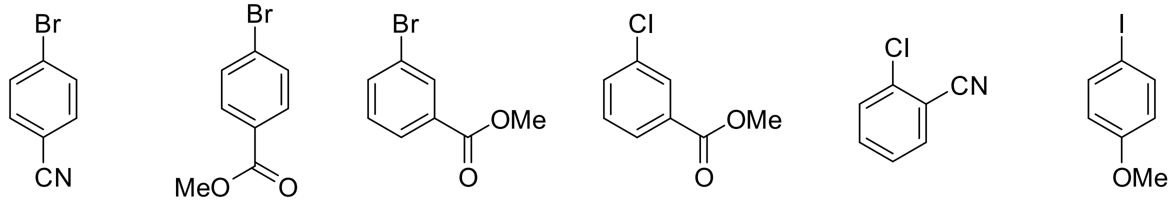

1o, n.d. **1p**, n.d. **1q**, n.d. **1r**, n.d. **1s**, traces **1t**, n.d.

Figure S 4 – Structure of aryl halides that did not react under the standard conditions. Samples have been analyzed via GC-MS to determine the yield. n.d. – the product of aryl halide reduction was not detected in the GC-MS chromatograms.

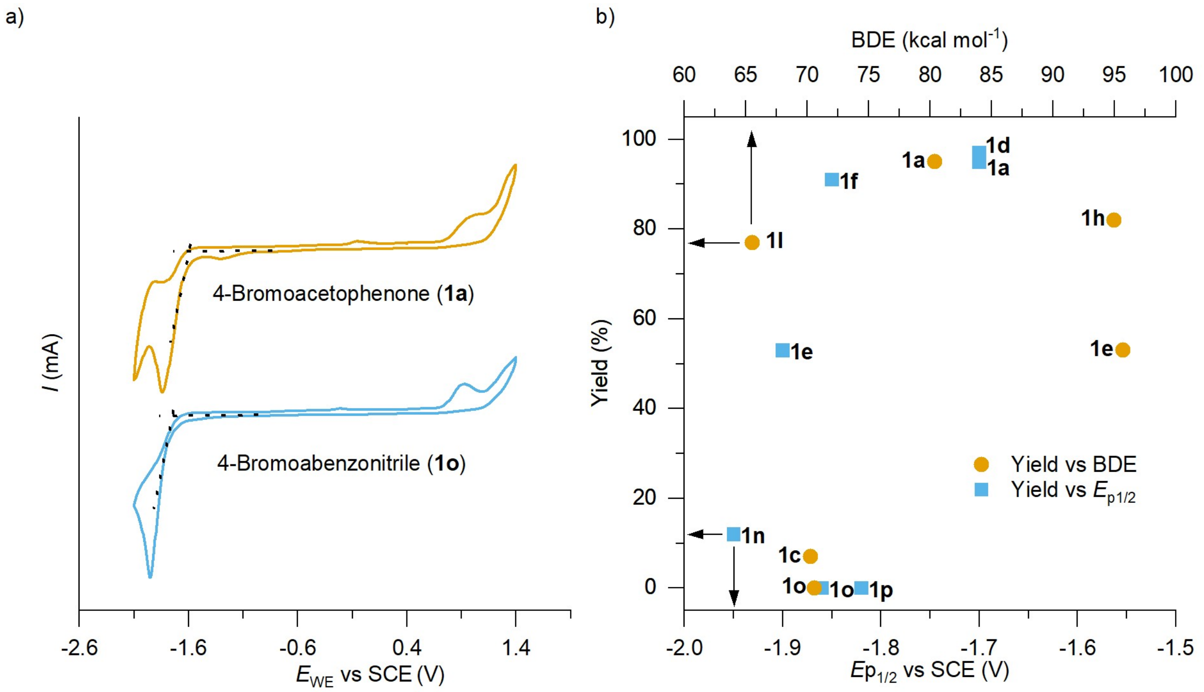

*Figure S 5 – (A) Cyclic voltammetry of **1a** and **1o**. (B) Correlation of the yield of aromatic compound with the half-peak potentials ($E_{p1/2}$) and BDE values of the corresponding substrates reported in Table S10.*

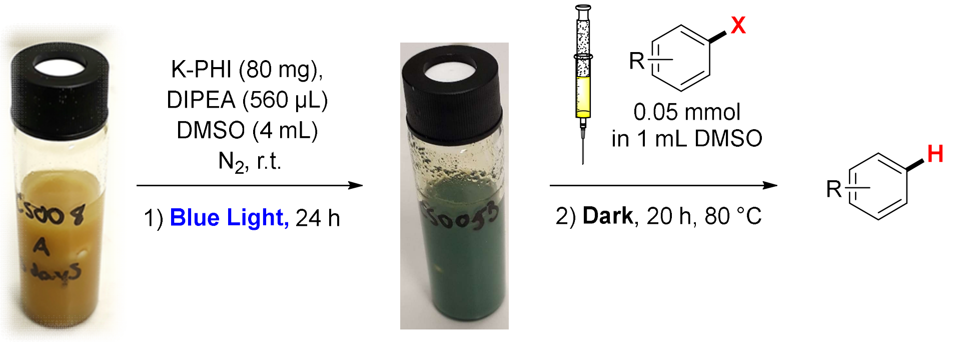

Figure S 6 – General procedure of dark photocatalytic aryl halide dehalogenation.

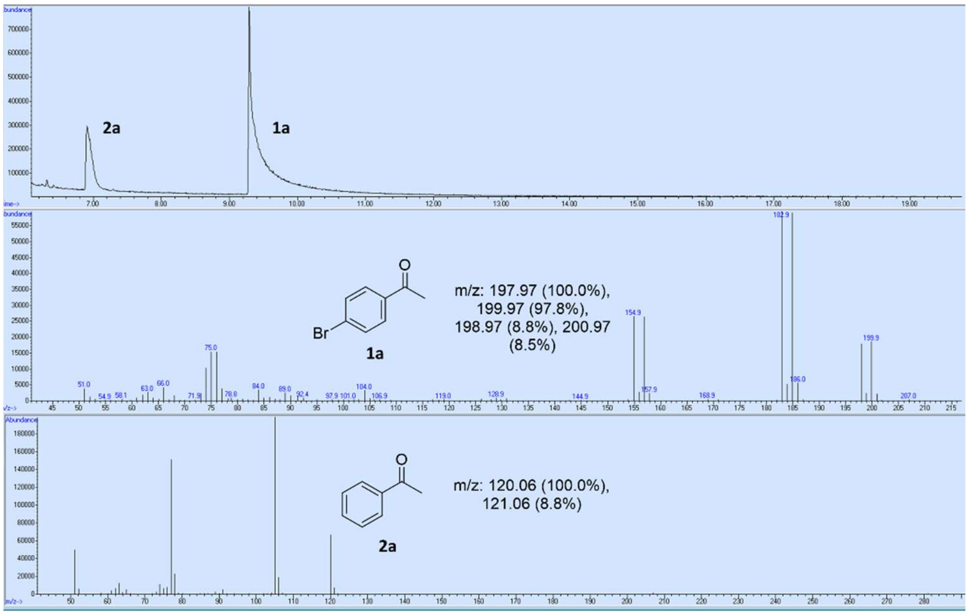

*Figure S 7 – Example of GC-MS chromatogram and spectra during optimization studies of dehalogenation of **1a.***

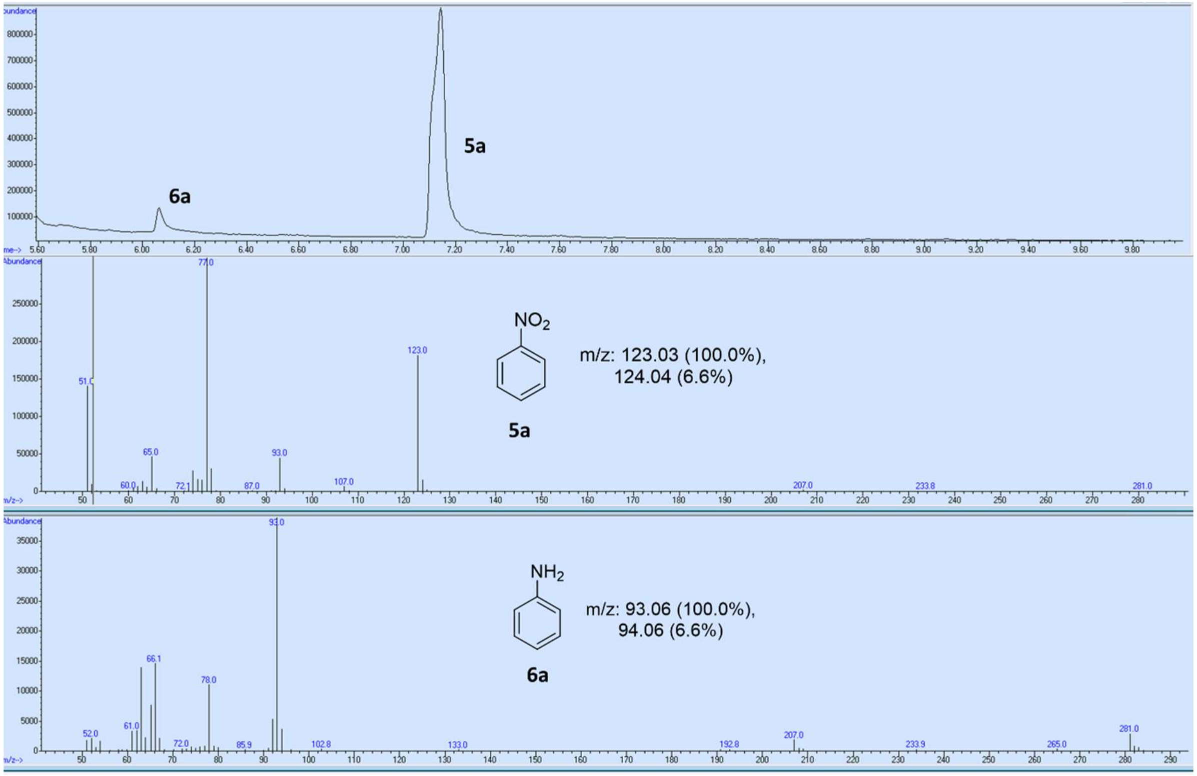

*Figure S 8 – GC-MS chromatogram and spectra of nitrobenzene **5a** reduction.*

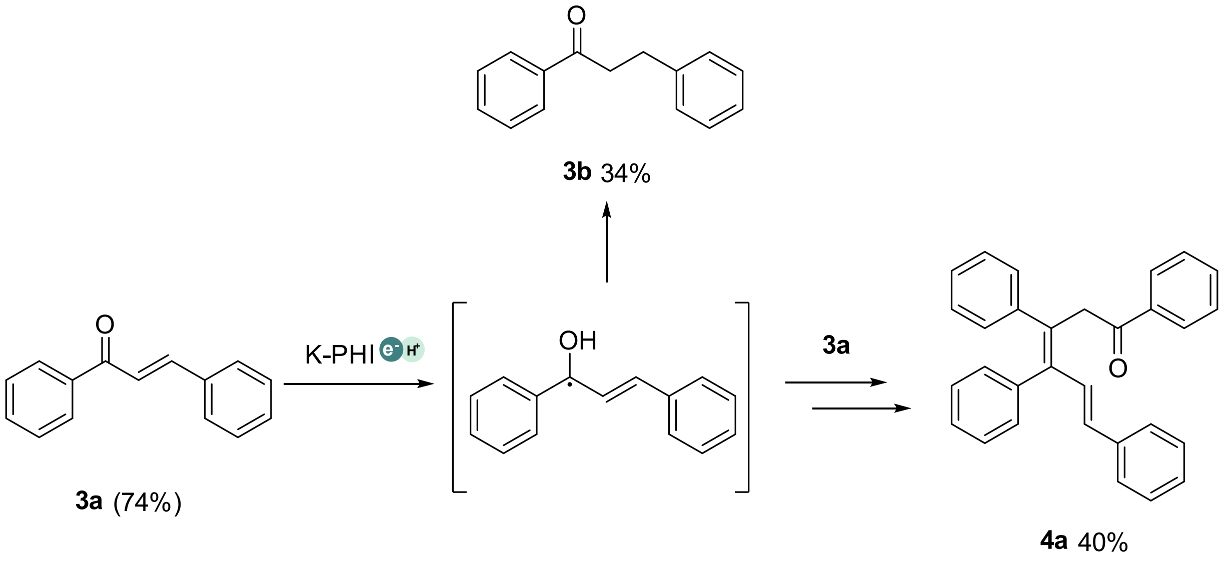

*Figure S 9 – Proposed mechanism for chalcone **3a** reduction, ketyl radical formation and dimerization. Chalcone conversion reported in parenthesis.*

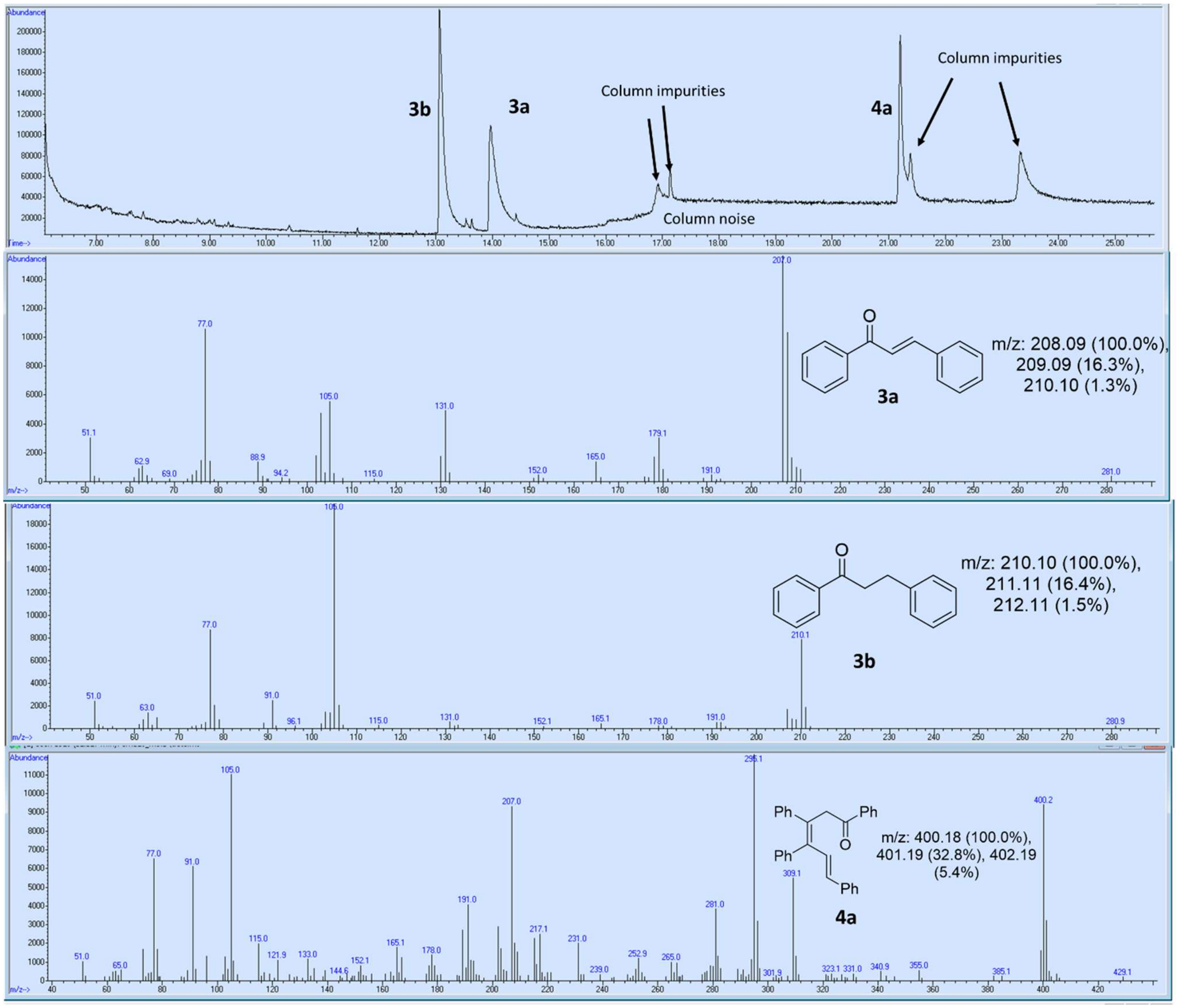

*Figure S 10 – GC-MS chromatogram and spectra of chalcone **3a** reduction.*

Thermochemical calculations

1. Reduction of phenyl radical from bromobenzene via MS-PCET by e^-/H^+.

Reduction of bromobenzene to phenyl radical via MS-PCET by e^-/H^+ can be described by the equation (1):

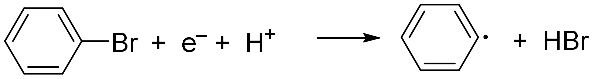

ΔG_1 (1)

which is a combination of equations (2) and (3):

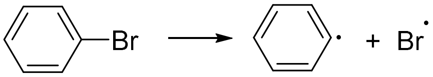

ΔG_2 (2)

$$HBr = Br^{\bullet} + H^{\bullet} \qquad \Delta G_3 \qquad (3)$$

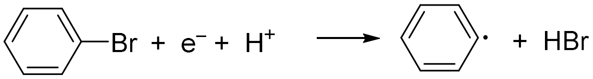

$\Delta G_1 = \Delta G_2 - \Delta G_3$

Table S11. Reported and estimated BDFE values.

	**Reaction**	**ΔG or BDFE, kcal mol^{-1}**	**Comment**
(2)	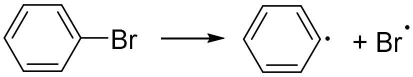	$\Delta G_2 = 74.6$	Gas phase, 298 K. Calculated from the equation $\Delta G_2 = BDE - T\Delta S$ (BDE = 82.3 kcal mol^{-1} from the reference [[9]] (computed value), ΔS = 0.026 kcal mol^{-1} from the reference [[10]]).
(3)	$HBr = Br^{\bullet} + H^{\bullet}$	$\Delta G_3 = 79.8$	Gas phase, 298 K. Calculated from the equation $\Delta G_2 = BDE - T\Delta S$ (BDE = 87.54 kcal mol^{-1} from the reference [[10]]

			(experimental value), ΔS = 0.026 kcal mol^{-1} from the reference [[10]]).
(1)	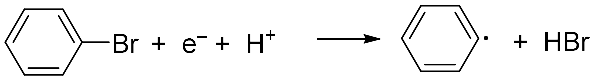	$\Delta G_3 = -5.2$	Gas, 298K. Calculated based on values of ΔG_2 and ΔG_3.

2. Reduction of O_2 to H_2O_2 by e^-/H^+ stored in the semiconductor.[11],[12]

Reduction of O_2 to H_2O_2 by e^-/H^+ stored in the semiconductor nanoparticle (SCNP) can be described by the equation (1):

$$2SCNP(e^-/H^+) + O_2 = 2SCNP + H_2O_2 \qquad \Delta G_1 \qquad (1)$$

which is a combination of equations (2), (3) and (4):

$$HO_2^\bullet = O_2 + H^\bullet \qquad \Delta G_2 \qquad (2)$$

$$H_2O_2 = HO_2^\bullet + H^\bullet \qquad \Delta G_3 \qquad (3)$$

$$SCNP(e^-/H^+) = SCNP + H^\bullet \qquad \Delta G_4 \qquad (4)$$

$$2SCNP(e^-/H^+) + O_2 = 2SCNP + H_2O_2 \qquad \Delta G_1 = 2\cdot\Delta G_4 - \Delta G_2 - \Delta G_3$$

ΔG_2, ΔG_3 and ΔG_4 – are the BDFE of the corresponding compounds in kcal mol^{-1}.

The condition for reduction of O_2 to H_2O_2 by e^-/H^+ stored in the SCNP is that $\Delta G_1 < 0$ (spontaneous process). The upper limit of ΔG_4 can be estimated from the relation:

$$\Delta G_4 < \frac{1}{2}(\Delta G_2 + \Delta G_3)$$

The results for different conditions (gas or water) are summarized in the Table S12.

Table S12. Reported and estimated BDFE values.

	**Equation**	**BDFE, kcal mol^{-1}**	**Comment**
(2)	$HO_2^\bullet = O_2 + H^\bullet$		
		$\Delta G_2 = 42.7$	Gas, 298K. Data from reference [[13]].

		ΔG_2 = ~58	DMSO, 298 K. Data from reference [[13]].
		ΔG_2 = 60.6	H_2O, 298 K. Data from reference [[13]].
(3)	$H_2O_2 = HO_2^{\bullet} + H^{\bullet}$		
		ΔG_3 = 79.6	Gas, 298K. Data from reference [[13]].
		ΔG_3 =91.0	H_2O, 298 K. Data from reference [[13]].
(4)	SCNP(e^-/H^+) = SCNP + $H^{\bullet}$		
		ΔG_4 < 61.2	Gas, 298K. Estimated based on values of ΔG_2 and ΔG_3.
		ΔG_4 < 75.8	H_2O, 298 K. Estimated based on values of ΔG_2 and ΔG_3.

3. Reduction of ketone to ketyl radical by e^-/H^+ stored in K-PHI.[14]

Reduction of ketone to ketyl radical by e^-/H^+ stored in K-PHI can be described by the equation (1):

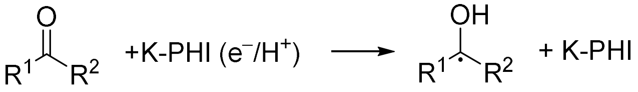

ΔG_1 (1)

which is a combination of equations (2) and (3):

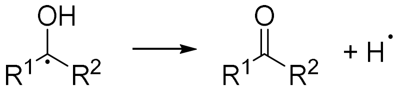

ΔG_2 (2)

K-PHI(e^-/H^+) = K-PHI + $H^{\bullet}$ ΔG_3 (3)

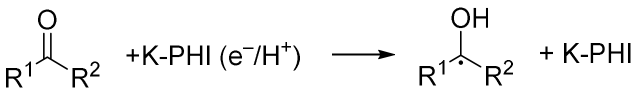

$\Delta G_1 = \Delta G_3 - \Delta G_2$

ΔG_2, ΔG_3 – are the BDFE of the corresponding compounds in kcal mol^{-1}.

The condition for reduction of ketone to its ketyl radical by e^-/H^+ stored in K-PHI is that ΔG_1 < 0 (spontaneous reaction). The upper limit of ΔG_3 can be estimated from the relation:

$$\Delta G_3 < \Delta G_2$$

The results are summarized in the Table S13.

Table S13. Reported and estimated BDFE values.

	Equation	BDFE, kcal mol^{-1}	Comment
(2)	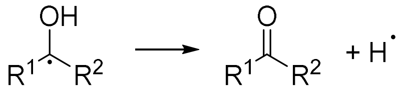	$\Delta G_2 = 25.69$	Calculated value for acetophenone ketyl radical. Data from reference [[15]].
(3)	K-PHI (e^-/H^+) = K-PHI + $H^\bullet$	$\Delta G_3 < 25.69$	Estimated based on the value of ΔG_2.

4. Generation of phenyl radical from bromobenzene using e^-/H^+ stored in K-PHI via MS-PCET (this work).

Reduction of bromobenzene to phenyl radical by e^-/H^+ stored in K-PHI can be described by the equation (1):

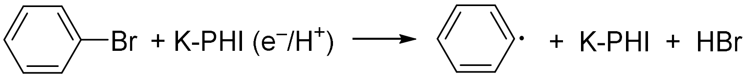 ΔG_1 (1)

which is a combination of equations (2), (3) and (4):

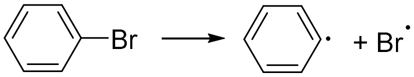

ΔG_2 (2)

$HBr = Br^\bullet + H^\bullet$ ΔG_3 (3)

K-PHI(e^-/H^+) = K-PHI + $H^\bullet$ ΔG_4 (4)

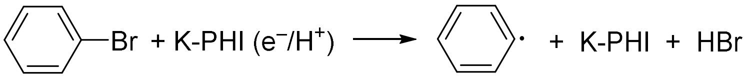 $\Delta G_1 = \Delta G_2 + \Delta G_4 - \Delta G_3$

ΔG_2, ΔG_3, ΔG_4 – are the BDFE of the corresponding compounds in kcal mol^{-1}.

The condition for reduction of bromobenzene to phenyl radical by e^-/H^+ stored in K-PHI is that $\Delta G_1 < 0$ (spontaneous reaction). Therefore, the upper limit of ΔG_4 can be estimated from the relation:

$$\Delta G_4 < \Delta G_3 - \Delta G_2$$

The results for different conditions (gas or water) are summarized in the Table S14.

Table S14. Reported and estimated BDFE values.

	**Reaction**	**BDFE, kcal mol^{-1}**	**Comment**
(2)	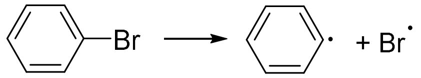	$\Delta G_2 = 74.6$	Gas phase, 298 K. Calculated from the equation ΔG_2 = BDE – TΔS (BDE = 82.3 kcal mol^{-1} from the reference [[9]] (computed value), ΔS = 0.026 kcal mol^{-1} from the reference [[10]]).
		$\Delta G_2 = 73.2$	H_2O, 298K. Computed value. Data from the reference [[9]].
(3)	HBr = Br• + H•	$\Delta G_3 = 79.8$	Gas phase, 298 K. Calculated from the equation ΔG_2 = BDE – TΔS (BDE = 87.54 kcal mol^{-1} from the reference [[10]] (experimental value), ΔS = 0.026 kcal mol^{-1} from the reference [[10]]).
(4)	K-PHI(e^-/H^+) = K-PHI + H•	$\Delta G_4 < 5.2$	Gas phase, 298 K. Estimated based on values of ΔG_2 and ΔG_3.

Table S15. Correlation of SCNPs doping degree with the yield of **2a** under the optimized conditions.

**Entry**	**SCNP**	**Diameter, nm**	**Doping degree, [electrons]·cm^{-3}**	**Yield of 2a, %**
1	K-PHI	100	ca. 10^{21}[a]	100%

2	mpg-CN	–	ca. $6 \cdot 10^{19}$[a]	12%
3	TiO_2	3	ca. $3\text{-}5 \cdot 10^{21}$[b]	Traces[d]
4	ZnO	4.6	ca.$2 \cdot 10^{19}$–$4 \cdot 10^{20}$[c]	–[e]

[a] Calculated using the equation: $N = d \cdot C \cdot N_A$,where d – density of carbon nitride, 2.336 g cm^{-3}. [16] C – concentration of electrons in the carbon nitride, mol g^{-1}. Data is taken from the reference [[17]]. N_A – Avogadro number, $6.02 \cdot 10^{23}$ mol^{-1}. [b] Data is taken from the reference [[18]]. [c] Data is taken from the references [[19]],[[20]]. [d] Commercial TiO_2. [e] Not evaluated in this work.

Crude ^{1}H NMR of aryl halides dehalogenation (1a-n)

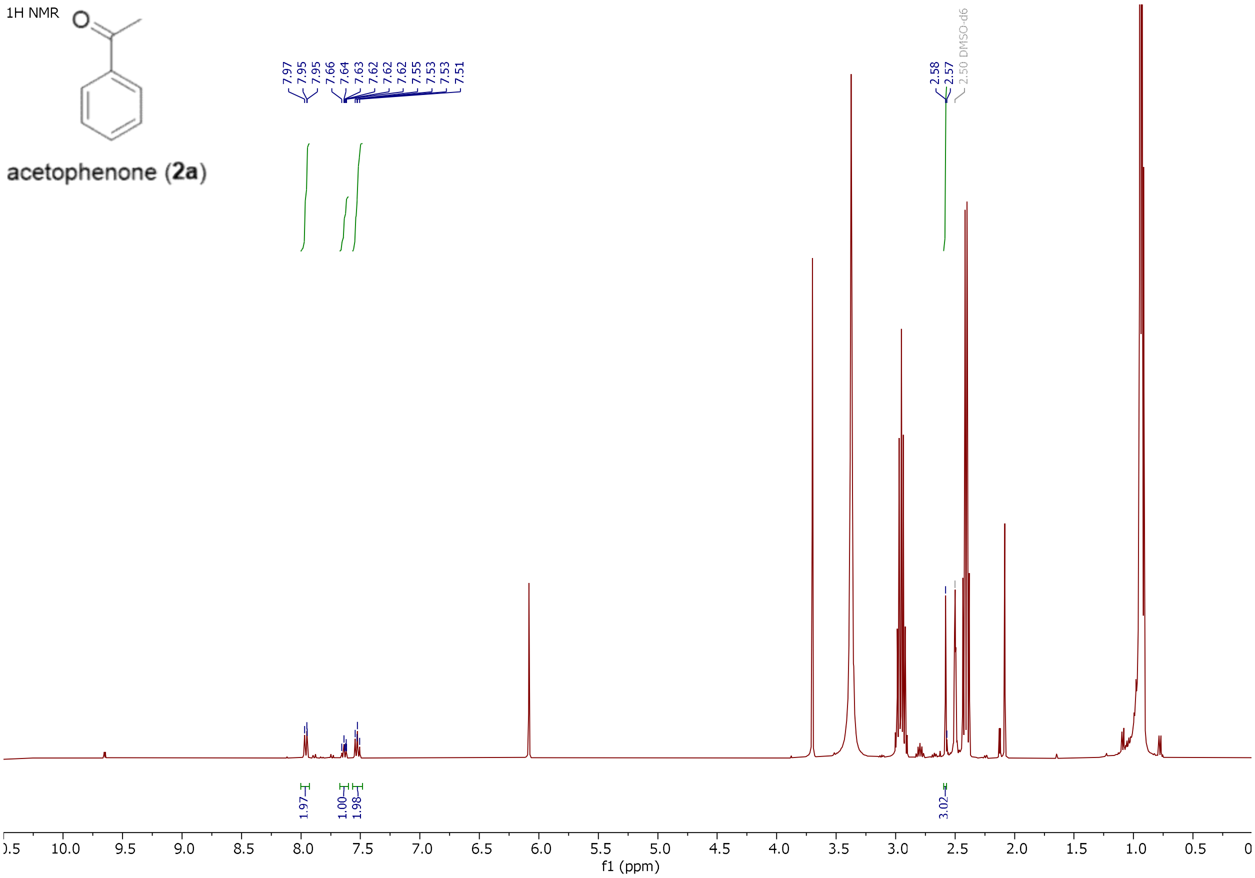

*Figure S 11 – Crude ^{1}H NMR of dehalogenation of **1a***

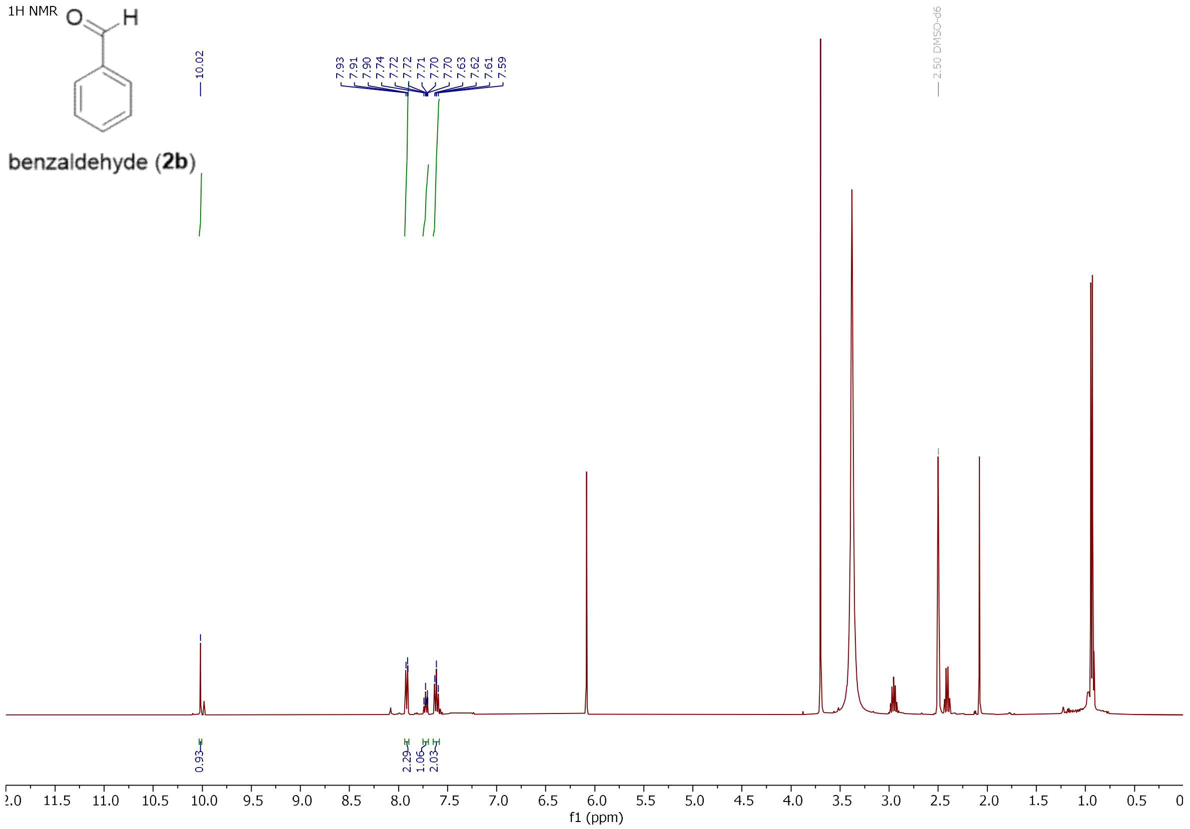

*Figure S 12 - Crude ¹H NMR of dehalogenation of **1b***

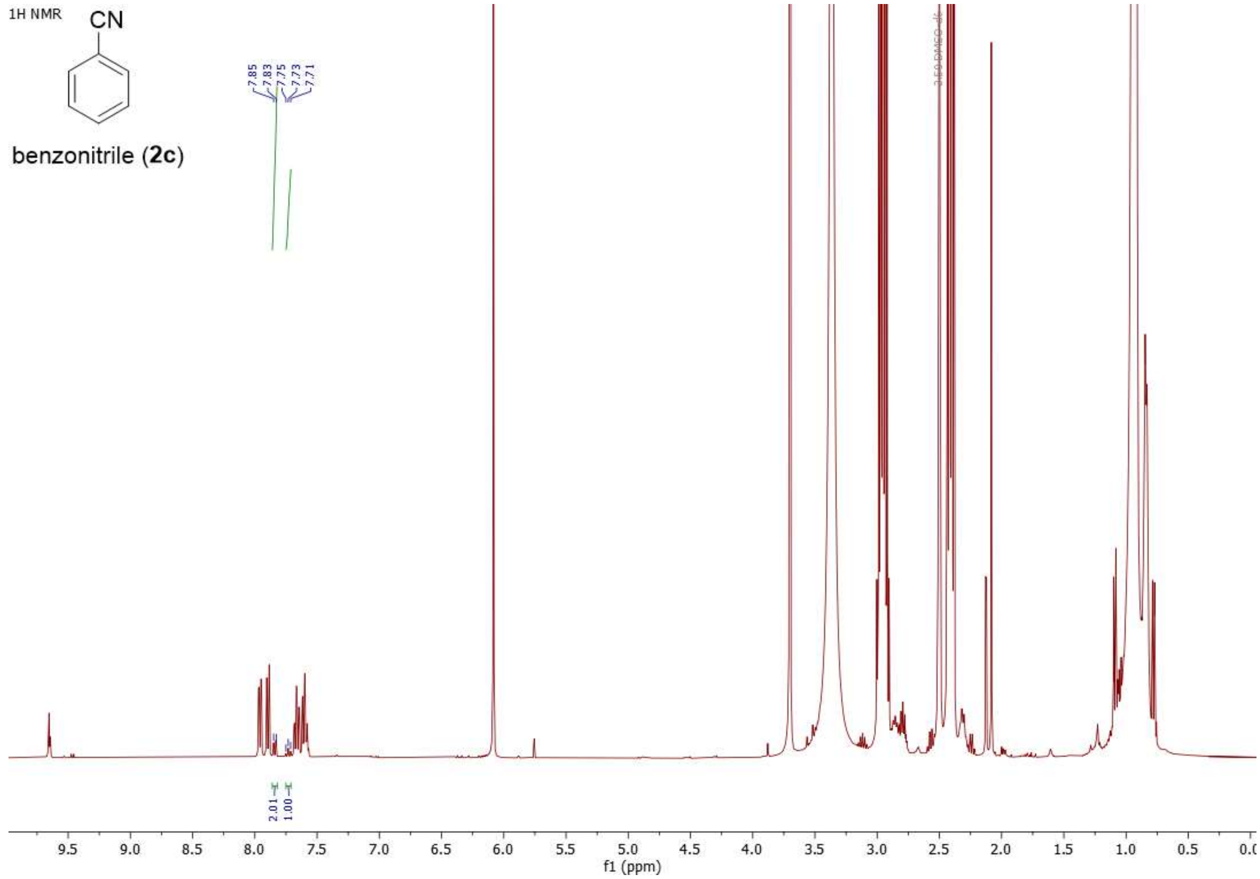

*Figure S 13 - Crude ^{1}H NMR of dehalogenation of **1c***

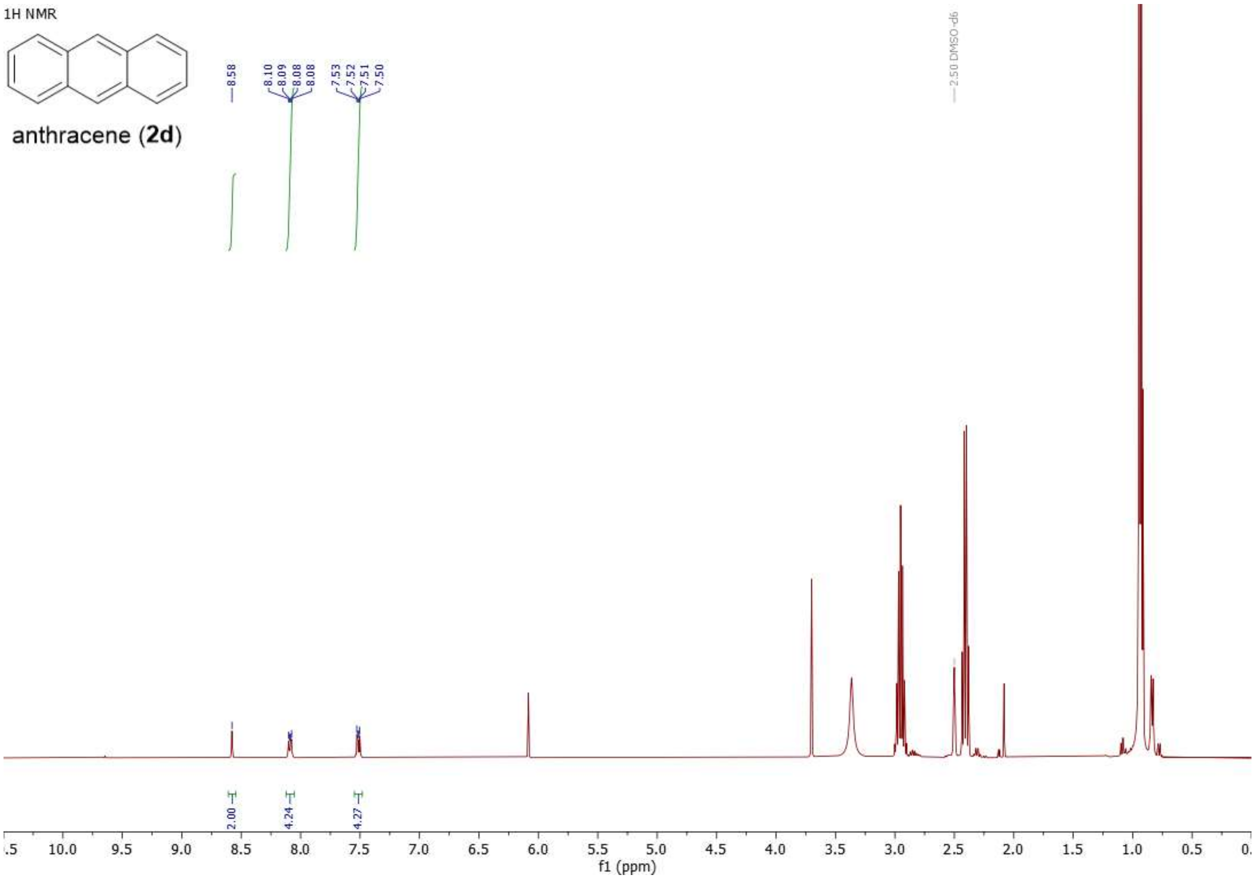

*Figure S 14 - Crude ^{1}H NMR of dehalogenation of **1d***

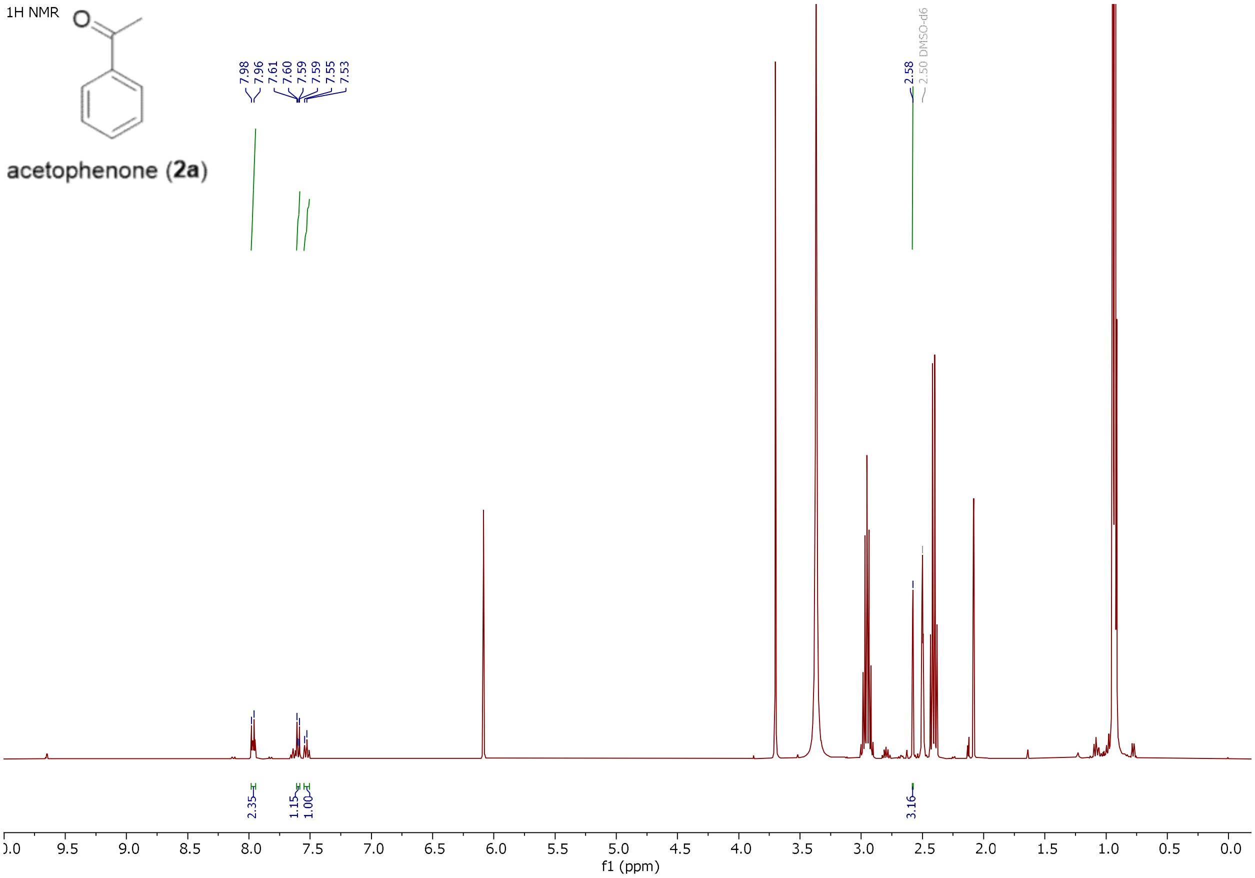

*Figure S 15 - Crude ^{1}H NMR of dehalogenation of **1e***

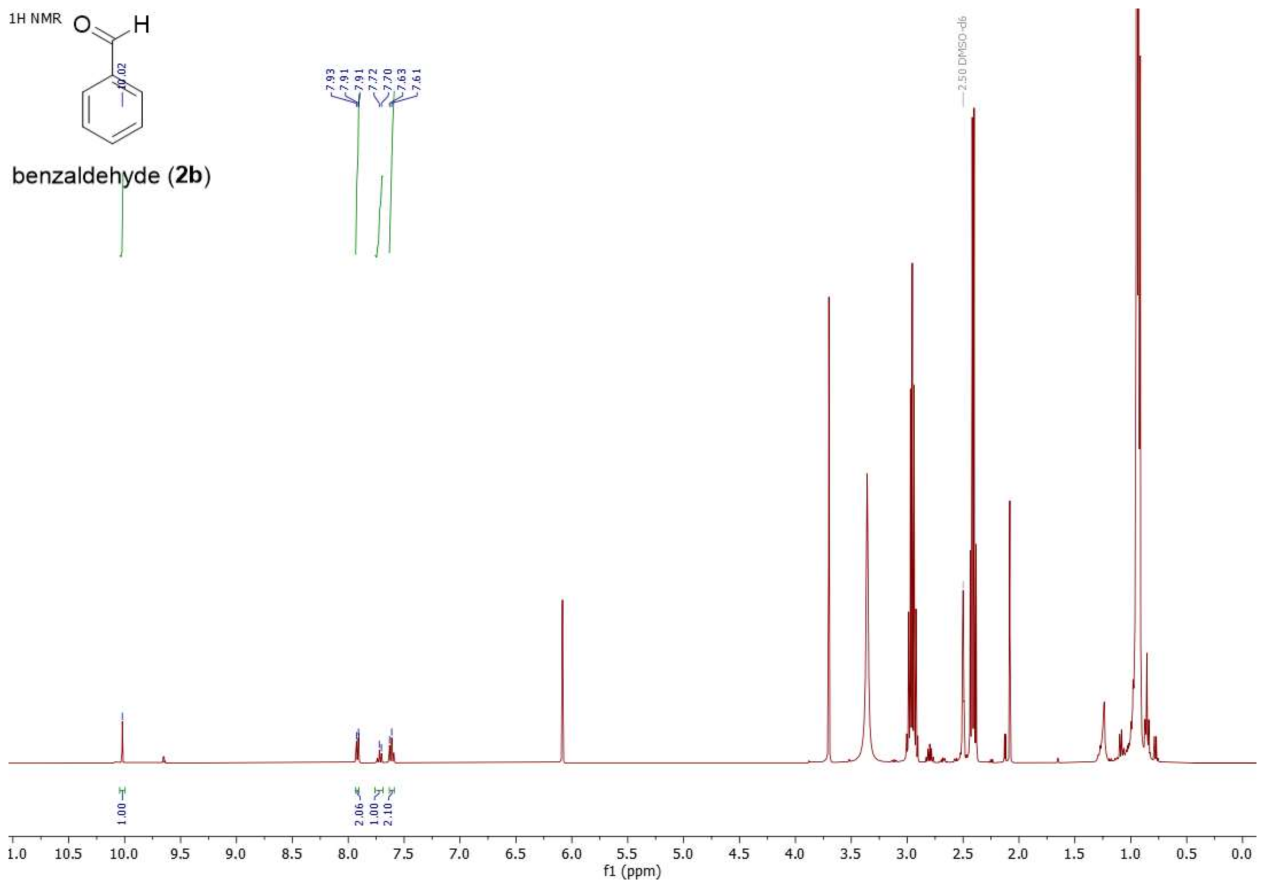

*Figure S 16 - Crude ^{1}H NMR of dehalogenation of **1f***

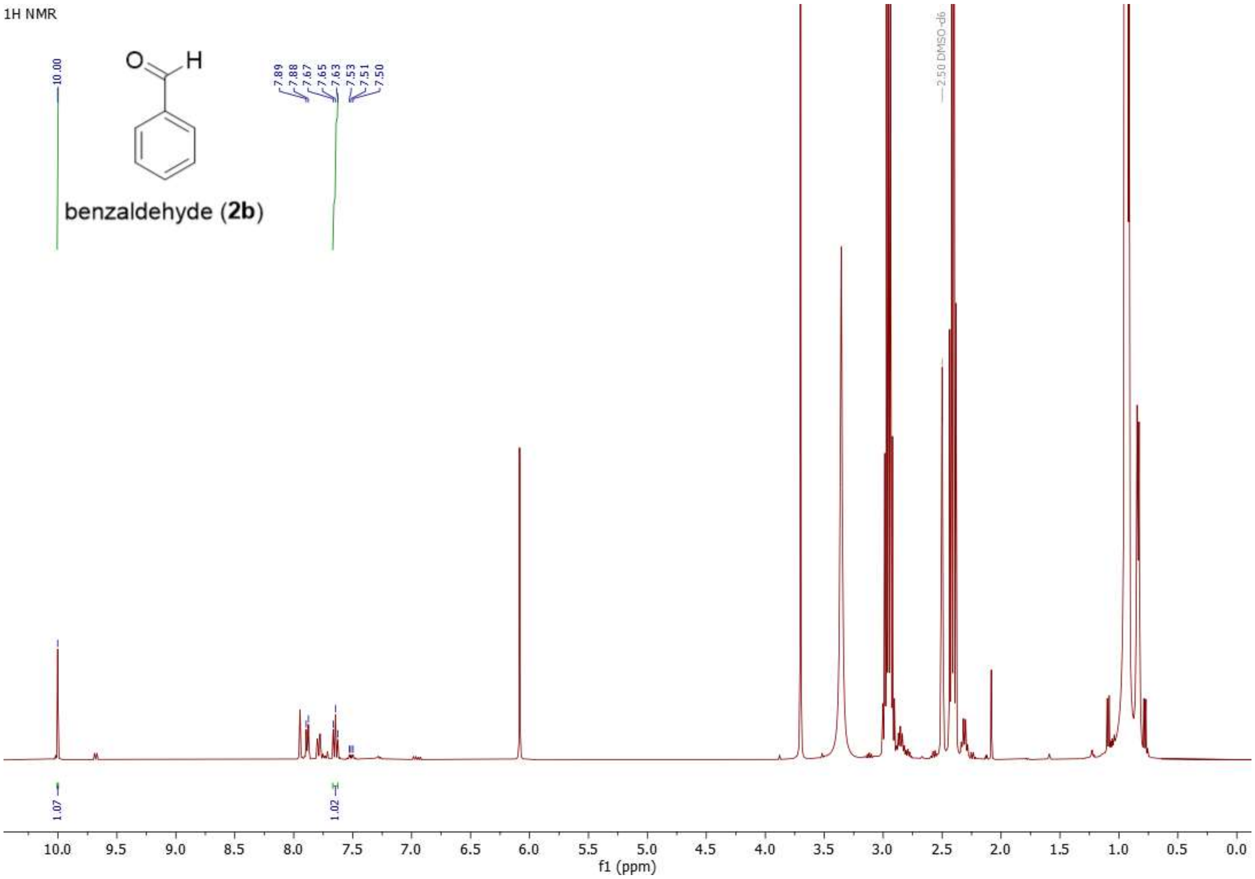

*Figure S 17 - Crude ^{1}H NMR of dehalogenation of **1g***

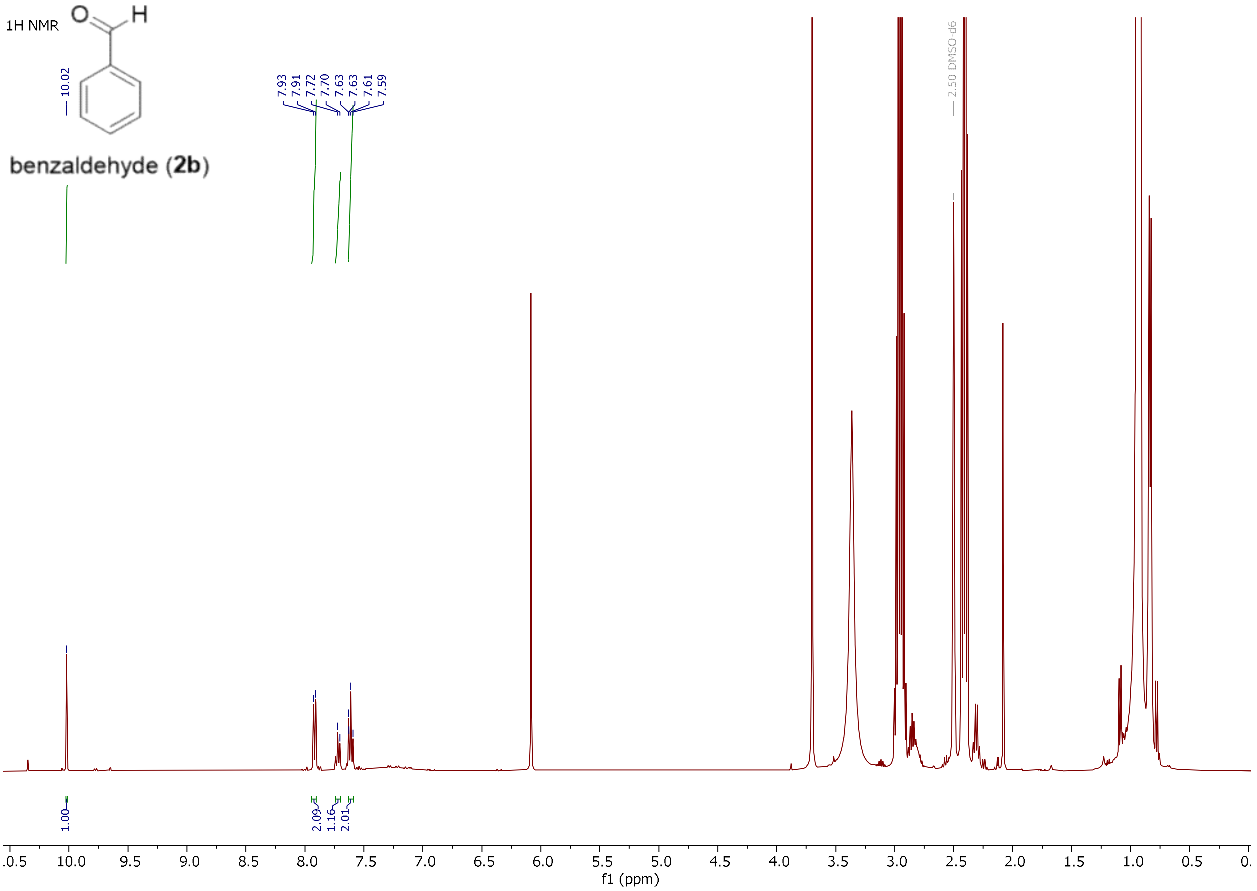

*Figure S 18 - Crude ^{1}H NMR of dehalogenation of **1h***

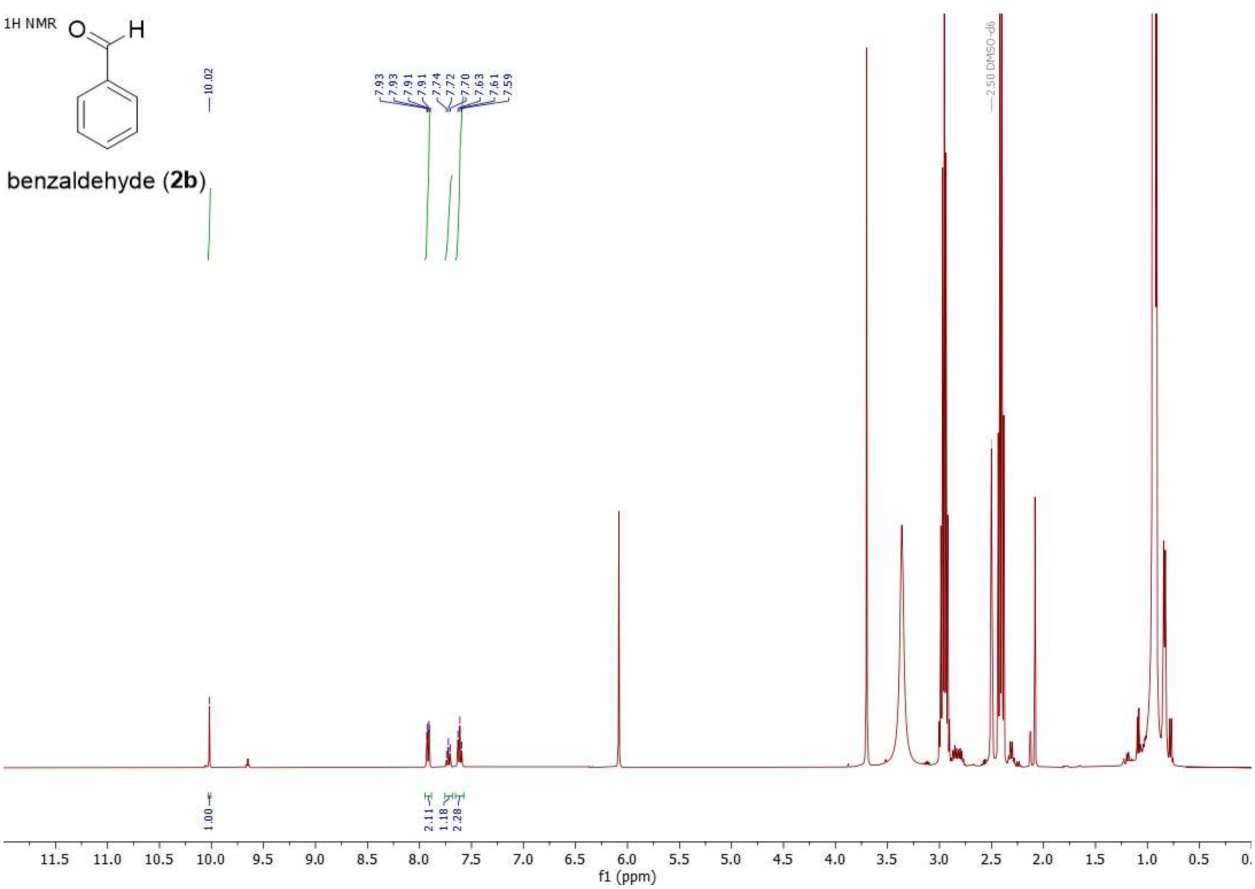

*Figure S 19 - Crude ^{1}H NMR of dehalogenation of **1i***

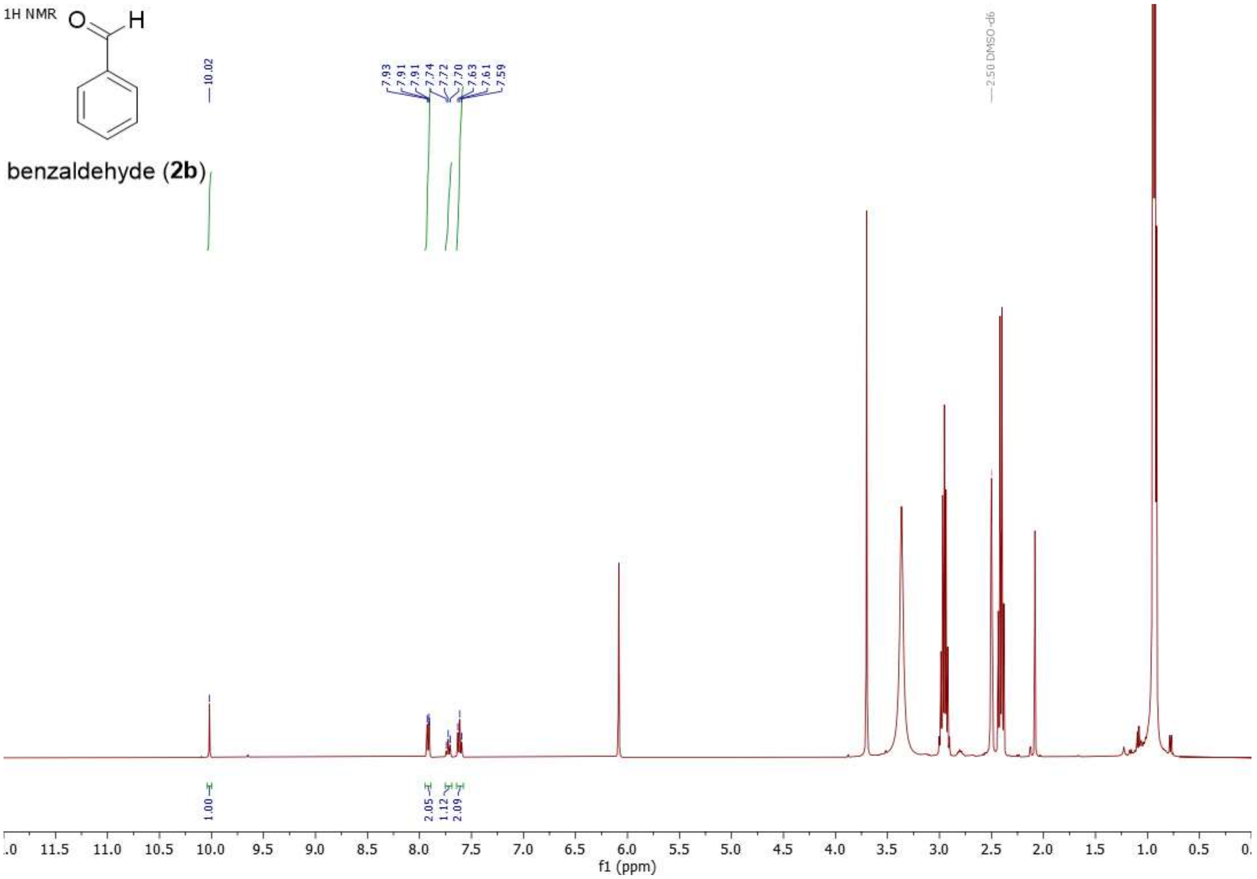

*Figure S 20 - Crude ^{1}H NMR of dehalogenation of **1j***

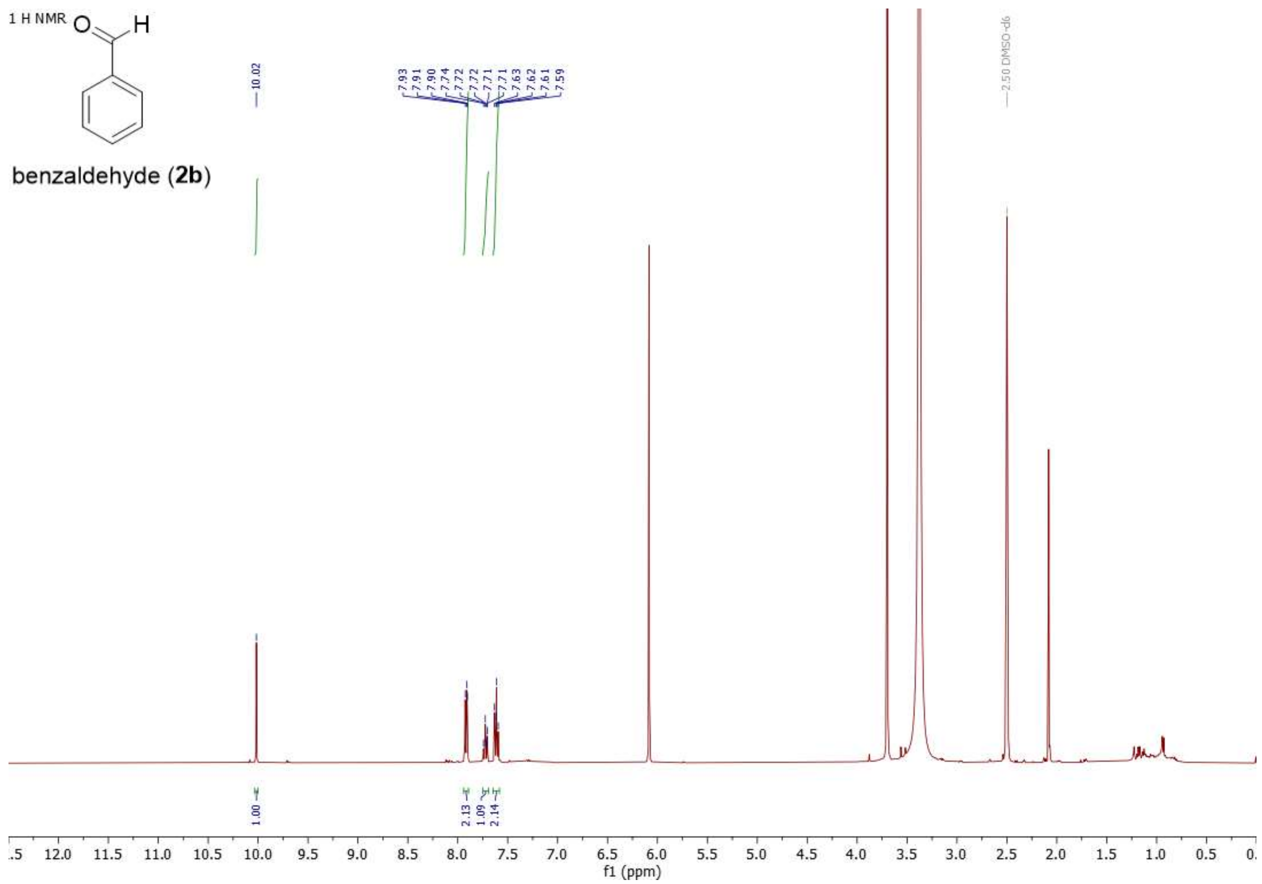

*Figure S 21 - Crude ¹H NMR of dehalogenation of **1k***

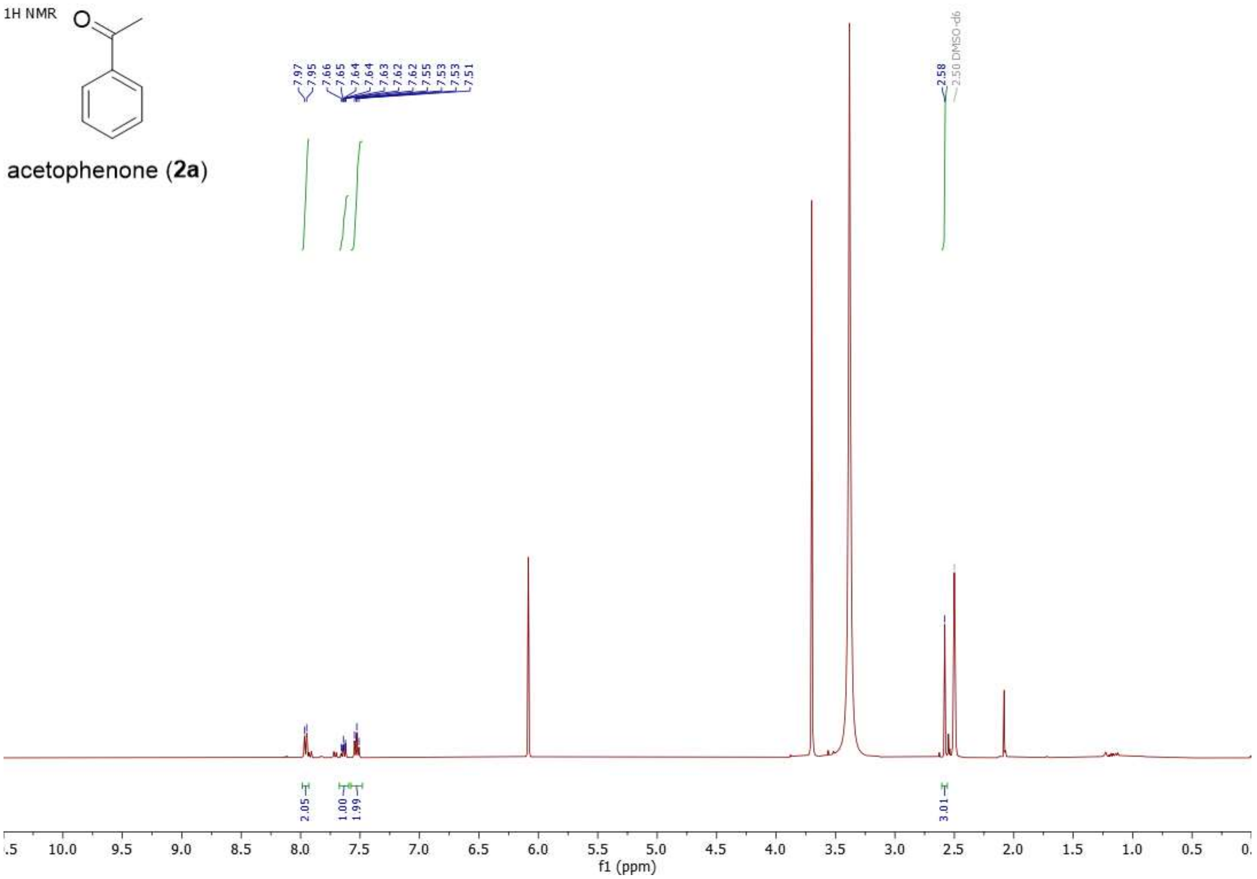

Figure S 22 - Crude ^{1}H NMR of dehalogenation of ***1l***

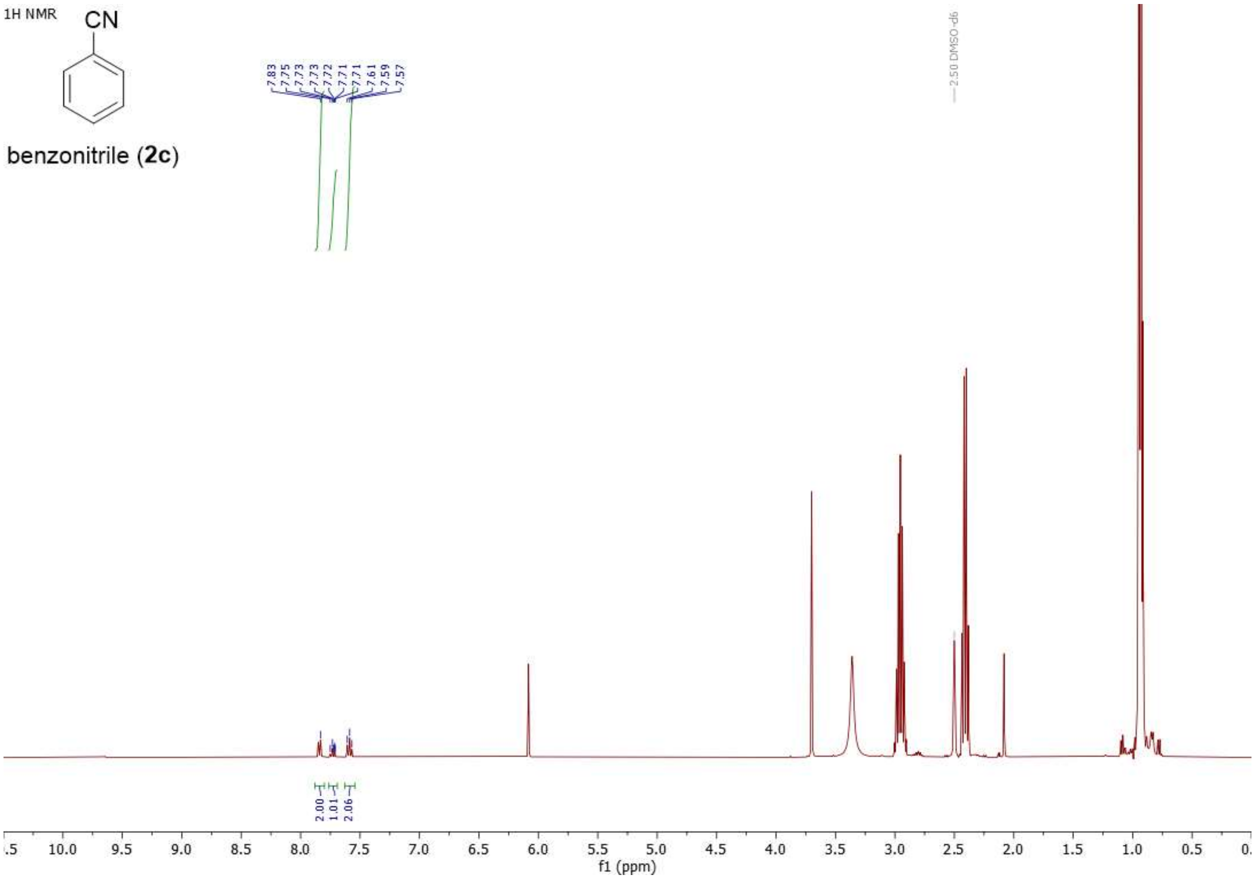

Figure S 23 - Crude ^{1}H NMR of dehalogenation of ***1m***

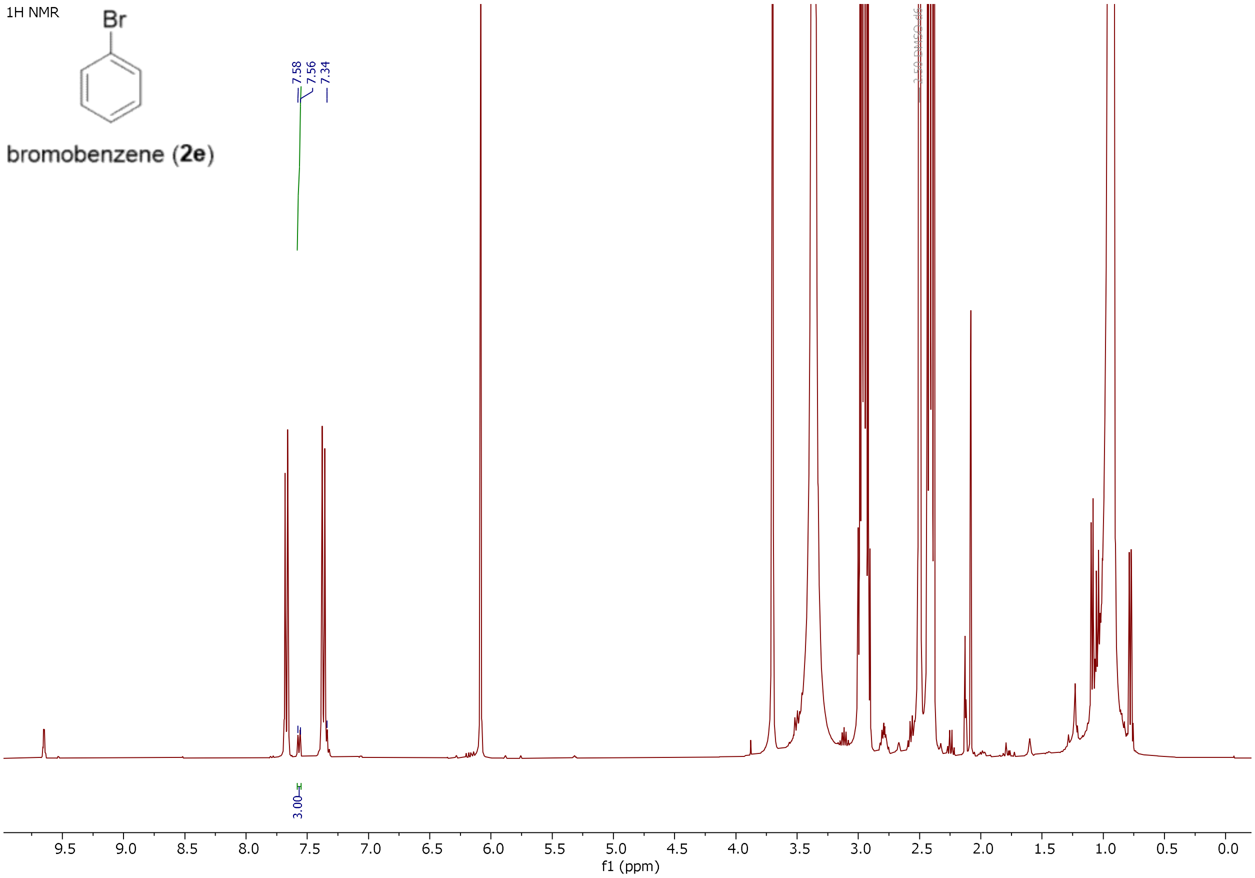

*Figure S 24 - Crude ¹H NMR of dehalogenation of **1n***

^{1}H NMR and ^{13}C NMR of reference compounds

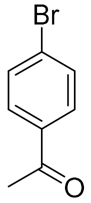

1-(4-bromophenyl)ethan-1-one (**1a**)

^{1}H NMR (400 MHz, DMSO) δ 7.87 (s, 2H), 7.76 – 7.68 (m, 2H), 2.57 (s, 3H).

^{13}C NMR (101 MHz, DMSO) δ 197.20, 135.78, 131.77, 130.24, 127.35, 26.76.

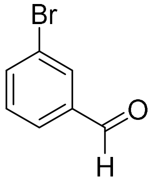

3-bromobenzaldehyde (**1b**)

^{1}H NMR (400 MHz, DMSO) δ 9.94 (s, 1H), 7.95 (s, 1H), 7.83 (dd, *J* = 7.6, 1.4 Hz, 1H), 7.79 – 7.72 (m, 1H), 7.47 (t, *J* = 7.8 Hz, 1H).

^{13}C NMR (101 MHz, DMSO) δ 191.63, 137.90, 136.87, 131.81, 131.09, 128.20, 122.47.

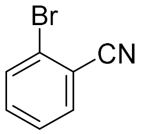

2-bromobenzonitrile (**1c**)

1H NMR (400 MHz, DMSO) δ 7.95 (dt, J = 7.7, 2.0 Hz, 1H), 7.89 (dq, J = 8.1, 1.3 Hz, 1H), 7.69 – 7.56 (m, 2H).

^{13}C NMR (101 MHz, DMSO) δ 135.16, 135.00, 133.21, 128.55, 124.52, 117.31, 114.44.

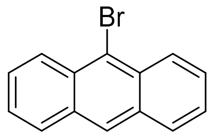

9-bromoanthracene (**1d**)

^{1}H NMR (400 MHz, DMSO) δ 8.72 (s, 1H), 8.40 (d, *J* = 8.8 Hz, 2H), 8.16 (d, *J* = 8.4 Hz, 2H), 7.74 – 7.67 (m, 2H), 7.64 – 7.56 (m, 2H).

^{13}C NMR (101 MHz, DMSO) δ 131.77, 129.83, 128.92, 128.10, 127.61, 126.73, 126.04, 121.02.

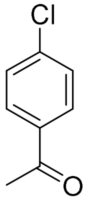

1-(4-chlorophenyl)ethan-1-one (**1e**)

^{1}H NMR (400 MHz, DMSO) δ 7.92 (d, J = 8.6 Hz, 2H), 7.52 (d, J = 8.6 Hz, 2H), 2.55 (s, 3H).

^{13}C NMR (101 MHz, DMSO) δ 196.77, 138.12, 135.38, 130.01, 128.71, 26.63.

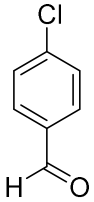

4-chlorobenzaldehyde (**1f**)

^{1}H NMR (400 MHz, DMSO) δ 9.99 (s, 1H), 7.92 (d, *J* = 8.5 Hz, 2H), 7.69 – 7.63 (m, 2H).

^{13}C NMR (101 MHz, DMSO) δ 192.17, 139.43, 134.85, 131.22, 129.40.

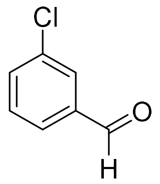

3-chlorobenzaldehyde (**1g**)

^{1}H NMR (400 MHz, DMSO) δ 10.02 – 9.92 (m, 1H), 7.95 – 7.69 (m, 3H), 7.66 – 7.42 (m, 1H).

^{13}C NMR (101 MHz, DMSO) δ 191.52, 137.74, 134.11, 133.93, 130.72, 128.71, 127.88.

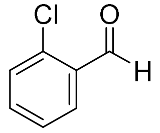

2-chlorobenzaldehyde (**1h**)

^{1}H NMR (400 MHz, DMSO) δ 10.35 – 10.21 (m, 1H), 7.85 (dd, *J* = 6.8, 4.8 Hz, 1H), 7.68 (d, *J* = 7.4 Hz, 1H), 7.61 (d, *J* = 8.2 Hz, 1H), 7.52 (s, 1H).

^{13}C NMR (101 MHz, DMSO) δ 189.20, 136.53, 135.46, 131.87, 130.54, 129.26, 127.57.

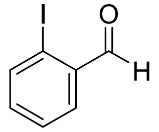

2-iodobenzaldehyde (**1i**)

^{1}H NMR (400 MHz, DMSO) δ 9.96 (d, J = 0.6 Hz, 1H), 8.09 – 8.02 (m, 1H), 7.83 – 7.75 (m, 1H), 7.58 (s, 1H), 7.42 (td, J = 7.6, 1.8 Hz, 1H).

^{13}C NMR (101 MHz, DMSO) δ 195.58, 140.72, 135.97, 134.98, 130.32, 128.98, 100.91.

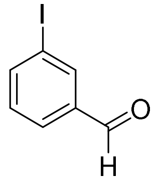

3-iodobenzaldehyde (**1j**)

^{1}H NMR (400 MHz, DMSO) δ 9.93 (s, 1H), 8.22 (s, 1H), 8.05 (dd, *J* = 7.8, 0.7 Hz, 1H), 7.90 (d, *J* = 7.6 Hz, 1H), 7.40 (t, *J* = 7.7 Hz, 1H).

^{13}C NMR (101 MHz, DMSO) δ 192.16, 142.86, 138.06, 137.93, 131.31, 128.74, 95.50.

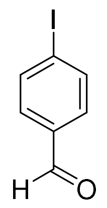

4-iodobenzaldehyde (**1k**)

^{1}H NMR (400 MHz, DMSO) δ 9.96 (s, 1H), 8.01 (d, *J* = 8.3 Hz, 2H), 7.66 (d, *J* = 8.3 Hz, 2H).

^{13}C NMR (101 MHz, DMSO) δ 192.81, 138.19, 135.40, 130.97, 103.71.

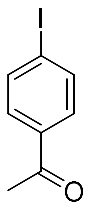

1-(4-iodophenyl)ethan-1-one (**1l**)

^{1}H NMR (400 MHz, DMSO) δ 7.91 (d, *J* = 8.5 Hz, 2H), 7.71 (d, *J* = 8.5 Hz, 2H), 2.55 (s, 3H).

^{13}C NMR (101 MHz, DMSO) δ 197.56, 137.66, 136.08, 129.96, 101.92, 26.70.

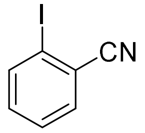

2-iodobenzonitrile (**1m**)

^{1}H NMR (400 MHz, DMSO) δ 8.09 – 8.02 (m, 1H), 7.90 – 7.82 (m, 1H), 7.59 (d, *J* = 1.1 Hz, 1H), 7.45 (d, *J* = 1.7 Hz, 1H).

^{13}C NMR (101 MHz, DMSO) δ 139.33, 134.62, 128.81, 119.62, 119.14, 100.10.

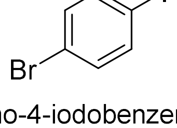

1-bromo-4-iodobenzene (**1n**)

^{1}H NMR (400 MHz, DMSO) δ 7.67 (d, *J* = 8.5 Hz, 2H), 7.36 (d, *J* = 8.4 Hz, 2H).

^{13}C NMR (101 MHz, DMSO) δ 139.27, 133.54, 121.55, 93.48.

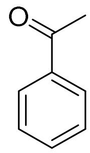

acetophenone (**2a**)

^{1}H NMR (400 MHz, DMSO) δ 7.96 (d, *J* = 6.4 Hz, 2H), 7.62 (d, *J* = 7.0 Hz, 1H), 7.55 – 7.47 (m, 2H), 2.59 – 2.54 (m, 3H).

^{13}C NMR (101 MHz, DMSO) δ 197.82, 136.80, 133.13, 128.64, 128.16, 26.62.

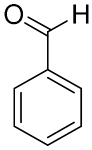

benzaldehyde (**2b**)

^{1}H NMR (400 MHz, DMSO) δ 10.02 (s, 1H), 7.90 (dt, *J* = 8.4, 1.5 Hz, 2H), 7.66 (s, 1H), 7.60 – 7.51 (m, 2H).

^{13}C NMR (101 MHz, DMSO) δ 192.94, 136.21, 134.43, 129.46, 129.04.

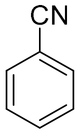

benzonitrile (**2c**)

^{1}H NMR (400 MHz, DMSO) δ 7.78 (s, 2H), 7.73 – 7.62 (m, 1H), 7.54 (s, 2H).

^{13}C NMR (101 MHz, DMSO) δ 133.17, 132.14, 129.36, 118.78, 111.44.

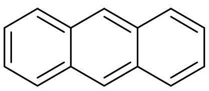

anthracene (**2d**)

^{1}H NMR (400 MHz, DMSO) δ 8.59 (s, 2H), 8.10 (dd, *J* = 6.5, 3.3 Hz, 4H), 7.52 (dd, *J* = 6.6, 3.2 Hz, 4H).

^{13}C NMR (101 MHz, DMSO) δ 131.23, 128.08, 126.05, 125.62.

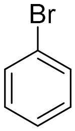

bromobenzene (**2e**)

^{1}H NMR (400 MHz, DMSO) δ 7.53 (d, *J* = 8.1 Hz, 2H), 7.38 – 7.23 (m, 3H).

^{13}C NMR (101 MHz, DMSO) δ 131.24, 130.31, 127.10, 121.90.

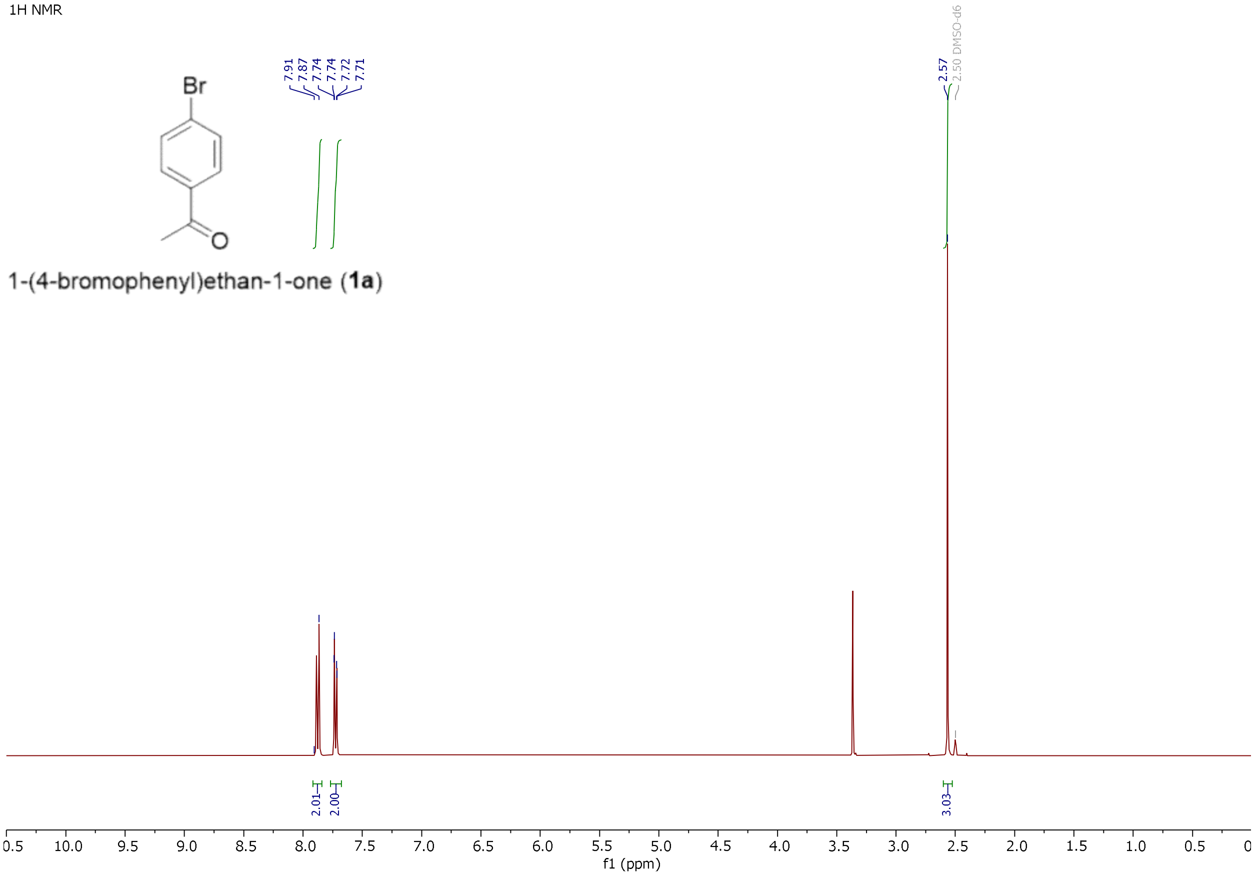

*Figure S 25 - ^{1}H NMR of **1a***

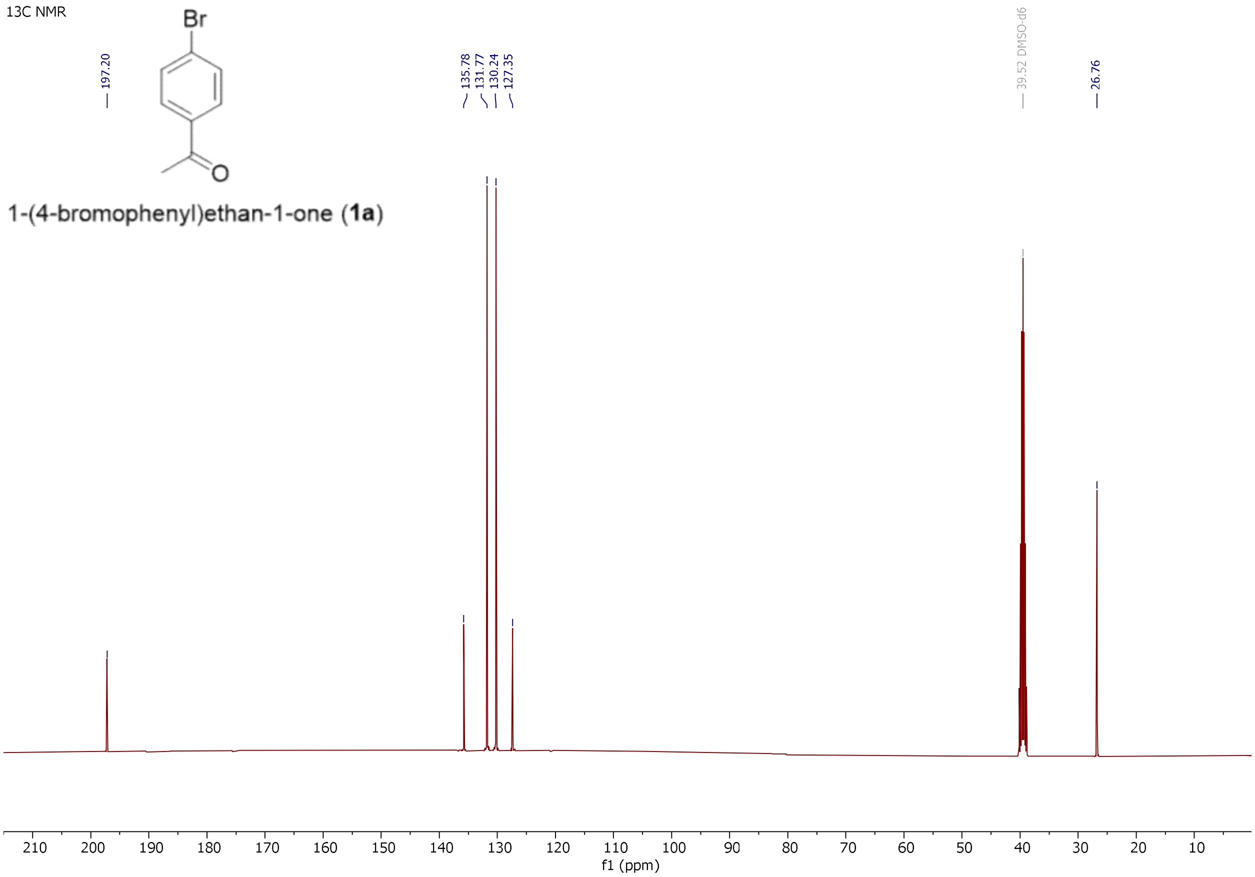

*Figure S 26 – ^{13}C NMR of **1a***

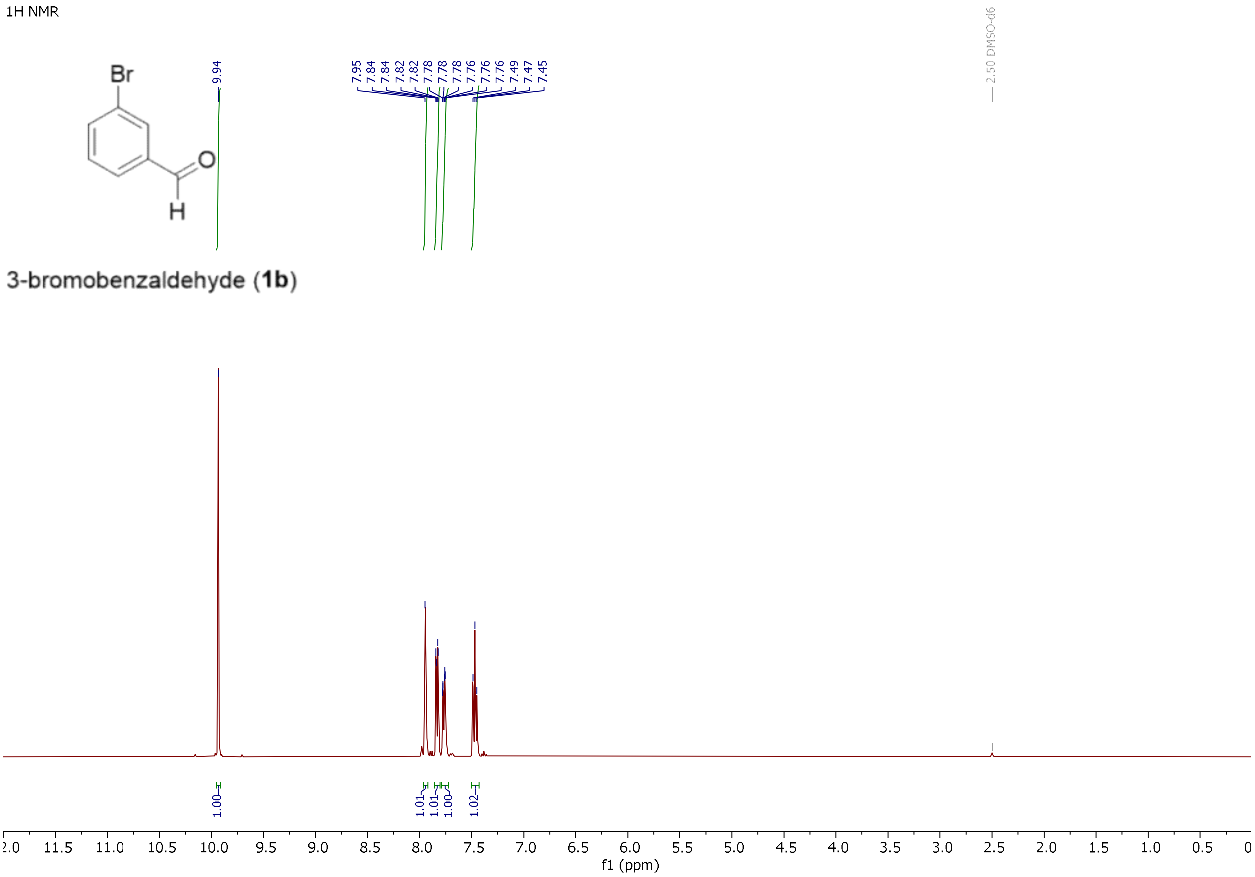

*Figure S 27 - ¹H NMR of 1**b***

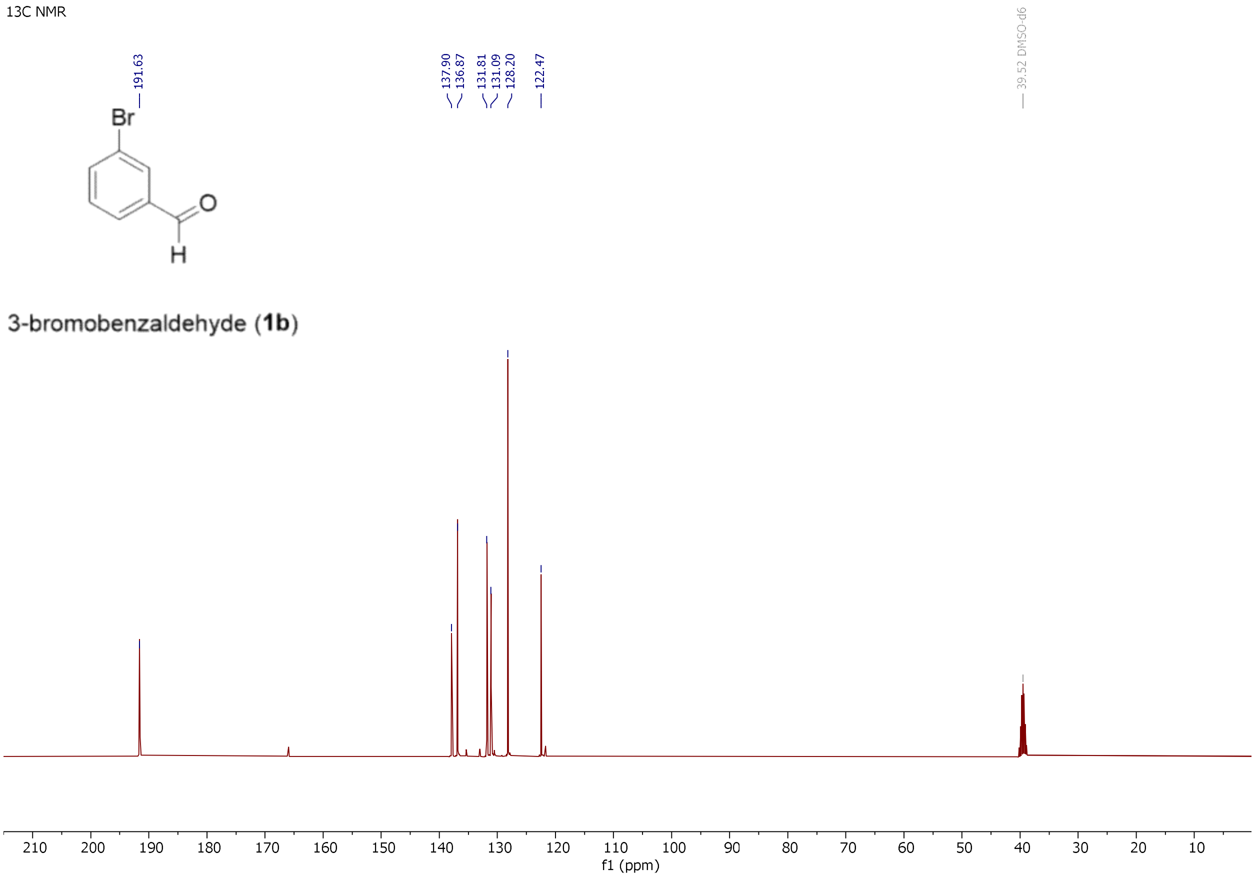

*Figure S 28 - ^{13}C NMR of **1b***

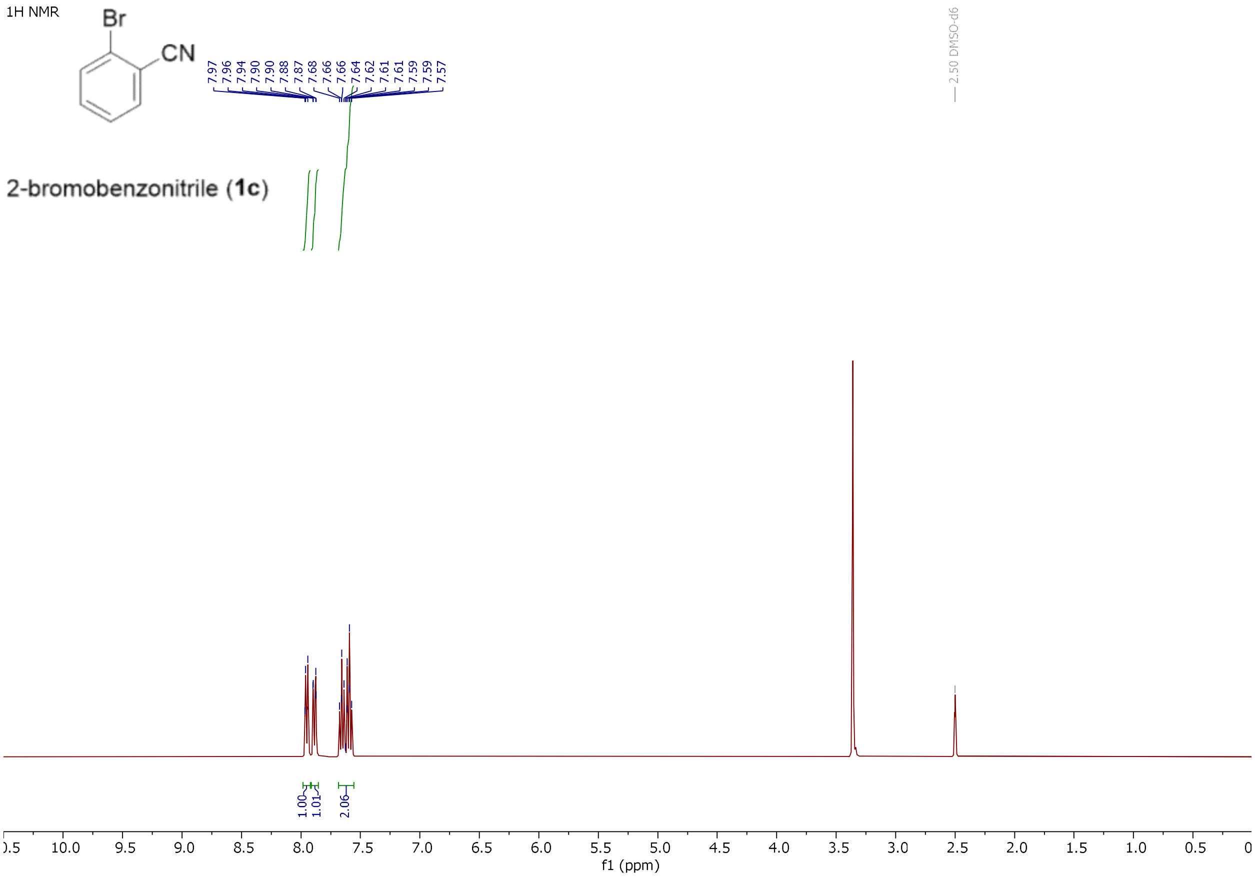

*Figure S 29 - ¹H NMR of 1**c***

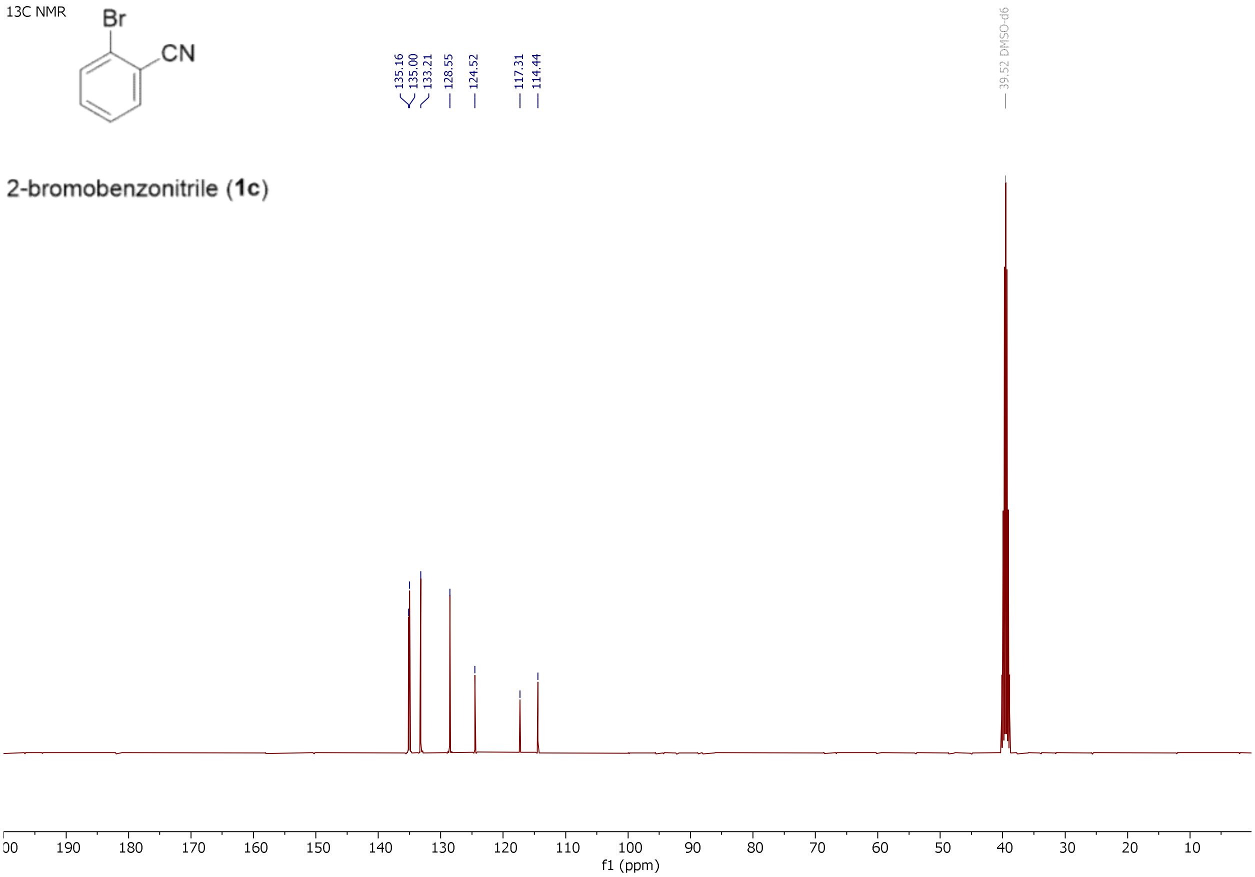

*Figure S 30 - ^{13}C NMR of **1c***

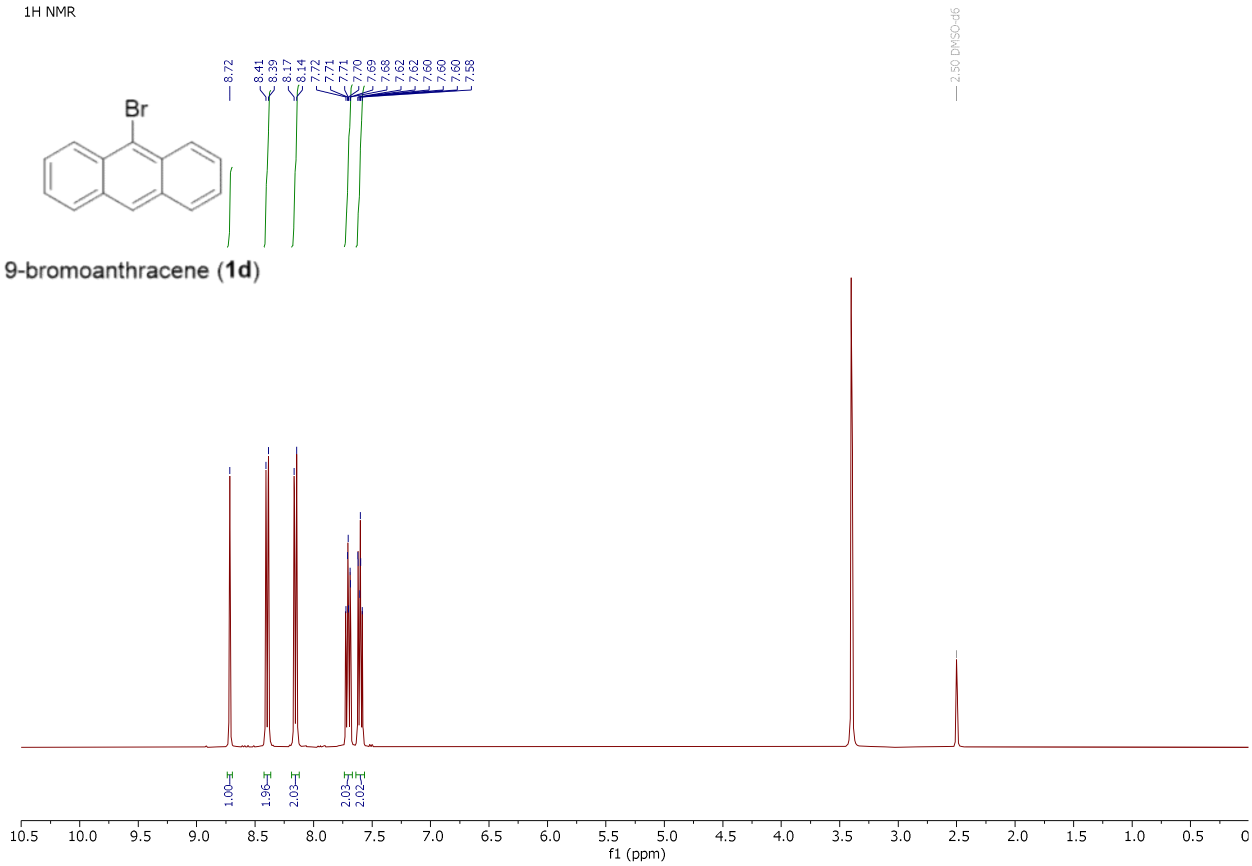

*Figure S 31 - ^{1}H NMR of **1d***

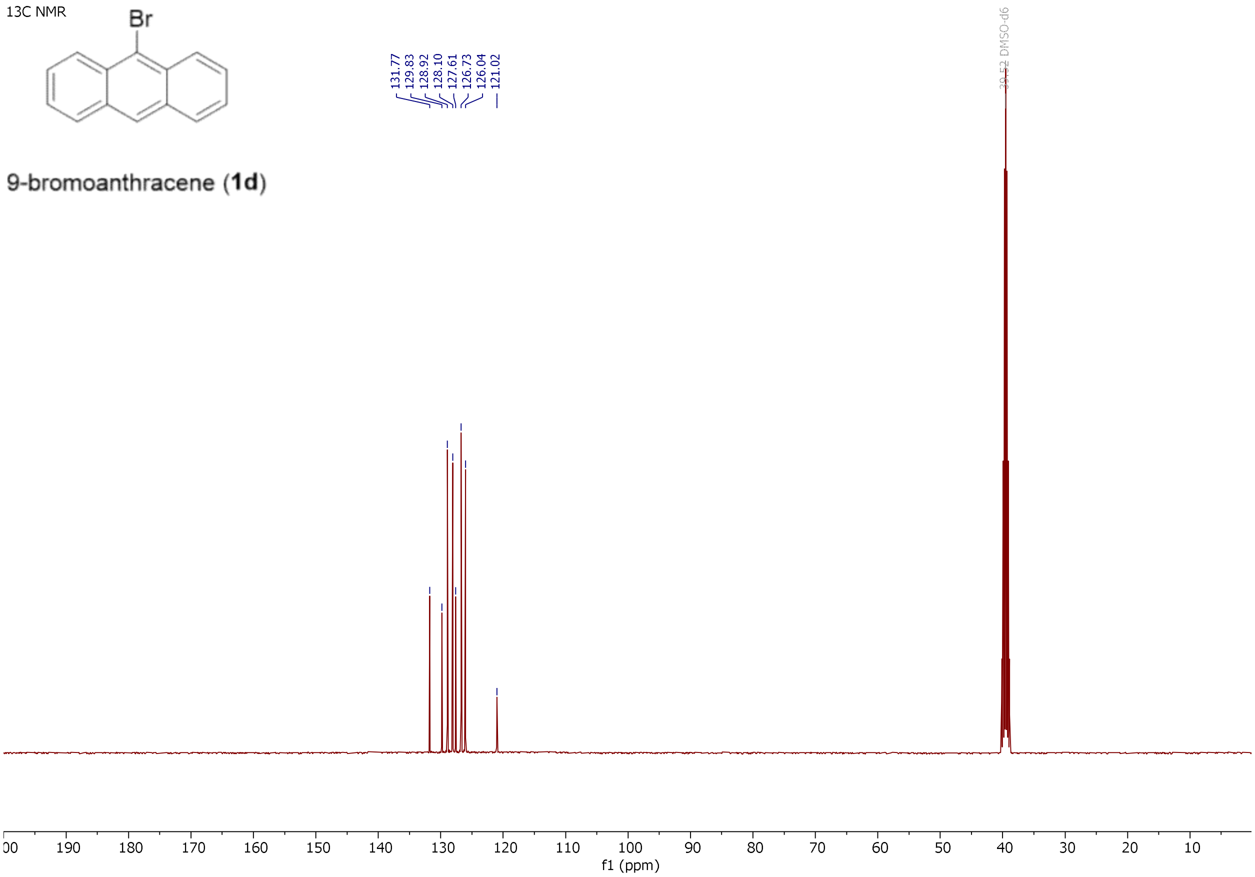

*Figure S 32 - ^{13}C NMR of **1d***

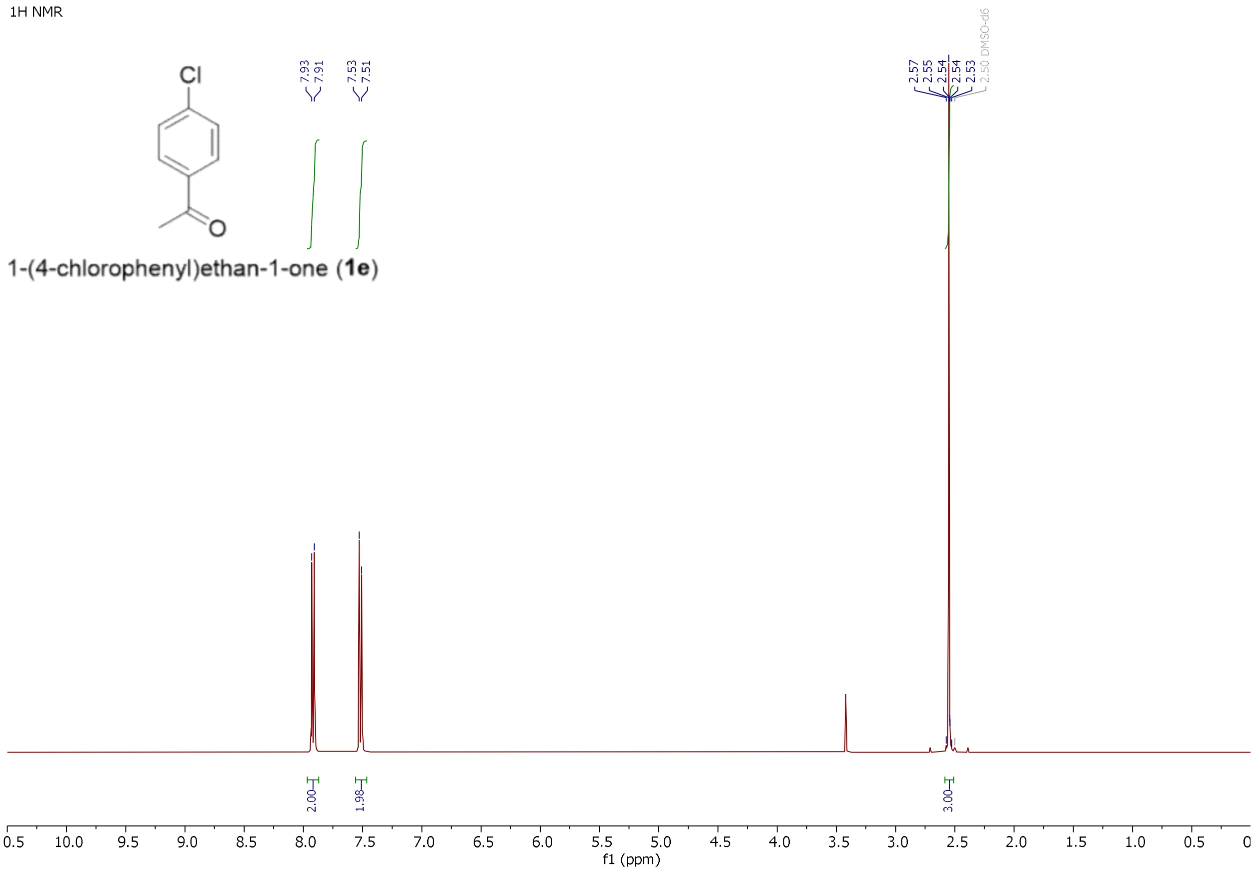

*Figure S 33 - ¹H NMR of **1e***

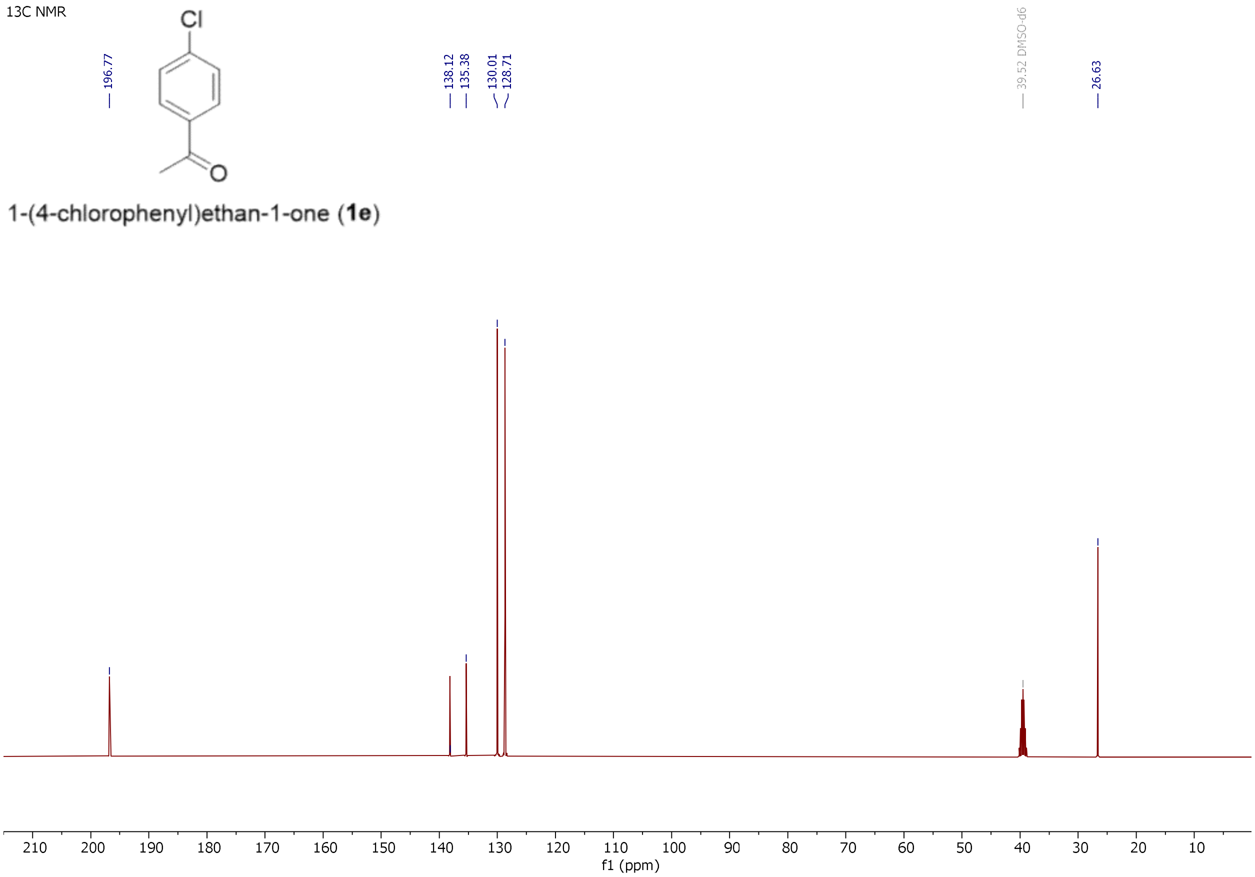

*Figure S 34 - ^{13}C NMR of **1e***

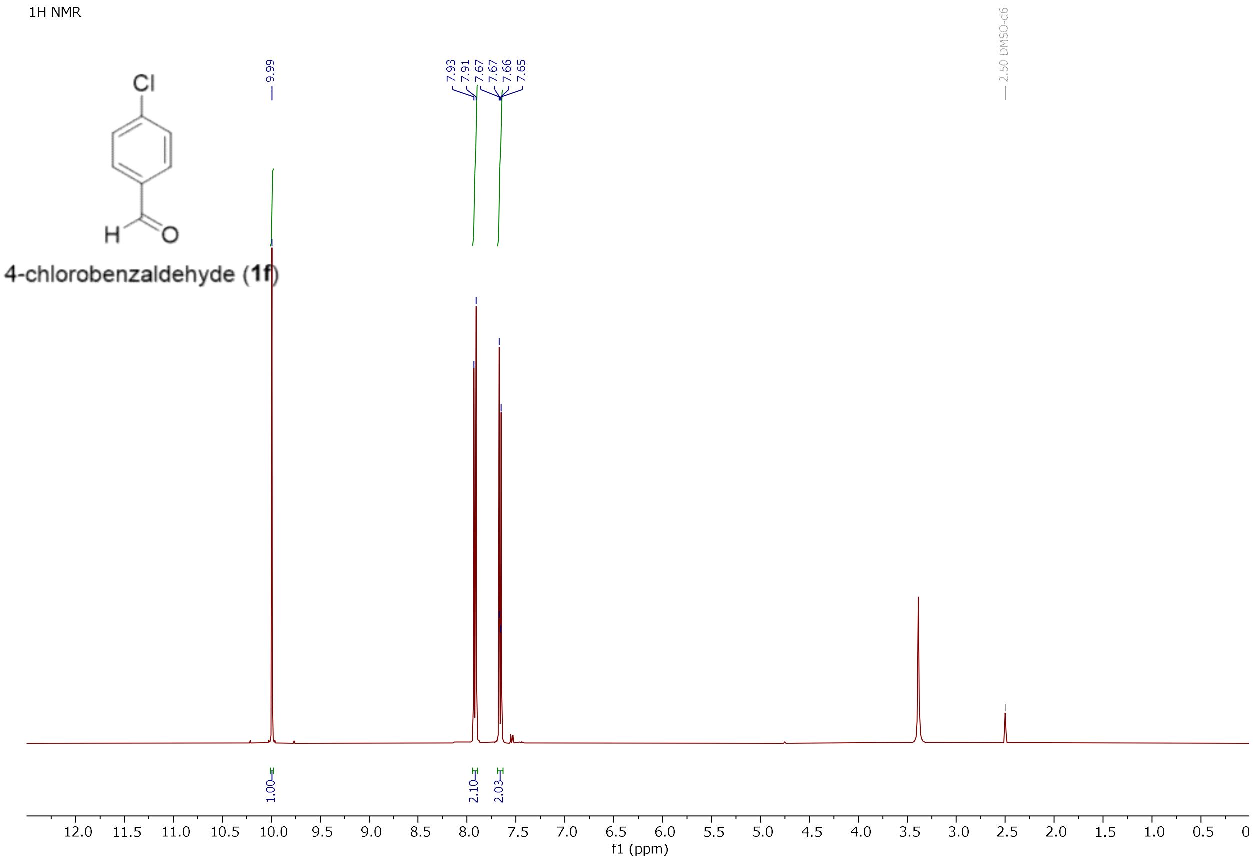

*Figure S 35 - ^{1}H NMR of **1f***

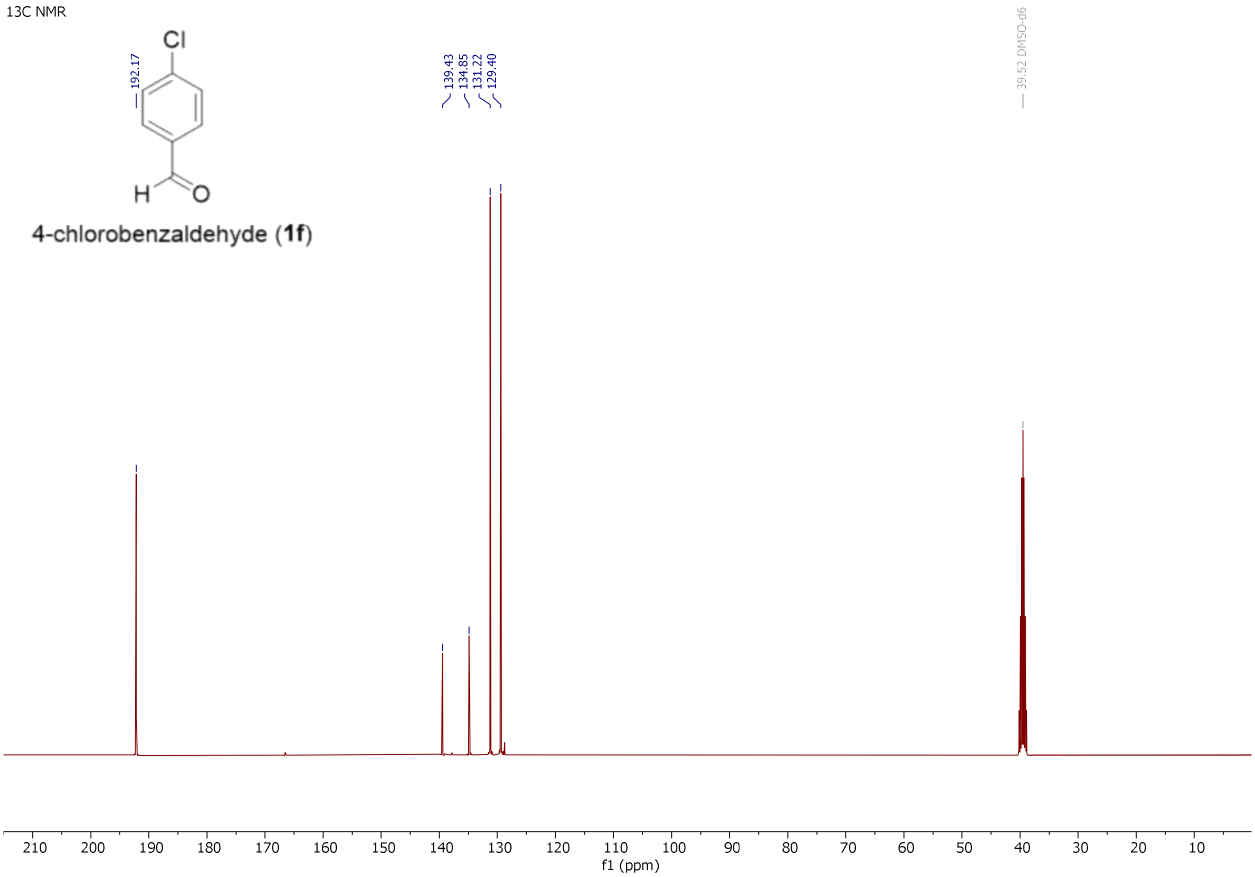

*Figure S 36 - ^{13}C NMR of **1f***

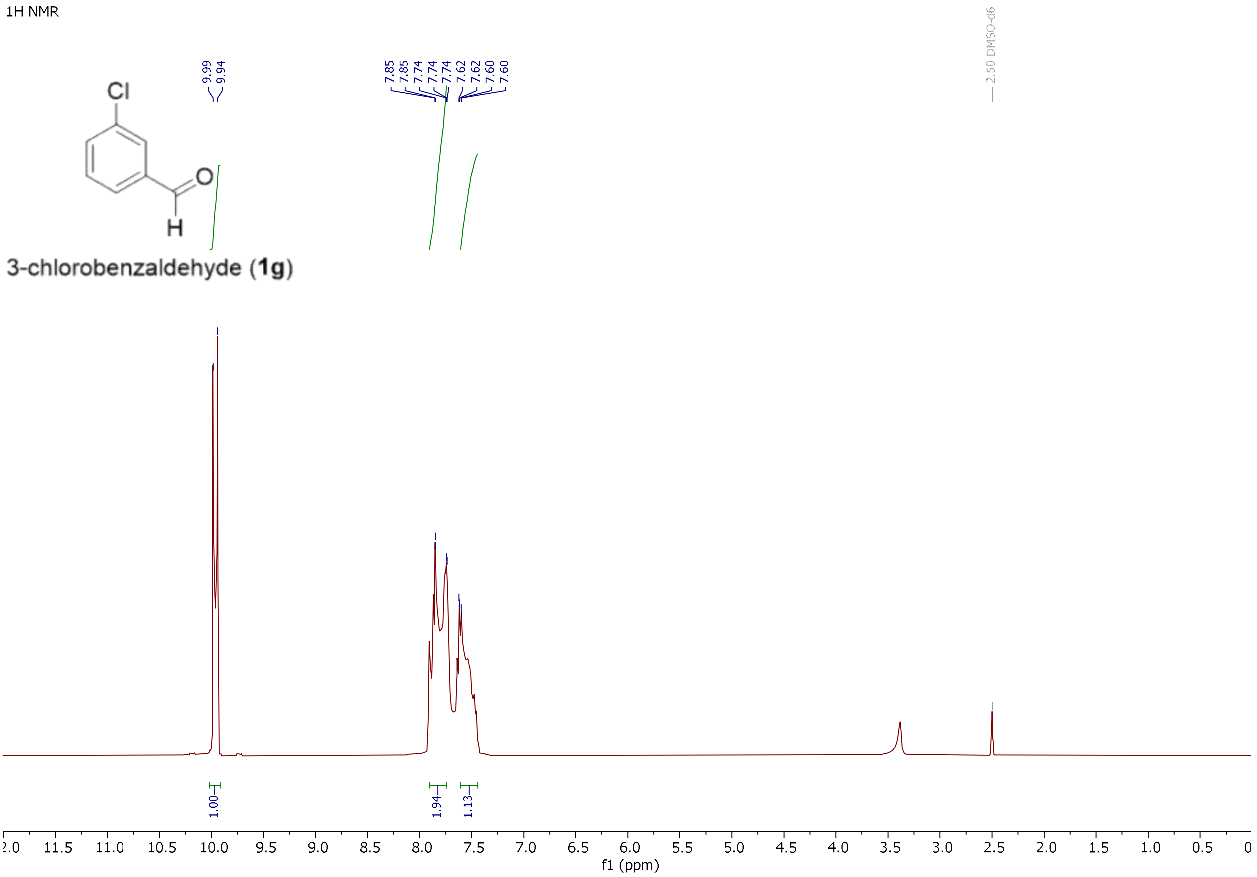

*Figure S 37 - ¹H NMR of **1g***

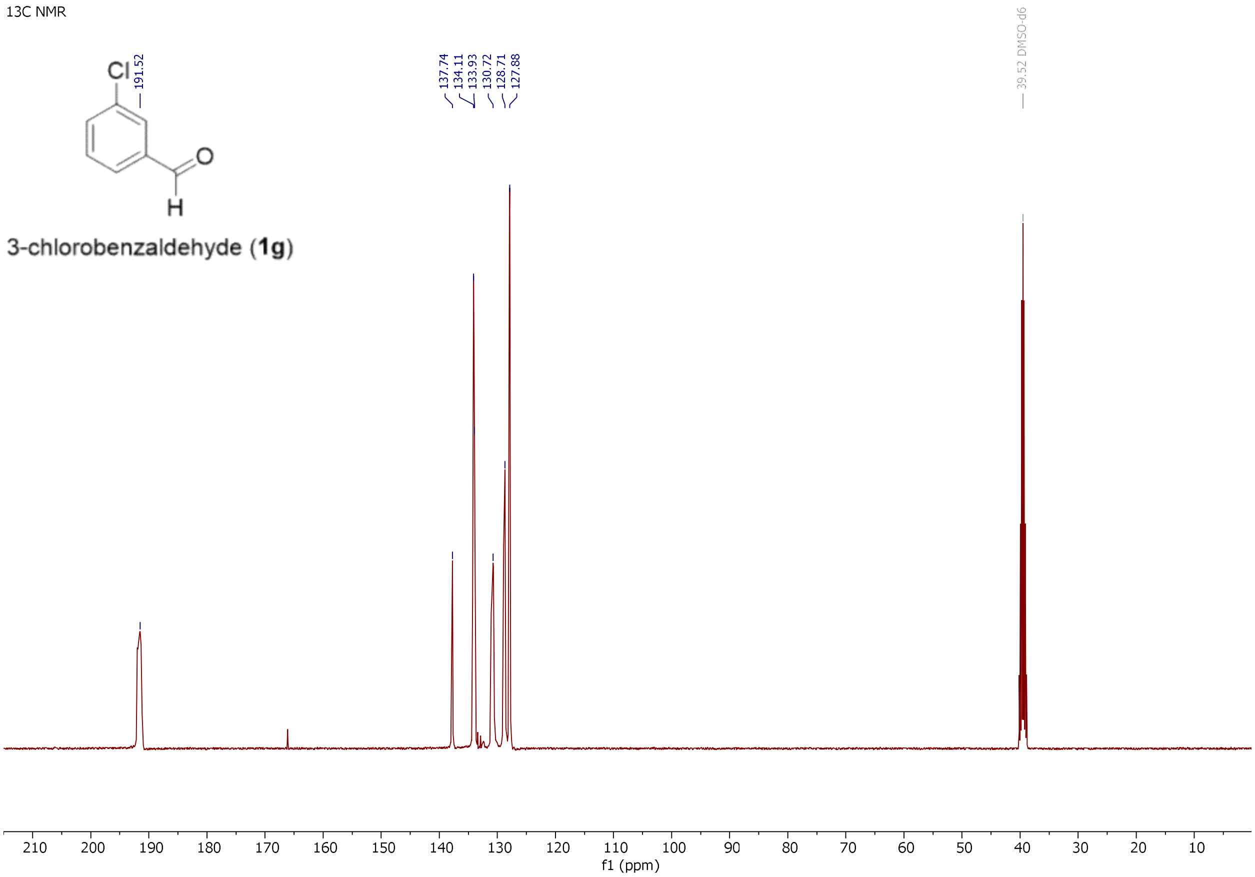

*Figure S 38 - ^{13}C NMR of **1g***

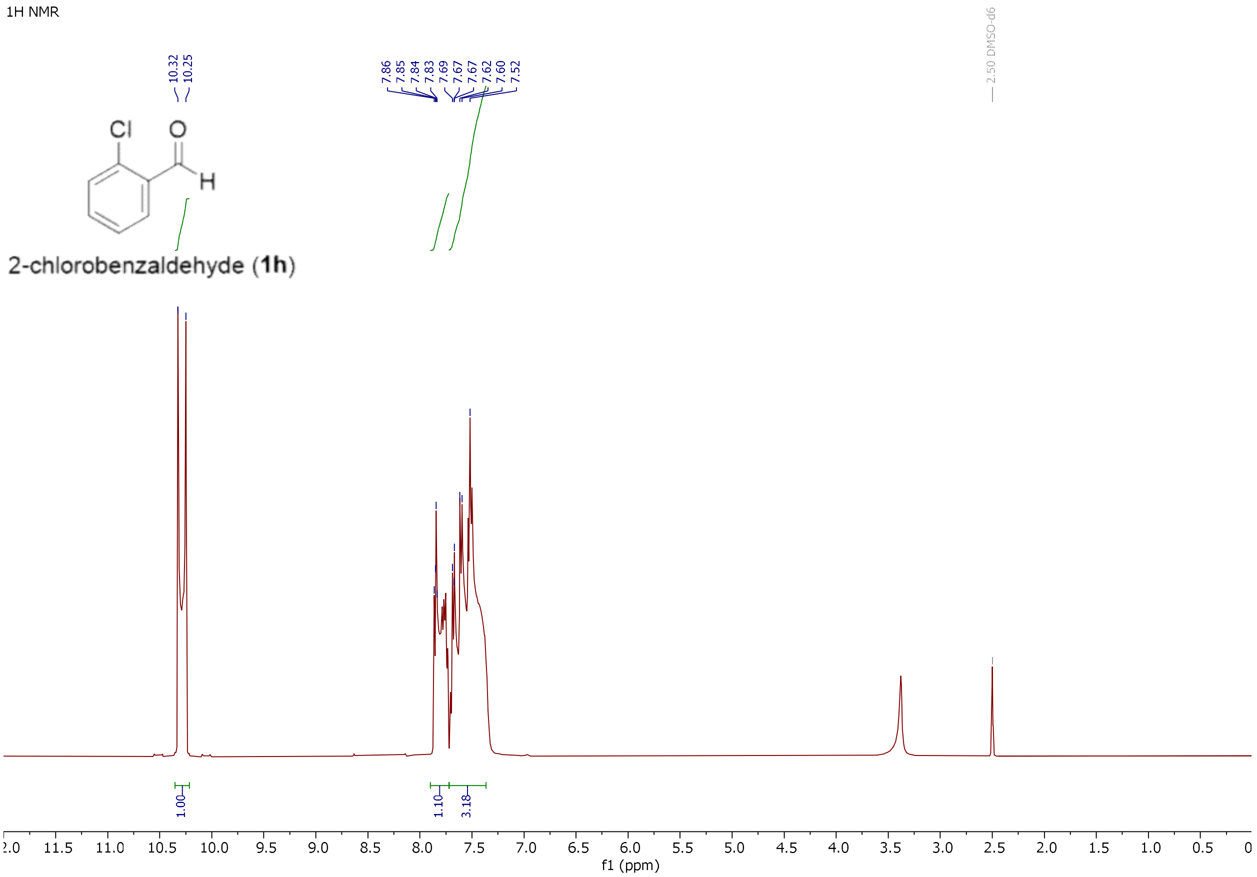

*Figure S 39 - ^{1}H NMR of **1h***

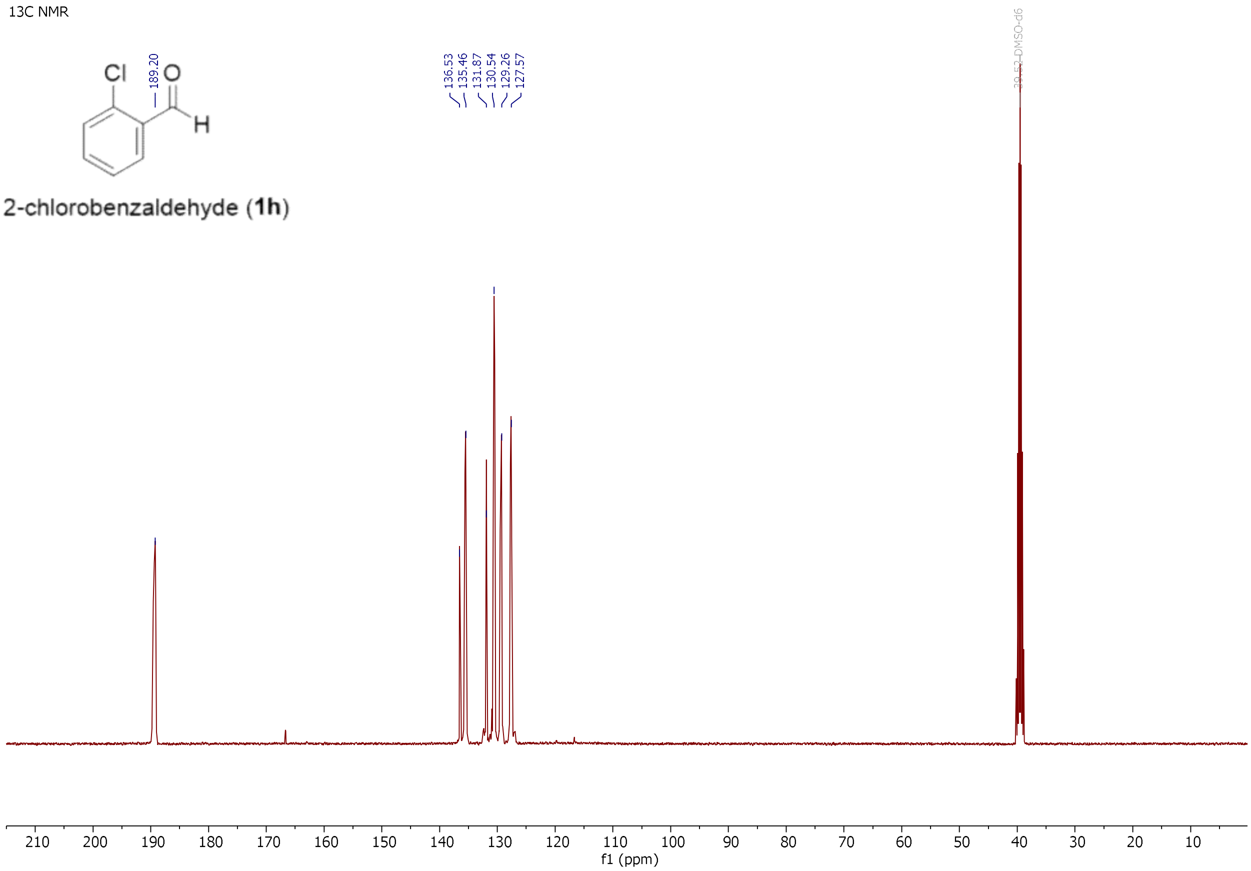

*Figure S 40 - ^{13}C NMR of **1h***

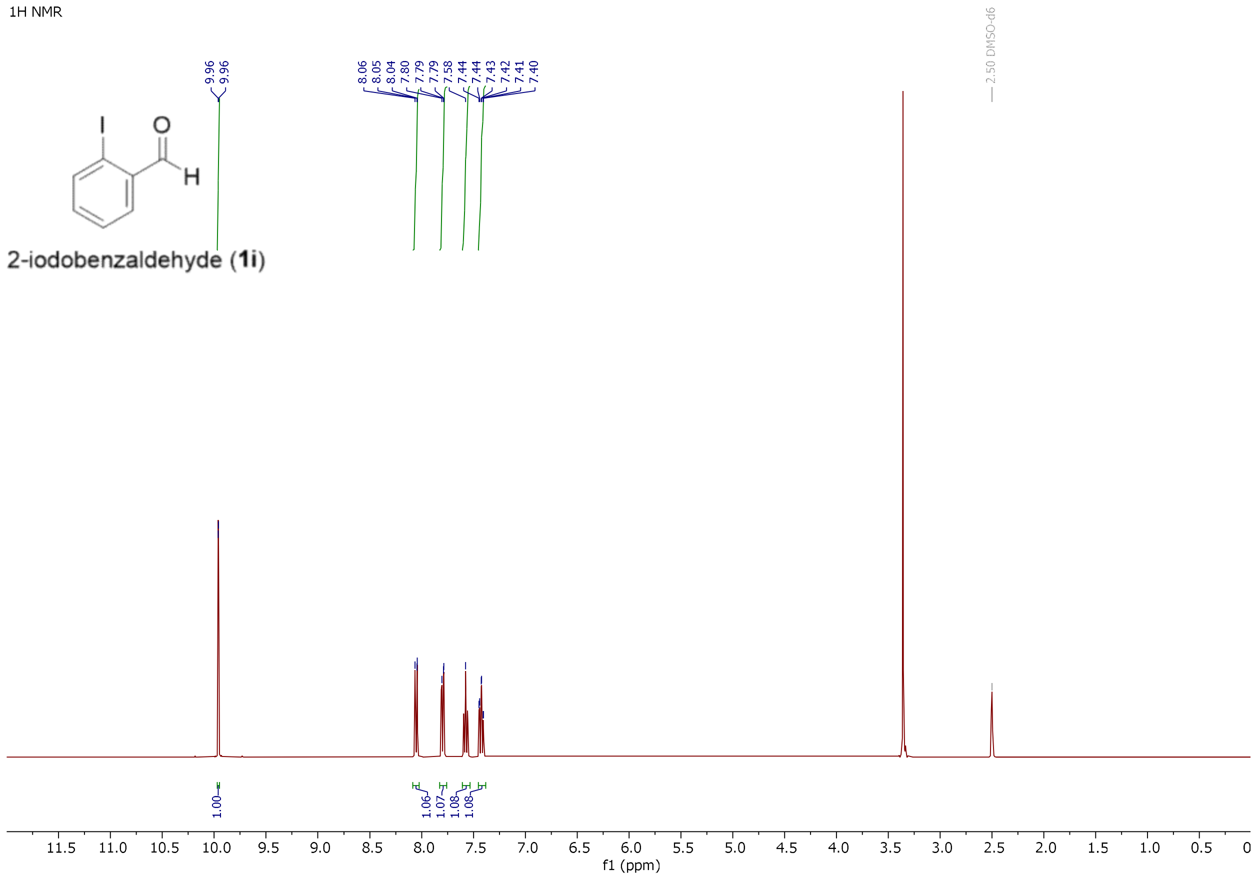

*Figure S 41 - ¹H NMR of **1i***

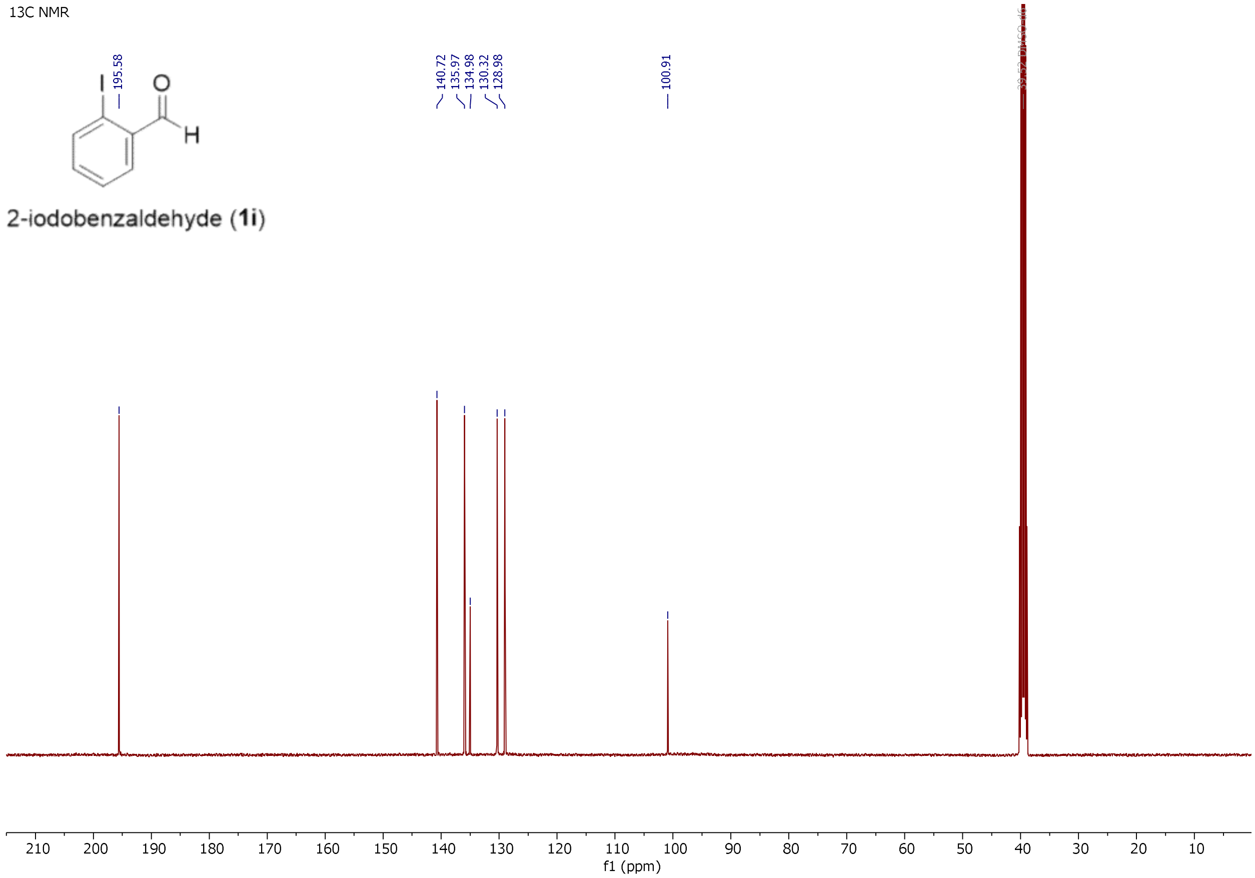

*Figure S 42 - ^{13}C NMR of **1i***

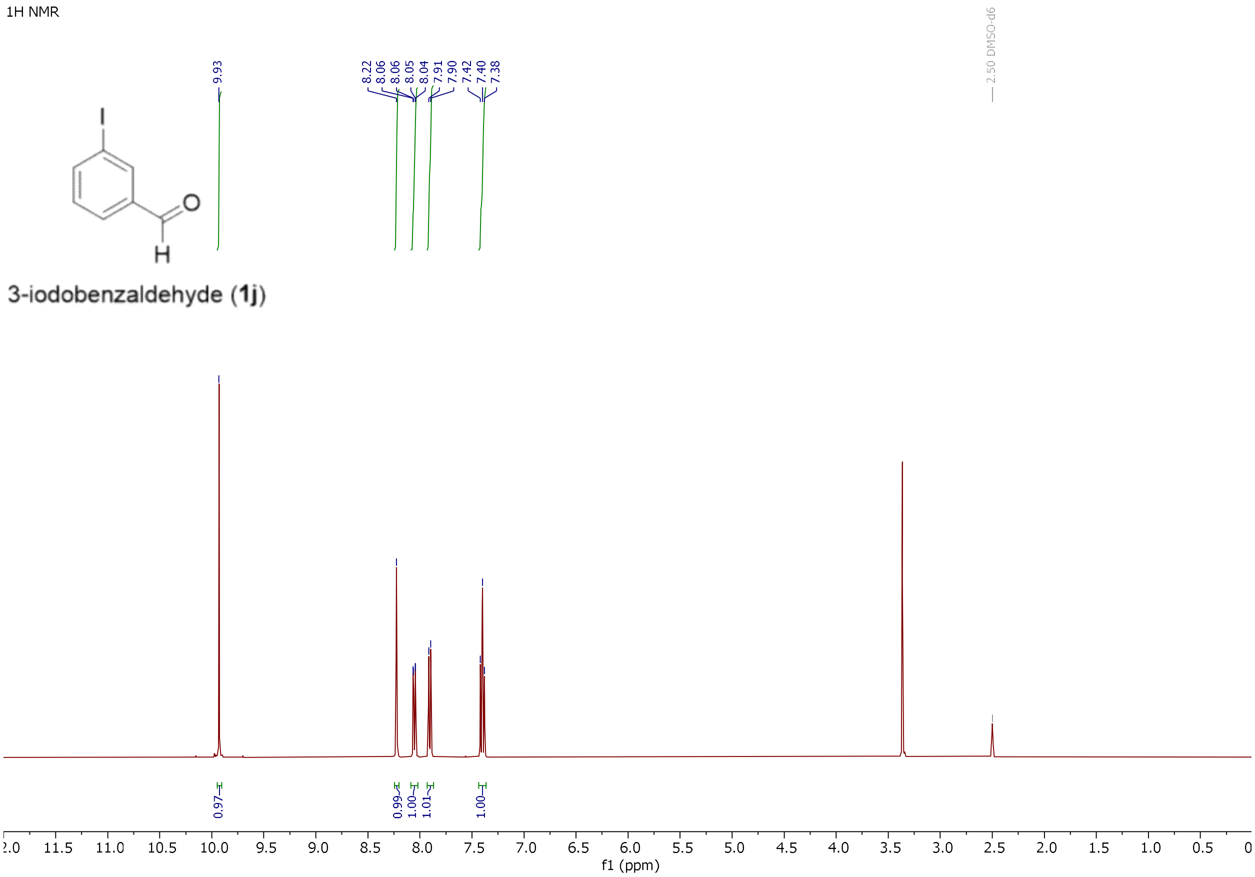

*Figure S 43 - ^{1}H NMR of **1j***

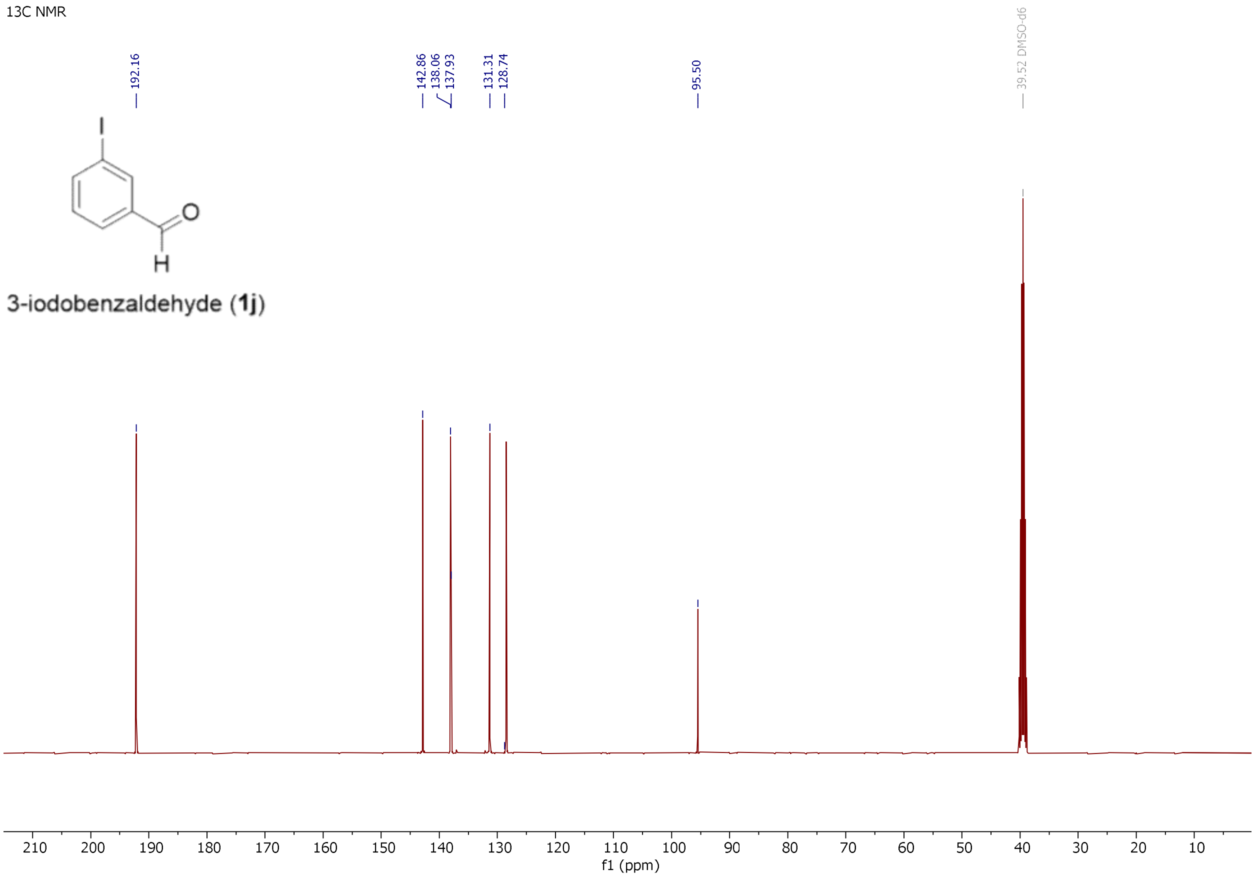

*Figure S 44 - ^{13}C NMR of **1j***

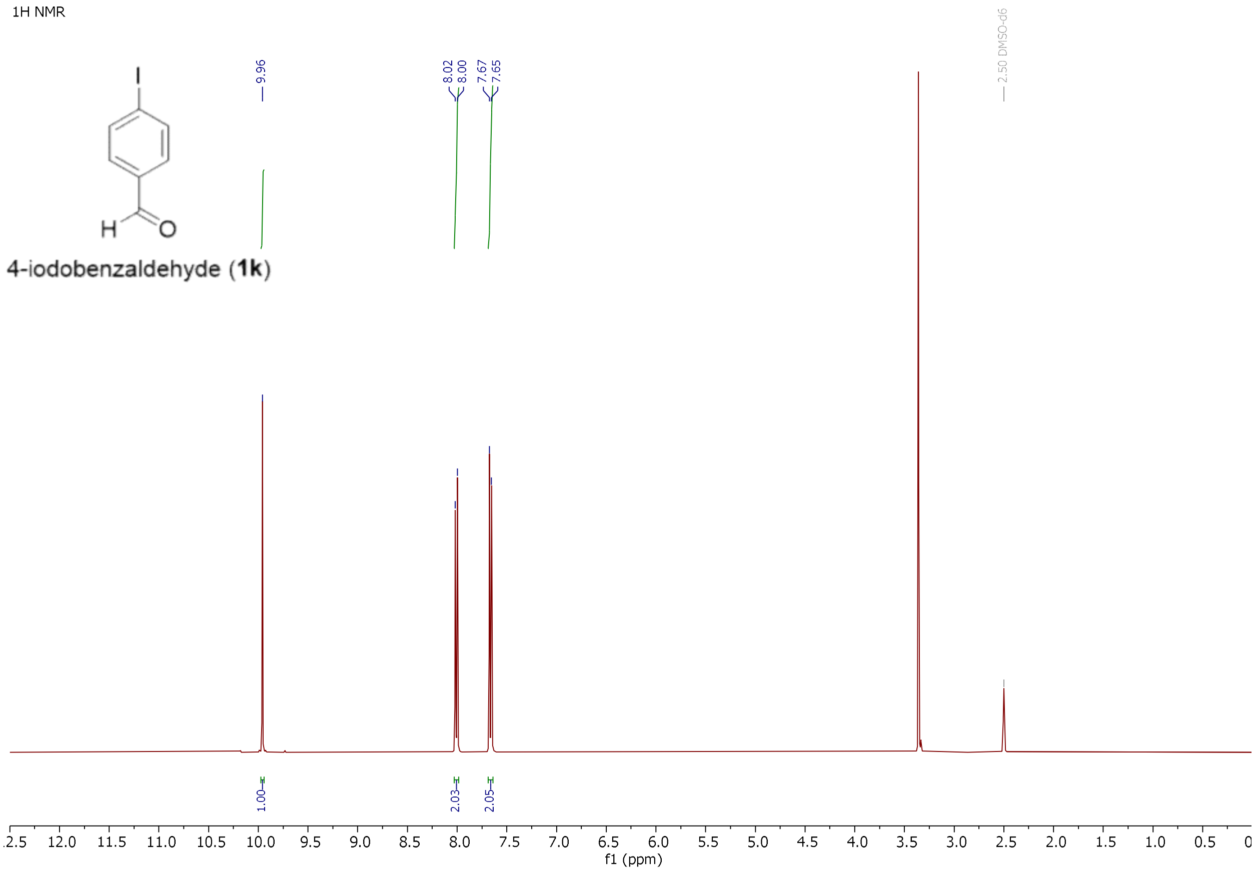

*Figure S 45 - ¹H NMR of **1k***

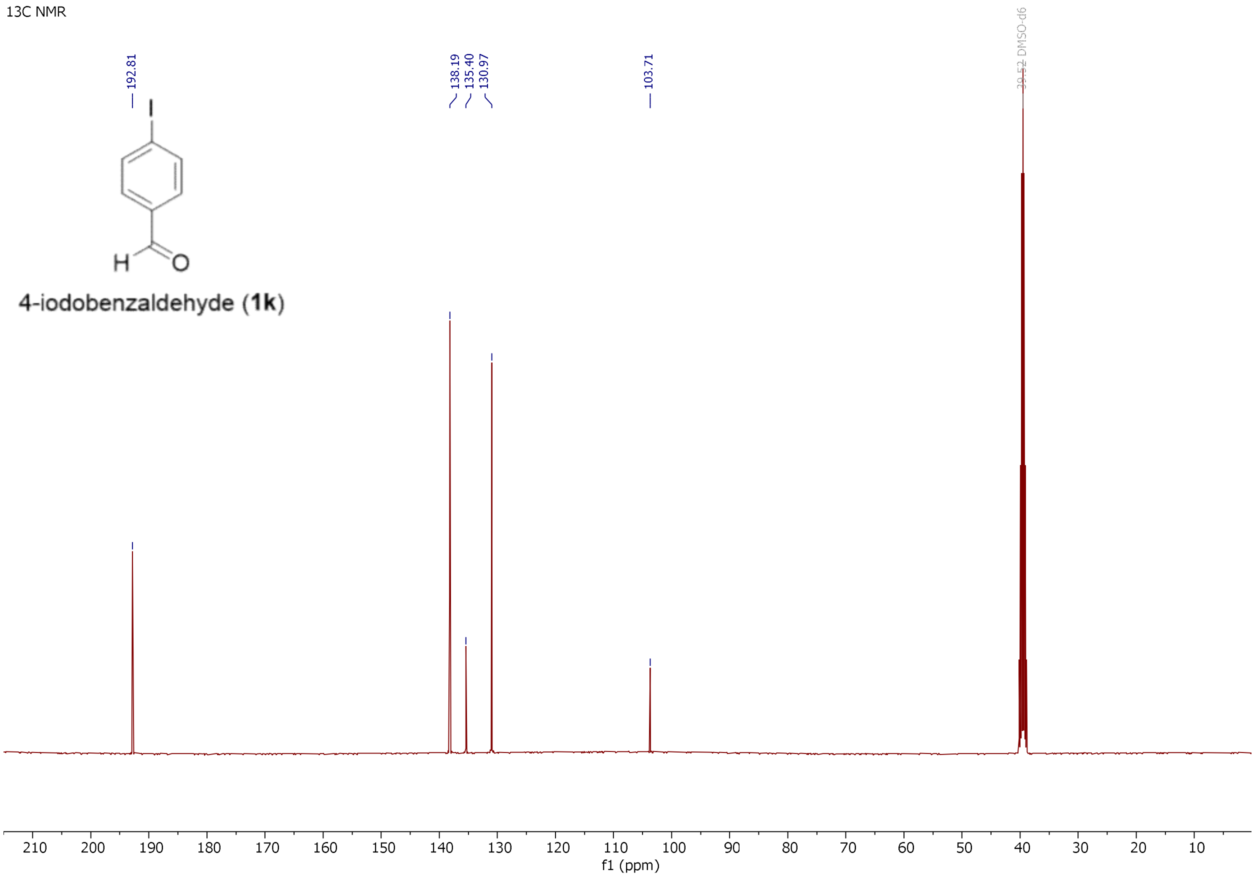

*Figure S 46 - ^{13}C NMR of **1k***

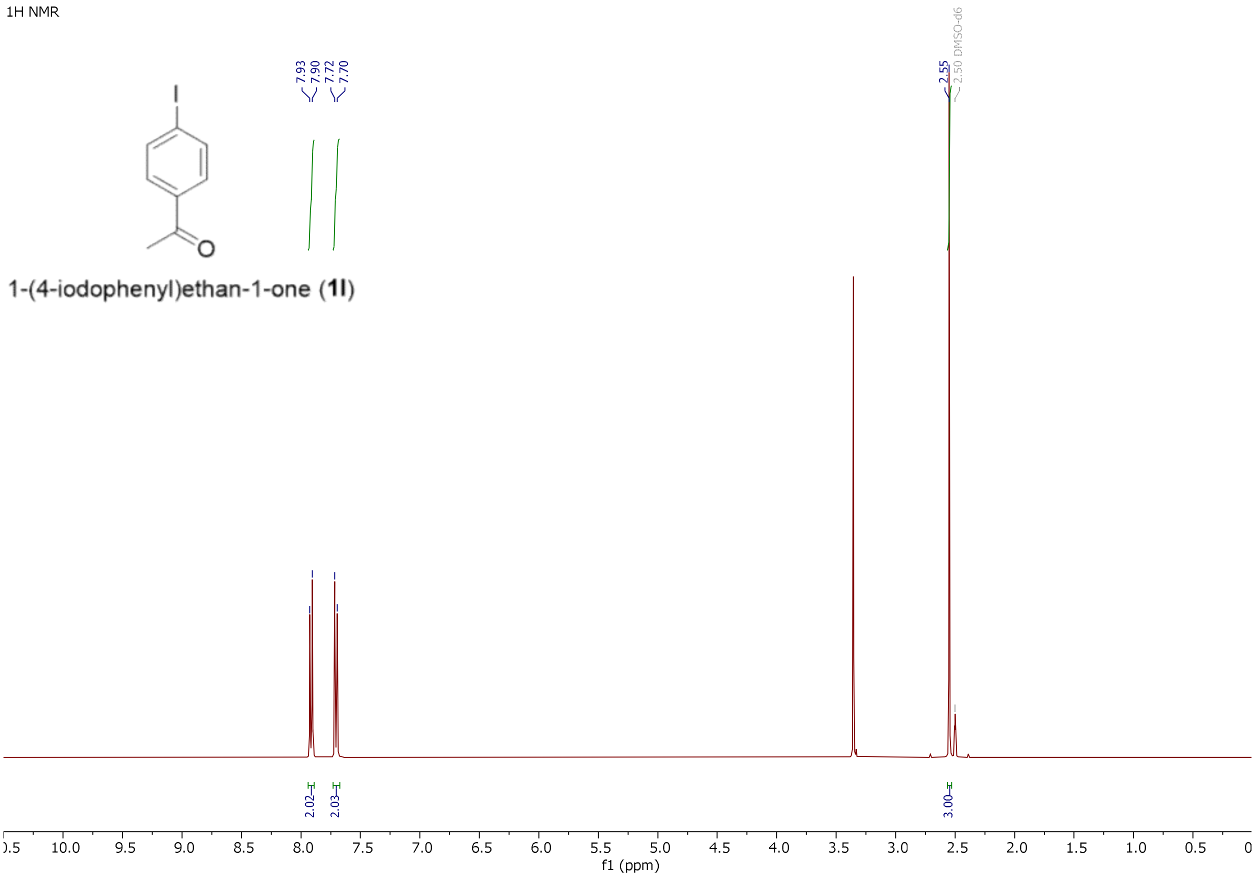

*Figure S 47 - ^{1}H NMR of **1l***

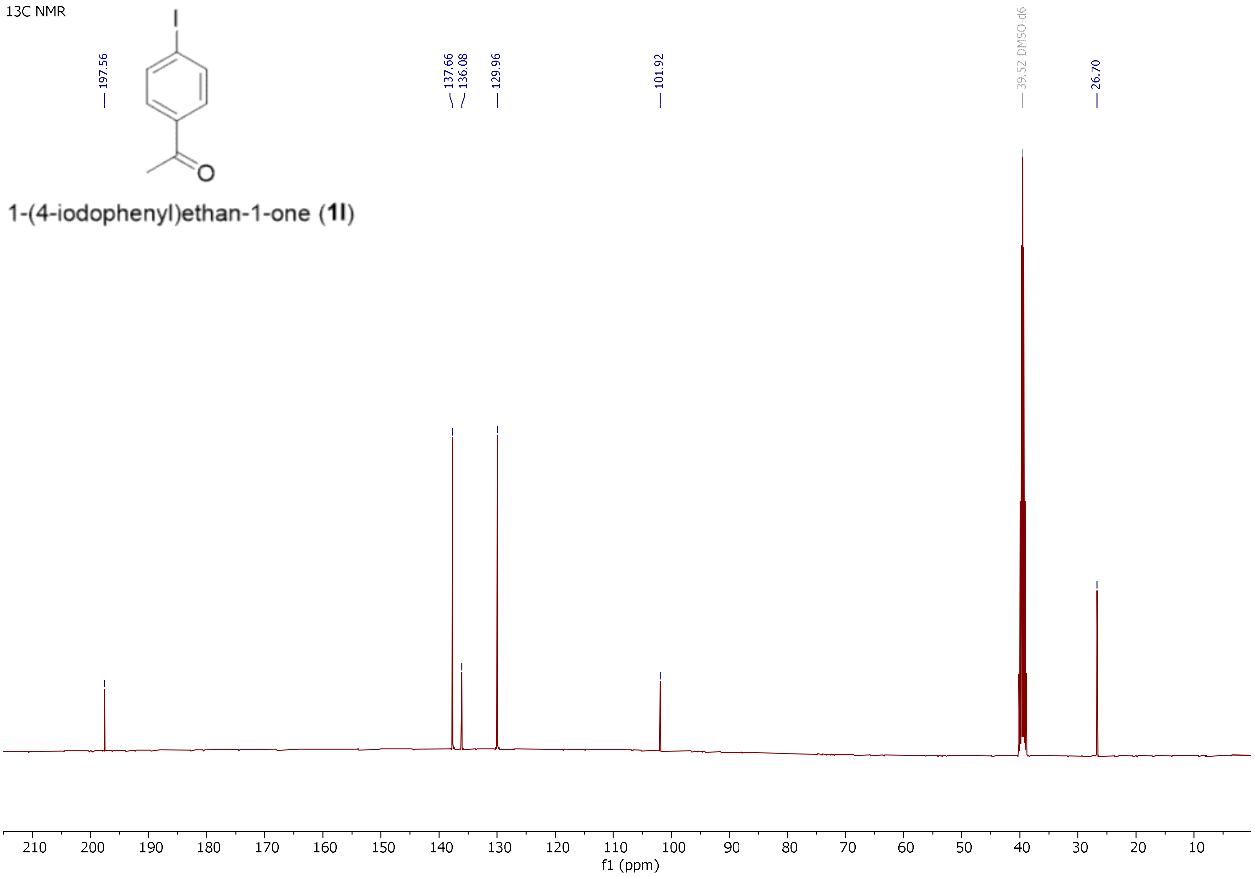

*Figure S 48 - ^{13}C NMR of **1l***

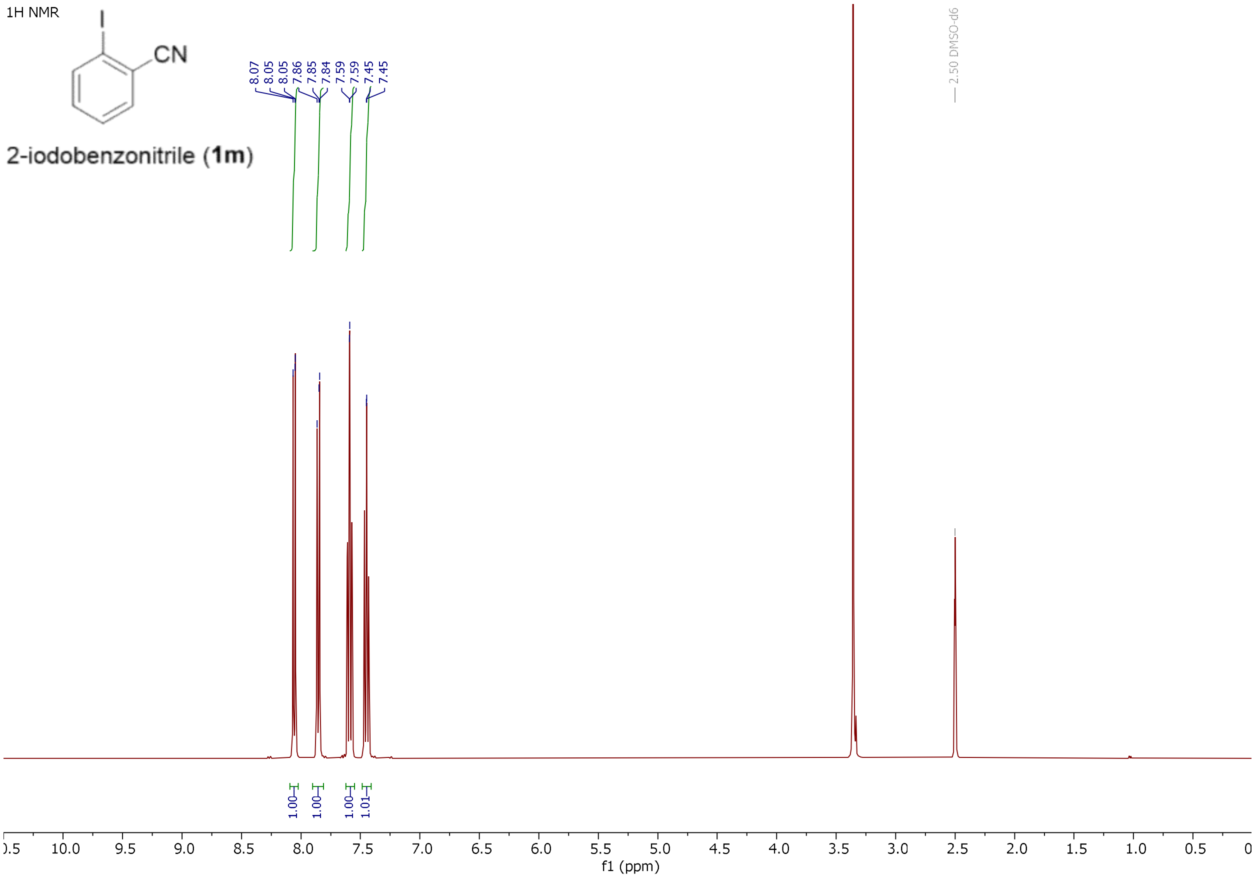

*Figure S 49 - ^{1}H NMR of **1m***

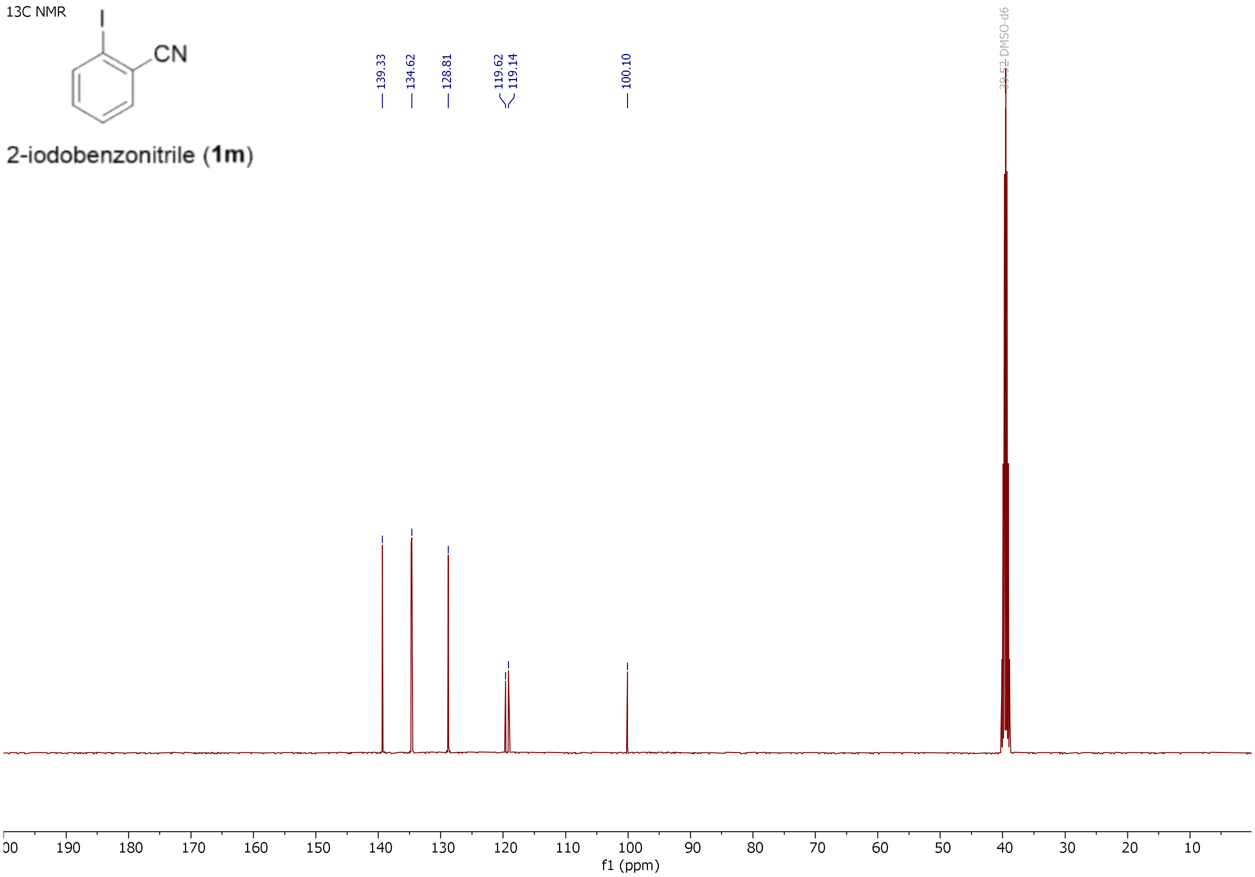

*Figure S 50 - ^{13}C NMR of **1m***

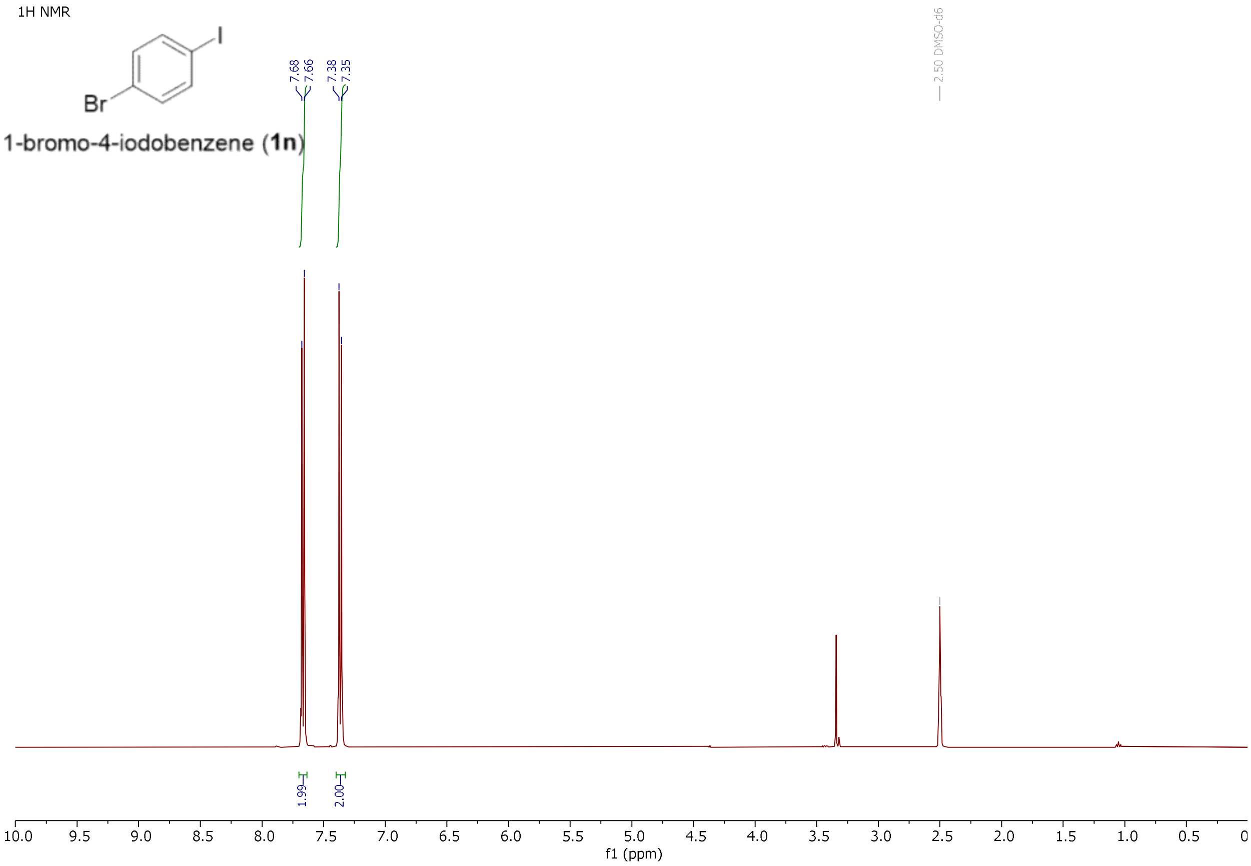

Figure S 51 - ^1H NMR of ***1n***

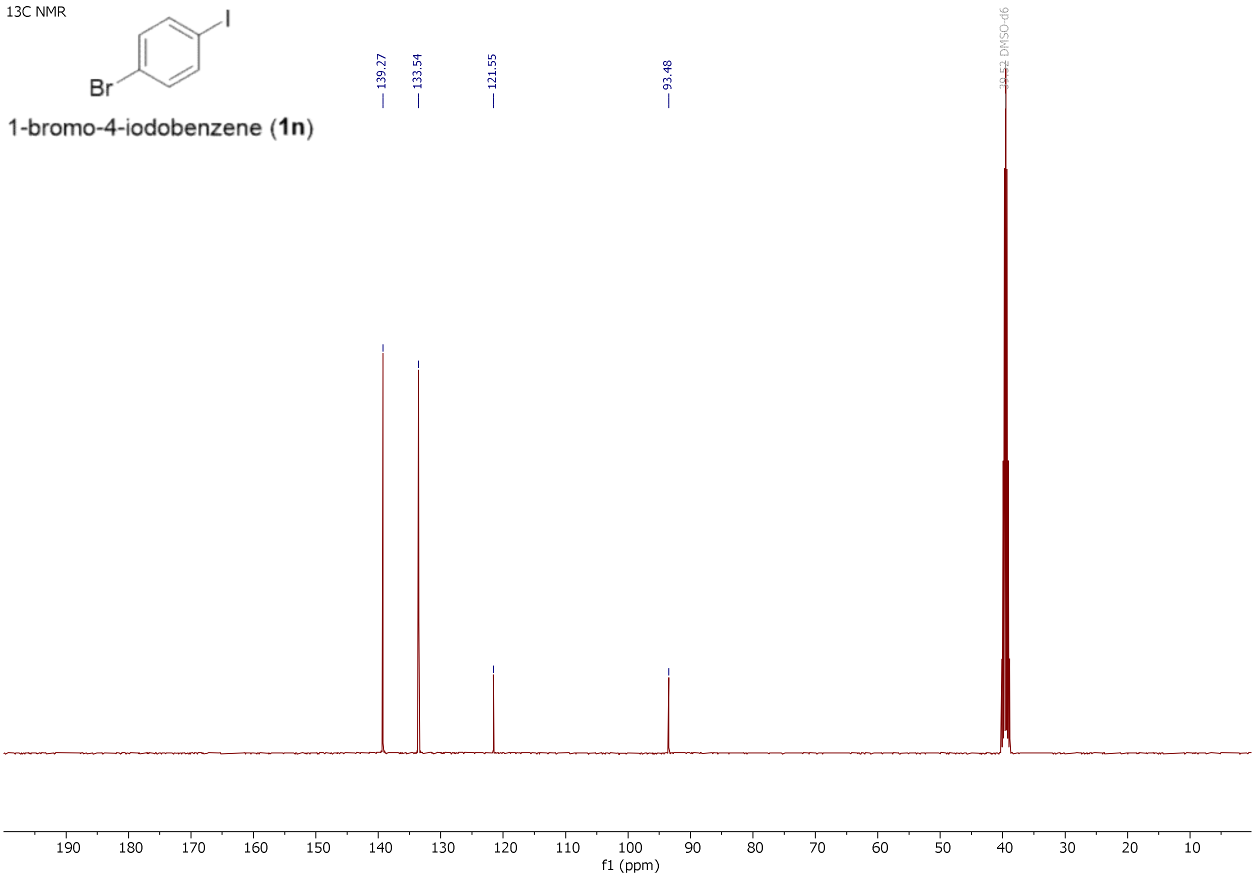

*Figure S 52 - ^{13}C NMR of **1n***

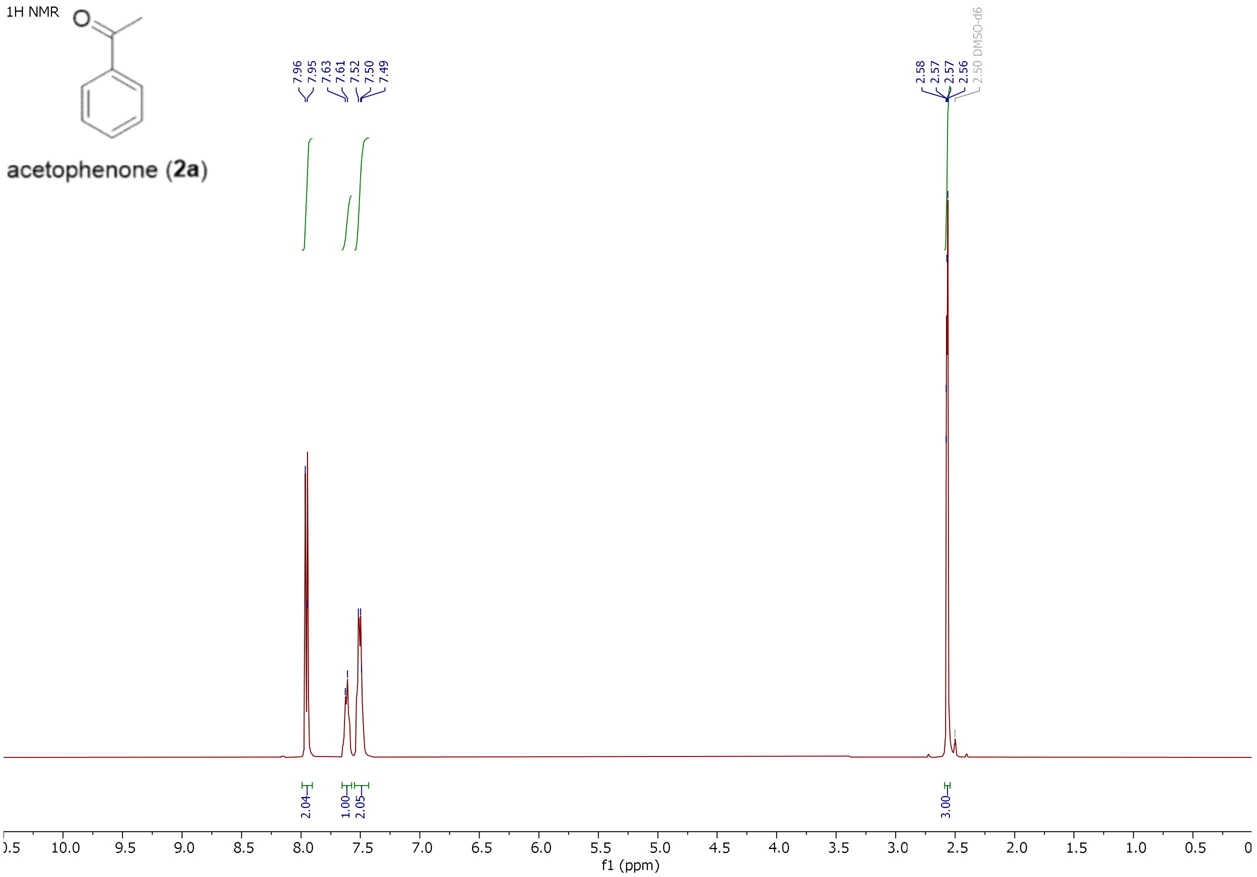

*Figure S 53 - ¹H NMR of **2a***

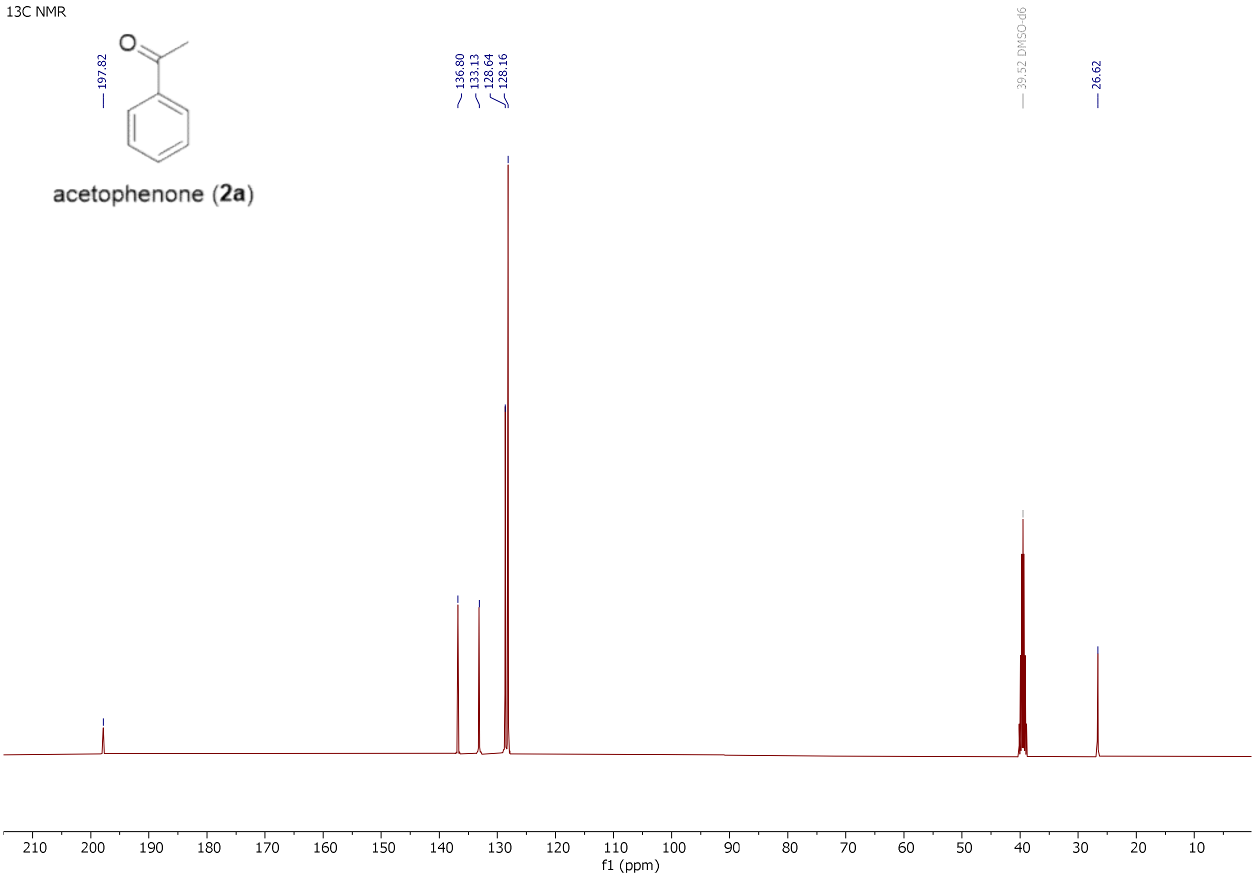

*Figure S 54 - ¹³C NMR of **2a***

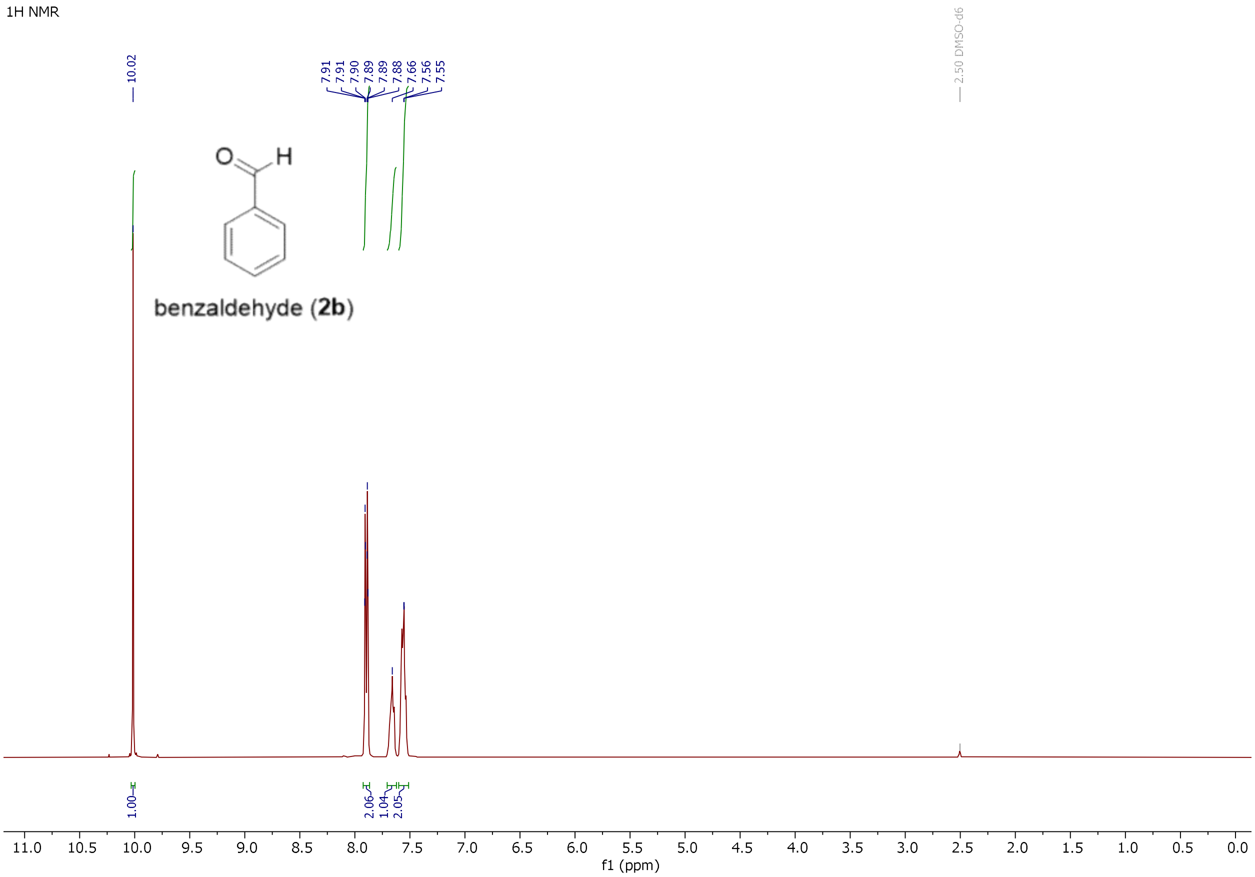

*Figure S 55 - ¹H NMR of **2b***

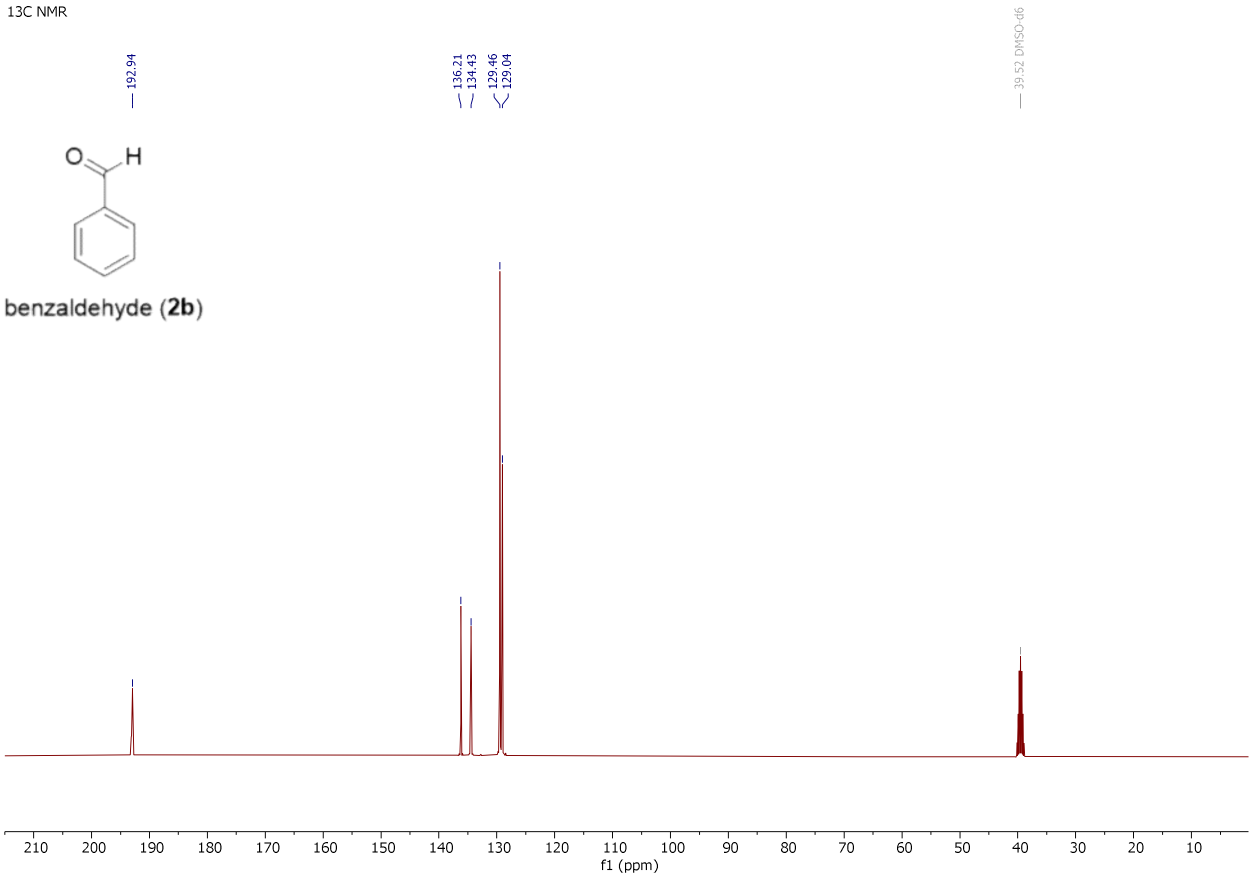

*Figure S 56 - ^{13}C NMR of **2b***

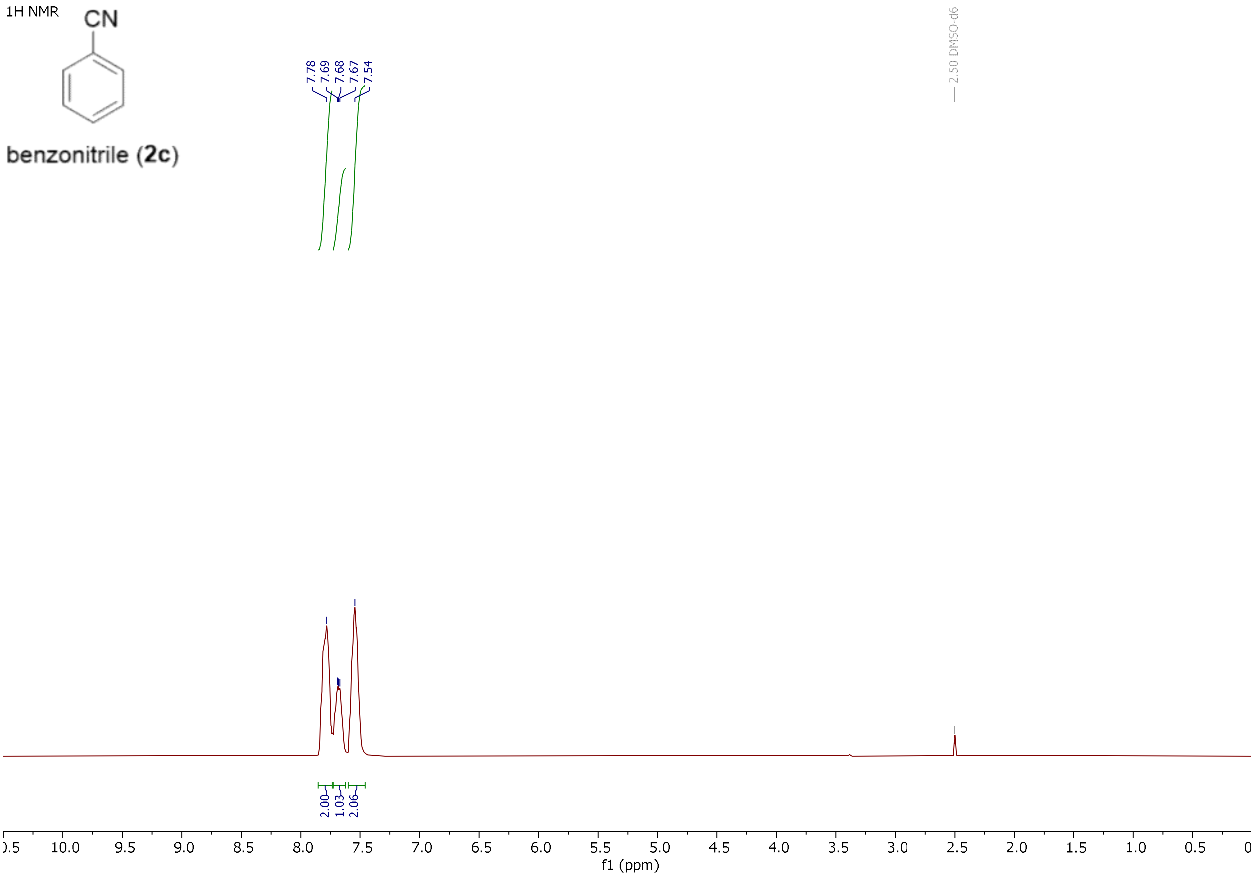

*Figure S 57 - ¹H NMR of **2c***

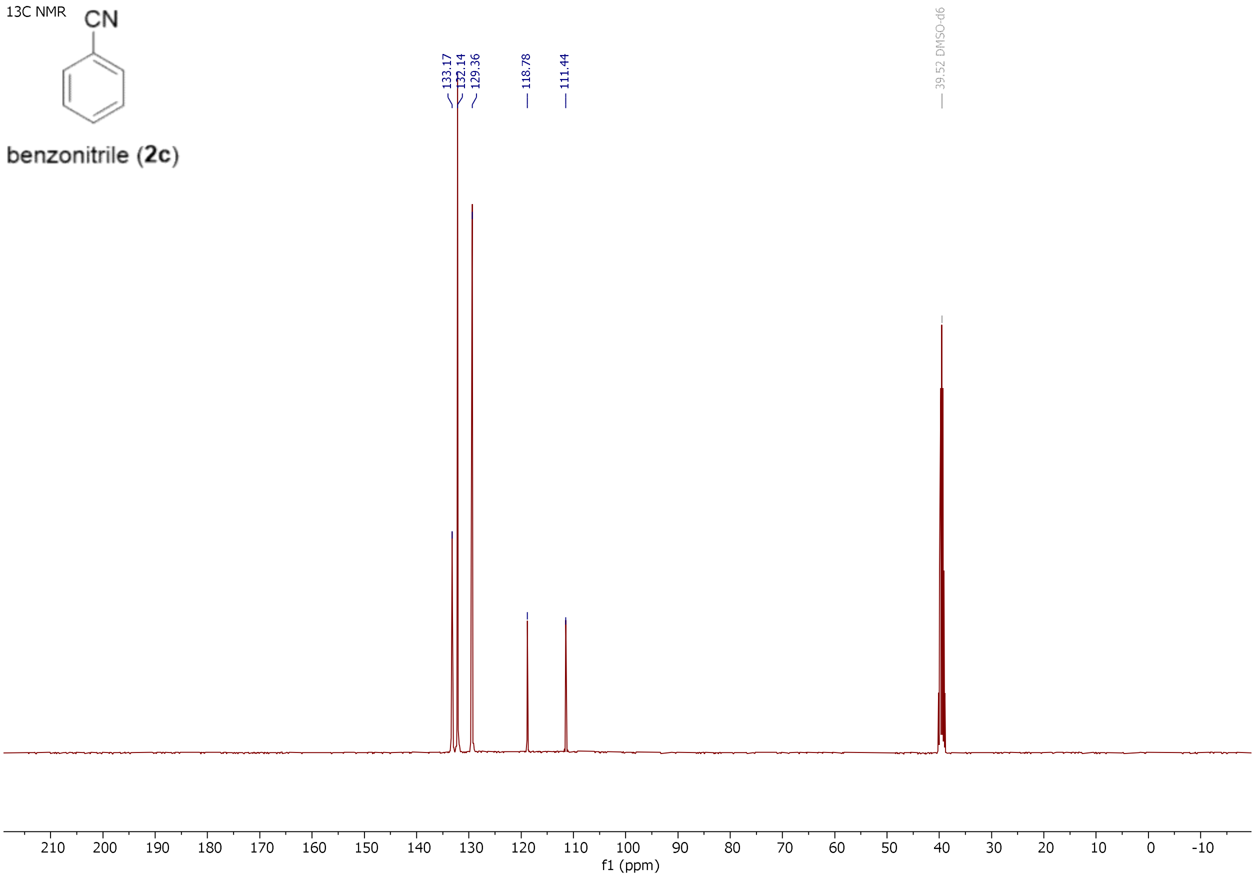

*Figure S 58 - ^{13}C NMR of **2c***

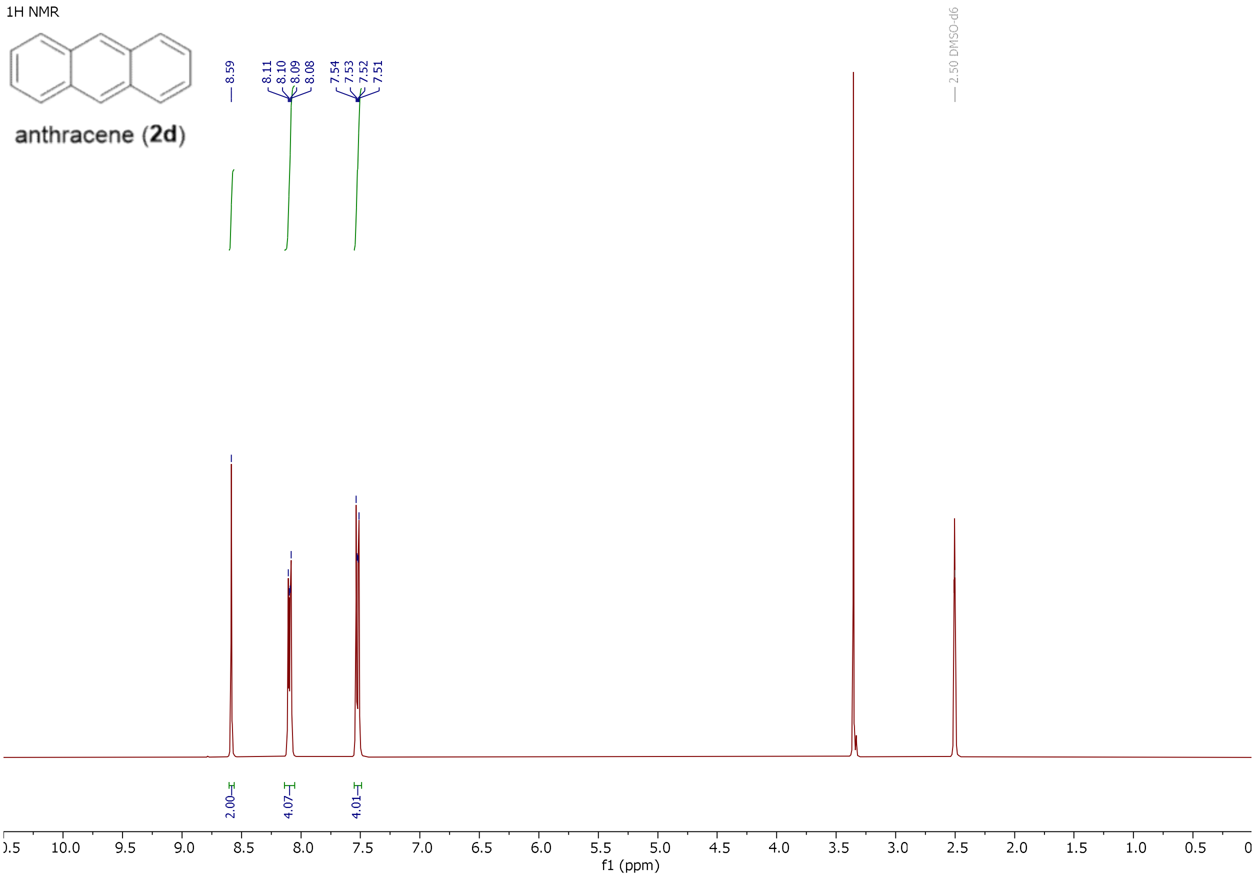

*Figure S 59 - ¹H NMR of **2d***

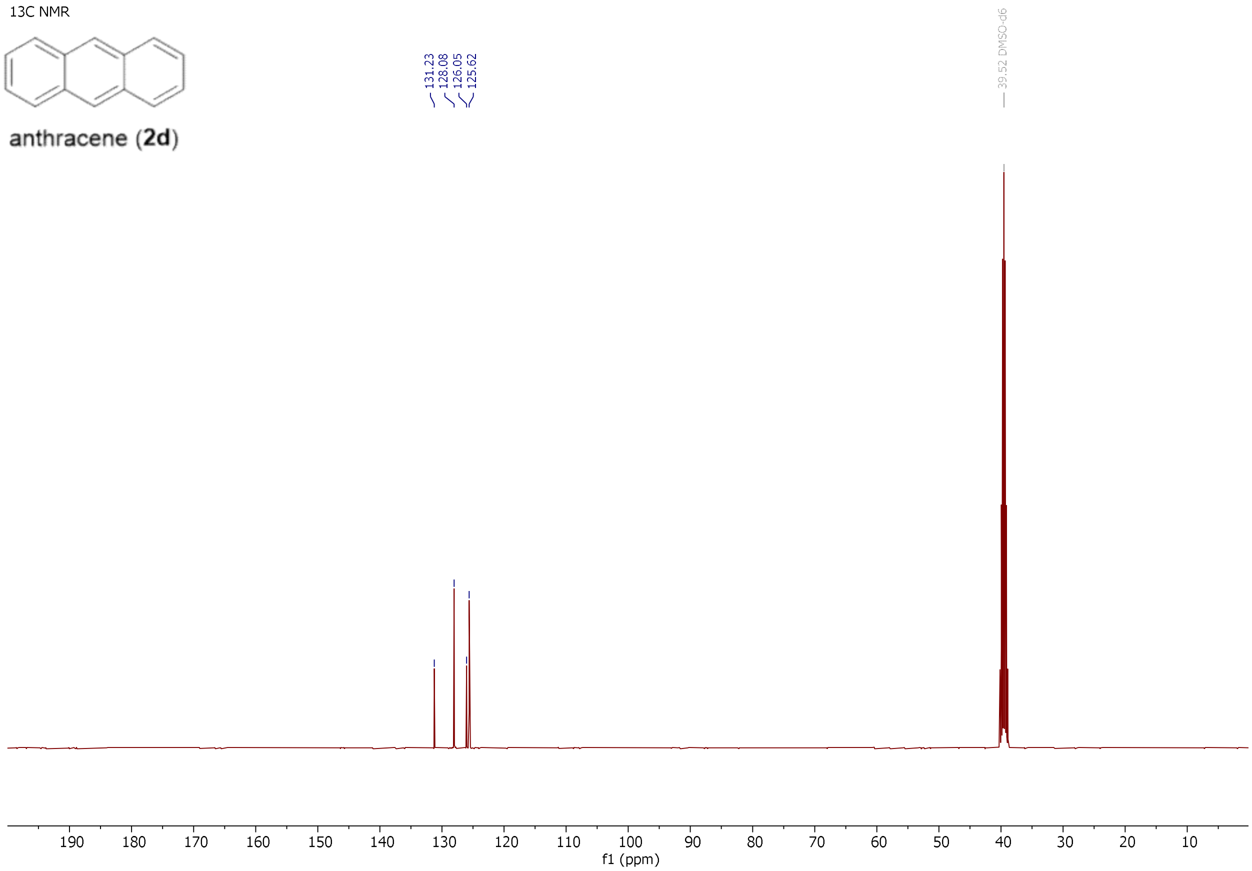

*Figure S 60 - ^{13}C NMR of **2d***

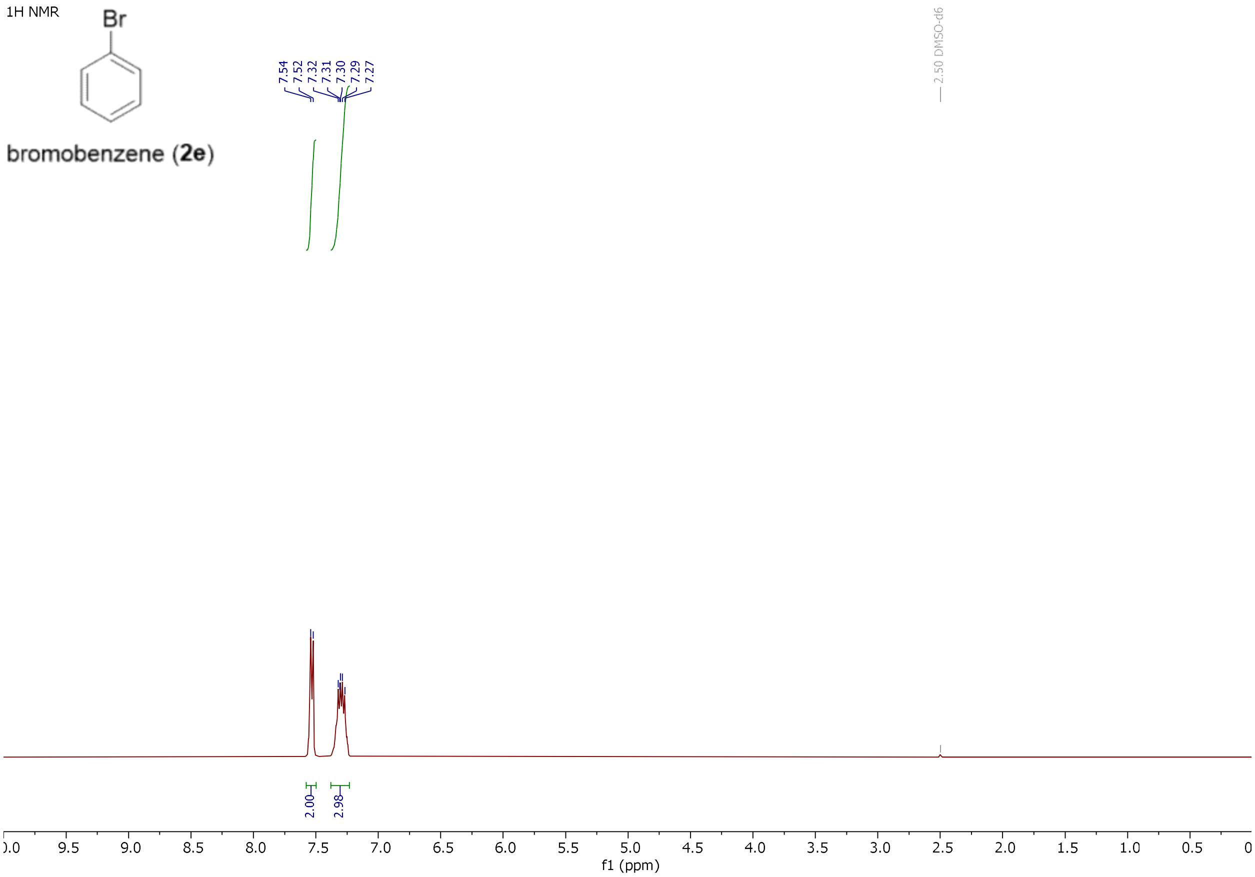

*Figure S 61 - ¹H NMR of **2e***

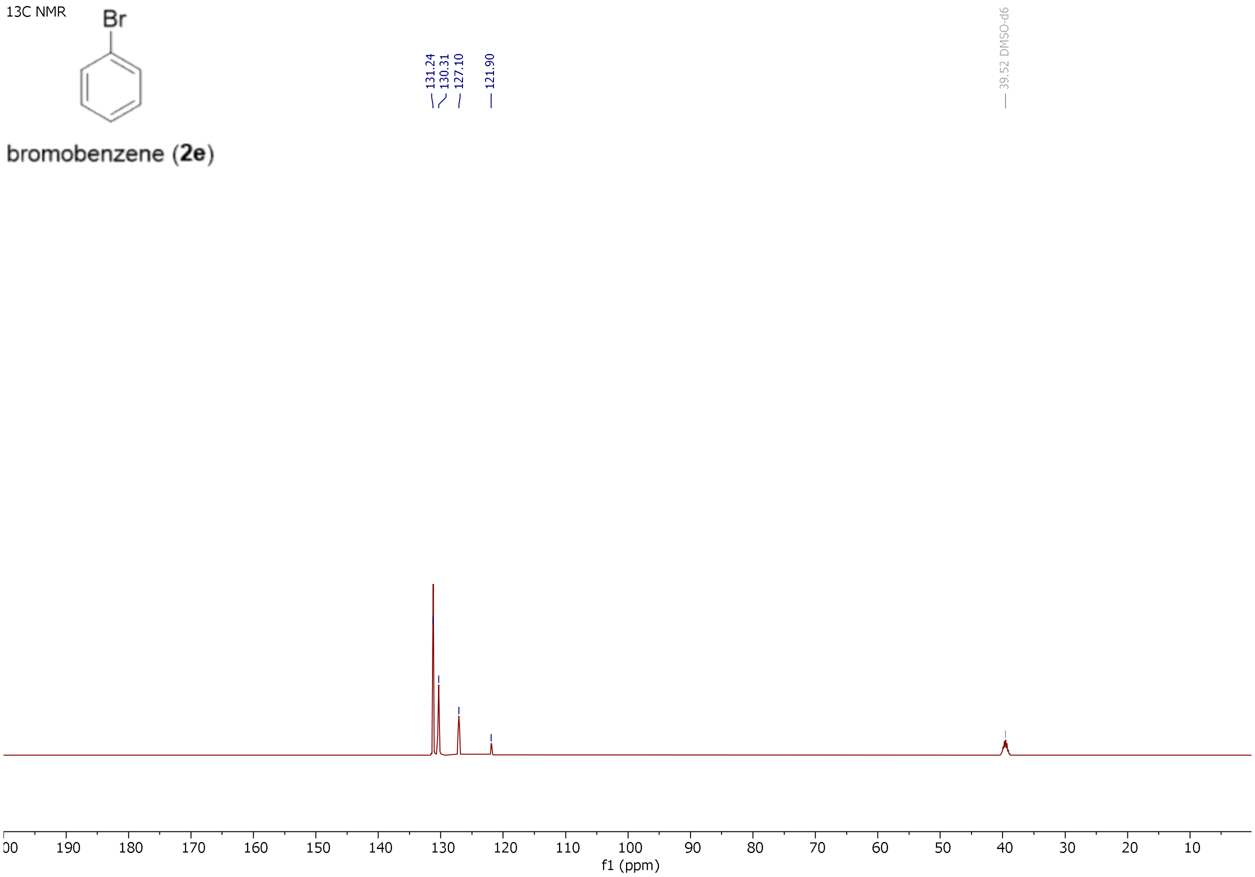

*Figure S 62 - ^{13}C NMR of **2e***

References

[1] R. J. Enemærke, T. B. Christensen, H. Jensen, K. Daasbjerg, *Journal of the Chemical Society, Perkin Transactions 2* **2001**, 1620-1630.

[2] M. Szwarc, D. Williams, *Proceedings of the Royal Society of London. Series A, Mathematical and Physical Sciences* **1953**, *219*, 353-366.

[3] C. Costentin, M. Robert, J.-M. Savéant, *Journal of the American Chemical Society* **2004**, *126*, 16051-16057.

[4] H. G. Roth, N. A. Romero, D. A. Nicewicz, *Synlett* **2016**, *27*, 714-723.

[5] K. Urgin, R. Barhdadi, S. Condon, E. Léonel, M. Pipelier, V. Blot, C. Thobie-Gautier, D. Dubreuil, *Electrochimica Acta* **2010**, *55*, 4495-4500.

[6] B. Kurpil, K. Otte, A. Mishchenko, P. Lamagni, W. Lipiński, N. Lock, M. Antonietti, A. Savateev, *Nature Communications* **2019**, *10*, 945.

[7] A. Savateev, N. V. Tarakina, V. Strauss, T. Hussain, K. ten Brummelhuis, J. M. Sánchez Vadillo, Y. Markushyna, S. Mazzanti, A. P. Tyutyunnik, R. Walczak, M. Oschatz, D. M. Guldi, A. Karton, M. Antonietti, *Angewandte Chemie International Edition* **2020**, *59*, 15061-15068.

[8] A. Savateev, I. Ghosh, B. Konig, M. Antonietti, *Angew Chem Int Ed Engl* **2018**, *57*, 15936-15947.

[9] D. Sadowsky, K. McNeill, C. J. Cramer, *Environmental Science & Technology* **2013**, *47*, 14194-14203.

[10] S. J. Blanksby, G. B. Ellison, *Accounts of Chemical Research* **2003**, *36*, 255-263.

[11] H. Ou, C. Tang, X. Chen, M. Zhou, X. Wang, *ACS Catalysis* **2019**, *9*, 2949-2955.

[12] Z. Zeng, X. Quan, H. Yu, S. Chen, Y. Zhang, H. Zhao, S. Zhang, *Applied Catalysis B: Environmental* **2018**, *236*, 99-106.

[13] J. J. Warren, T. A. Tronic, J. M. Mayer, *Chemical Reviews* **2010**, *110*, 6961-7001.

[14] B. Kurpil, Y. Markushyna, A. Savateev, *ACS Catalysis* **2019**, *9*, 1531-1538.

[15] S. Shi, R. Szostak, M. Szostak, *Organic & Biomolecular Chemistry* **2016**, *14*, 9151-9157.

[16] https://www.americanelements.com/graphitic-carbon-nitride (accessed: July 2021).

[17] Y. Markushyna, P. Lamagni, C. Teutloff, J. Catalano, N. Lock, G. Zhang, M. Antonietti, A. Savateev, *Journal of Materials Chemistry A* **2019**, *7*, 24771-24775.

[18] R. H. Joel N. Schrauben, Carolyn N. Valdez, Miles Braten, Lila Fridley, James M. Mayer, *Science* **2012**, *336*, 1298-1301.

[19] W. K. Liu, K. M. Whitaker, A. L. Smith, K. R. Kittilstved, B. H. Robinson, D. R. Gamelin, *Physical Review Letters* **2007**, *98*, 186804.

[20] C. N. Valdez, M. F. Delley, J. M. Mayer, *Journal of the American Chemical Society* **2018**, *140*, 8924-8933.
